# Supplementary material for: Camrelizumab combined with apatinib in patients with first-line platinum-resistant or PD-1 inhibitor resistant recurrent/metastatic nasopharyngeal carcinoma: a single-arm, phase 2 trial
Source: Nat Commun. 2023 Aug 14;14:4893. doi: 10.1038/s41467-023-40402-x (PMC10425437; doi:10.1038/s41467-023-40402-x)
Supplement: Supplementary file 1 — Supplementary Information [file 41467_2023_40402_MOESM1_ESM.pdf]

Supplementary information

Contents

Supplementary Tables **1**

Supplementary Table 1: Baseline characteristics 3

Supplementary Table 2: Efficacy and survival in full analysis set and efficacy analysis set (before the protocol amendment) 5

Supplementary Table 3: Efficacy and survival in full analysis set and efficacy analysis set (after the protocol amendment) 6

Supplementary Table 4: Cumulative acute adverse events in full analysis set (n=72) 7

Supplementary Table 5: Cumulative acute adverse events in cohort 2 during the neoadjuvant phase (n=32) 8

Supplementary Table 6: Biomarker and subgroup analysis for correlation with clinical efficacy in all 72 patients. 9

Supplementary Table 7: Results of subgroup analysis of PFS and OS in all 72 patients 11

Supplementary Table 8: Characteristics of patients who developed nasopharynx necrosis 13

Supplementary Table 9: Clinical characteristics of patients with tumor specimens underwent RNA-Seqs analysis 14

Supplementary Table 10: Univariate and multivariate logistic regression analysis predicting response to camrelizumab plus apatinib in cohort 1 using cell population signatures at baseline. 16

Supplementary Table 11: Enrichment scores of gene signatures in baseline tumor specimens in cohort 2. 17

Supplementary Table 12: Detailed clinical information of 98 LA-NPC patients for RNA-Seq validation. 18

Supplementary Table 13: MCP-counter outputs at baseline in 98 LA-NPC patients for RNA-Seq validation. 21

Supplementary Table 14: Summary of clinical endpoints of included trials. 25

Supplementary Table 15: Gene signatures representing potentially clinically relevant pathways from RNA sequencing data in cohort 2. 26

Supplementary Figure 1: Flow chart of the study. 27

Supplementary Figure 2: The stability of objective response rate (ORR) as the sample size increased. 28

Supplementary Figure 3: Radiological images of a representative patient in cohort 1. 29

Supplementary Figure 4: IHC of PD-L1 expression 30

Supplementary Figure 5: KDR expression in different cohort. 31

Supplementary Figure 6: C-KIT and SRC expression. 32

Supplementary Figure 7: Plasma EBV DNA titer fold reduction from baseline. 33

Supplementary Figure 8: Schematic diagram of longitudinal tumor samples. 34

Supplementary Figure 9: Gene expression characteristics and enrichment in cohort 1. 35

Supplementary Figure 10: B cell is associated with prognosis of patients with nasopharyngeal carcinoma 36

Supplementary Figure 11: Tertiary lymphatic structure and spatial distance analysis. 37

Supplementary Figure 12: Transcriptional analysis of tumor specimens from patients in different cohorts at baseline. 39

Supplementary Figure 13: Blood vessel markers (VEGFA and IL8) expression. 40

Supplementary Figure 14: PD-L1 was detected on vascular endothelium. 41

Supplementary Figure 15: CD8 and blood vessel markers expression. 42

Supplementary Figure 16: Transcriptional analysis of the difference of tumor specimens between baseline and post-treatment in responders and non-responders. 43

Supplementary Figure 17: Efficacy of anti-PD-1 or apatinib monotherapy, and our combination therapy (cohort 1) in RM-NPC. 45

Supplementary References 46

Supplementary Note 1. Study Protocol 47

Supplementary Table 1: Baseline characteristics

Characteristic, n (%)	All patients (n=72)	Cohort 1(n=40)	Cohort 2(n=32)
Gender			
Male	56 (77.8)	32(80.0)	24(75.0)
Female	16 (22.2)	8(20.0)	8(25.0)
Age, median (IQR)	45 (36-54)	49 (37-54)	40(36-50)
Histology			
WHO II	4(5.6)	3(7.5)	1(3.1)
WHO III	68 (94.4)	37(92.5)	31(96.9)
ECOG score			
0	34 (47.2)	19(47.5)	15(46.9)
1	38 (52.8)	21(52.5)	17(53.1)
Recurrence and/ or metastasis			
Recurrence	4 (5.6)	1(2.5)	3(9.3)
Metastasis	56 (77.8)	30(75.0)	26(81.3)
Recurrence & metastasis	12 (16.7)	9(22.5)	3((9.3))
Location of metastases			
Liver	37 (51.4)	20(50.0)	17(53.1)
Lung	32 (44.4)	18(45.0)	14(43.8)
Bone	22 (30.6)	11(27.5)	11(34.4)
Others	38 (52.8)	21(52.5)	17(53.1)
Multiple organs involved	43 (59.7)	25(62.5)	18(56.3)
Plasma EBV DNA level(copies/ml)			
≥ 10,000	25(34.7)	14(35.0)	11(34.4)
< 10,000	47(65.3)	26(65.0)	21(65.6)
Target lesion size, median (IQR) (mm)	26 (9-30)	18 (8-26)	35 (11-42)
Previous lines of therapy for advanced disease			
1	38 (52.8)	24(60.0)	14(43.8)
2	27 (37.5)	15(37.5)	12(37.5)
≥ 3	7 (9.7)	1(2.5)	6(18.8)
Smoking history			
Yes	25 (34.7)	13(32.5)	12(37.5)
No	47 (65.3)	27(67.5)	20(62.5)
Previous radiotherapy			
Yes	45(62.5)	24(60.0)	21(65.6)
No	27(37.5)	16(40.0)	11(34.4)
Previous chemotherapy drug for advanced disease			
Cisplatin	70(97.2)	40(100.0)	30(93.8)
Paclitaxel	26(36.1)	15(37.5)	11(34.4)

Gemcitabine	44(61.1)	22(55.0)	22(68.8)
5-fluorouracil	28(38.9)	21(52.5)	7(21.9)
Previous PD-1 inhibitor for advanced disease			
Camrelizumab	/	/	8(25.0)
Toripalimab	/	/	15(46.9)
Sintilimab	/	/	3(9.4)
Nivolumab	/	/	3(9.4)
Anti-CTLA4 and Anti-PD1	/	/	3(9.4)
PD-L1 status (TPS)			
Negative	14(19.4)	6(15.0)	8(25.0)
1%-50%	28(38.9)	14(35.0)	14(43.8)
50%-100%	10(13.9)	9(22.5)	1(3.1)
Unknown	20(27.8)	11(27.5)	9(28.1)
PD-L1 status (CPS)			
Negative	8(11.1)	3(7.5)	5(15.6)
Positive	44(61.1)	26(65.0)	18(56.3)
Unknown	20(27.8)	11(27.5)	9(28.1)
KDR status			
Negative	16(22.2)	8(20.0)	8(25.0)
Positive	36(50.0)	21(52.5)	15(46.9)
Unknown	20(27.8)	11(27.5)	9(28.1)
Previous EGFR inhibitor treatment			
Nimotuzumab/Cetuximab	18(25.0)	11(27.5)	7(21.9)
No	54(75.0)	29(72.5)	25(78.1)

Abbreviations: ECOG=Eastern Cooperative Oncology Group; EBV DNA: Epstein-Barr virus (EBV) DNA; EGFR, epidermal growth factor receptor; KDR, vascular endothelial growth factor receptor 2.

、

Supplementary Table 2: Efficacy and survival in Full analysis set and Efficacy analysis set (before the protocol amendment)

	Full analysis set (n=52)		Efficacy analysis set (n=48)	
	**Cohort 1(n=27)**	**Cohort 2(n=25)**	**Cohort 1(n=26)**	**Cohort 2(n=22)**
**Overall response**				
CR	1 (3.7)	NE	1 (3.8)	NE
PR	17(63.0)	10(40.0)	17(65.4)	10(45.5)
SD	4 (14.8)	8(32.0)	4 (15.4)	8(36.4)
PD	4 (14.8)	4(16.0)	4 (15.4)	4(18.2)
Non-evaluable*	1(3.7)	3(12.0)	NE	NE
**ORR**	66.7(47.7-85.7)	40.0(19.4-60.6)	69.2(50.2-88.2)	45.5(22.9-68.1）
**DCR**	81.5(65.8-97.1)	72.0(53.1-90.9)	84.6(69.8-99.5)	81.8(64.3-99.3）

Data are n (%) or n (%, 95% CI)

Efficacy and survival are listed in Full analysis set (n=52) and Efficacy analysis set (n=48), respectively. Response was assessed according to the RECIST v1.1. Only confirmed responses were listed.

Abbreviations: CR, complete response; PR, partial response; SD, stable disease; PD, progressive disease; ORR, objective response rate; DCR, disease control rate.

Supplementary Table 3: Efficacy and survival in Full analysis set and Efficacy analysis set (after the protocol amendment)

	Full analysis set (n=72)		Efficacy analysis set (n=64)	
	**Cohort 1(n=40)**	**Cohort 2(n=32)**	**Cohort 1(n=36)**	**Cohort 2(n=28)**
**Overall response**				
CR	3 (7.5)	NE	3(8.3)	NE
PR	23(57.5)	11(34.4)	23(63.9)	11(39.3)
SD	6 (15.0)	11(34.4)	6 (16.7)	11(39.3)
PD	4 (10.0)	6(18.8)	4 (11.1)	6(21.4)
Non-evaluable*	4(10.0)	4(12.5)	NE	NE
**ORR**	65.0(49.6-80.4)	34.4(17.0-51.8)	72.2(56.9-87.6)	39.3(20.0-58.6）
**DCR**	80.0(67.0-93.0)	68.8(51.8-85.7)	88.9(78.1-99.7)	78.6(62.4-94.8）
**Progression-free survival**				
Median, months	12.6(1.5-23.7)	4.5(3.7-5.4)	13.4(9.4-17.5)	4.5(3.5-5.6)
6-month	65.0(50.3-79.7)	37.5(20.6-54.4)	66.7(51.2-82.2)	35.7(17.9-53.5)
12-month	52.5(37.0-68.0)	18.8(5.3-32.3)	55.6(39.3-71.9)	14.3(1.4-27.2)
**Overall survival**				
Median, months	NE	16.2(13.1-NE)	NE	NE
6-month	92.5(84.3-100.0)	84.4(73.3-98.7)	94.4(87.0-100.0)	89.3(77.9-100.0)
12-month	82.5(70.7-94.3)	68.8(58.9-91.1)	88.9(78.7-99.1)	71.4(54.7-88.1)

Data are n (%) or n (%, 95% CI)

Efficacy and survival are listed in Full analysis set (n=72) and Efficacy analysis set (n=64), respectively. Response was assessed according to the RECIST v1.1. Only confirmed responses were listed.

Abbreviations: CR, complete response; PR, partial response; SD, stable disease; PD, progressive disease; ORR, objective response rate; DCR, disease control rate.

* The patients in each cohort were excluded from the efficacy analysis due to the absence of a post-treatment efficacy assessment

Supplementary Table 4: Cumulative acute adverse events in full analysis set (n=72)

	Any grade	Grade 1-2	Grade 3	Grade 4
**Hematologic**				
Leukopenia	44(61.1)	41 (56.9)	3 (4.2)	0
Neutropenia	32(44.4)	27 (37.5)	5 (6.9)	0
Anemia	15(20.8)	9 (12.5)	6 (8.3)	0
Thrombocytopenia	12(16.7)	8 (11.1)	3 (4.2)	1(1.4)
**Non-hematologic**				
Hypertension,	48(66.7)	28 (38.9)	20 (27.8)	0
Fatigue	37(51.4)	37 (51.4)	0	0
Proteinuria	33(45.8)	32 (44.4)	1 (1.4)	0
Hand and foot syndrome	18(25.0)	9 (12.5)	9(12.5)	0
Loss of appetite	4(5.6)	4 (5.6)	0	0
Reactive cutaneous capillary endothelial proliferation	9(12.5)	7 (9.7)	2 (2.8)	0
ALT increased	24(33.3)	16(22.2)	7 (9.7)	1(1.4)
AST increased	32(44.4)	24(33.3)	8(11.1)	0
Rash	8(11.1)	4 (5.6)	2(2.8)	2(2.8)
Hyperbilirubinaemia	29(40.3)	22 (30.6)	4 (5.6)	3(4.2)
Hypothyroidism	49(68.1)	49(68.1)	0	0
Abdominal pain	3(4.2)	3(4.2)	0	0
Diarrhea	8(11.1)	6 (8.3)	2 (2.8)	0
Nasopharyngeal necrosis	9(12.5)	0	8(11.1)	1(1.4)
Hemorrhage	4(5.6)	2 (2.8)	2 (2.8)	0
Fever	1 (1.4)	1 (1.4)	0	0
Hyperthyroidism	1 (1.4)	1 (1.4)	0	0
Oral mucositis	4(5.6)	4(5.6)	0	0
Pruritus	9(12.5)	9(12.5)	0	0
Myalgia	8(11.1)	8(11.1)	0	0
γ -glutamyl transferase increased	31(43.1)	23(31.9)	8(11.1)	0
Constipation	1 (1.4)	1 (1.4)	0	0
Immune myocarditis	1(1.4)	0	1(1.4)	0
Abdominal pain	3(4.2)	3(4.2)	0	0
Sore Throat	9(12.5)	9(12.5)	0	0
Headaches	3(4.2)	3(4.2)	0	0

Data are n or n (%). Abbreviations: ALT= alanine aminotransferase; AST= aspartate aminotransferase.

Supplementary Table 5: Cumulative acute adverse events in cohort 2 during the neoadjuvant phase (n=32)

	Any grade	Grade 1-2	Grade 3	Grade 4
**Hematologic**				
Leukopenia	12(37.5)	12(37.5)	0	0
Neutropenia	5 (15.6)	4 (12.5)	1 (3.1)	0
Anemia	1(3.1)	0	1 (3.1)	0
**Non-hematologic**				
Hypertension,	6(18.8)	4(12.5)	2 (6.3)	0
Fatigue	5(15.6)	5(15.6)	0	0
Proteinuria	8 (25.0)	8 (25.0)	0	0
Hand and foot syndrome	3 (9.4)	3 (9.4)	0	0
Loss of appetite	1 (3.1)	1 (3.1)	0	0
ALT increase	11(34.4)	6(18.8)	4(12.5)	1(3.1)
AST increase	15(46.9)	11(34.4)	4(12.5)	0
Hyperbilirubinaemia	4 (12.5)	4 (12.5)	0	0
Oral mucositis	1(3.1)	1(3.1)	0	0
Pruritus	1(3.1)	1(3.1)	0	0
Myalgia	1(3.1)	1(3.1)	0	0
γ -glutamyl transferase increased	4(12.5)	3(9.4)	1(3.1)	0
Sore Throat	1(3.1)	1(3.1)	0	0
Headaches	1(3.1)	1(3.1)	0	0

Data are n or n (%). Abbreviations: ALT= alanine aminotransferase; AST= aspartate aminotransferase.

Supplementary Table 6: Biomarker and Subgroup Analysis for Correlation With Clinical Efficacy in All 72 Patients.

Characteristic, n (%)	Cohort 1(n=40)	ORR%(95%CI)	Cohort 2(n=32)	ORR%(95%CI)
Gender				
Male	32(80.0)	71.9(55.4-88.3)	24(75.0)	33.3(13.0-53.7)
Female	8(20.0)	37.5(-5.8-80.8)	8(25.0)	37.5(-5.8-80.8)
Age				
≥ 60	4(10.0)	50.0(-41.9-100.0)	3(9.4)	33.3(-100.0-100.0)
< 60	36(90.0)	66.7(50.5-82.8)	29(90.6)	34.5(16.1-52.9)
Histology				
WHO II	3(7.5)	66.7(-76.8-100.0)	1(3.1)	100.0(NE-NE)
WHO III	37(92.5)	64.9(48.7-81.0)	31(96.9)	32.3(14.8-49.7)
ECOG score				
0	19(47.5)	84.2(66.2-100.0)	15(46.9)	26.7(1.3-52.0)
1	21(52.5)	47.6(24.3-70.9)	17(53.1)	41.2(15.1-67.3)
Liver metastases				
Yes	20(50.0)	70.0(48.0-92.0)	17(53.1)	47.1(20.6-73.5)
No	20(50.0)	60.0(36.5-83.5)	15(46.9)	20.0(-2.9-42.9)
Plasma EBV DNA level(copies/ml)				
≥ 10,000	14(35.0)	50.0(20.0-80.0)	11(34.4)	18.2(-9.0-45.4)
< 10,000	26(65.0)	73.1(54.8-91.3)	21(65.6)	42.9(19.8-65.9)
Previous lines of therapy for advanced disease				
1	24(60.0)	66.7(46.3-87.0)	14(43.8)	21.4(-3.2-46.0)
2+	16(40.0)	62.5(35.9-89.1)	18(56.2)	44.4(19.0-69.9)
Smoking history				
Yes	13(32.5)	69.2(40.2-98.3)	12(37.5)	33.3(2.0-64.6)
No	27(67.5)	63.0(43.5-82.4)	20(62.5)	35.0(12.1-57.9)
Previous radiotherapy				
Yes	24(60.0)	66.7(46.3-87.0)	21(65.6)	33.3(11.3-55.3)
No	16(40.0)	62.5(35.9-89.1)	11(34.4)	36.4(2.5-70.3)
PD-L1 status (TPS)				
Negative	6(15.0)	50.0(-7.5-100.0)	8(25.0)	12.5(-17.1-42.1)
Positive	23(57.5)	65.2(44.2-86.3)	15(46.9)	46.7(18.1-75.3)
Unknown	11(27.5)	72.7(41.3-100.0)	9(28.1)	33.3(-5.1-71.8)
PD-L1 status (CPS)				
Negative	3(7.5)	33.3(-100.-0-100.0)	5(15.6)	20.0(-35.5-75.5)
Positive	26(65.0)	65.4(45.8-85.0)	18(56.3)	38.9(13.9-63.8)
Unknown	11(27.5)	72.7(41.3-100.0)	9(28.1)	33.3(-5.1-71.8)
KDR status				
Negative	8(20.0)	37.5(-5.8-80.8)	8(25.0)	12.5(-17.1-42.1)
Positive	21(52.5)	71.4(50.4-92.5)	15(46.9)	46.7(18.1-75.3)

Unknown	11(27.5)	72.7(41.3-100.0)	9(28.1)	33.3(-5.1-71.8)
PD-LI and KDR status （TPS）				
KDR+PDL1+	19(47.5)	73.7(51.9-95.5)	11(34.4)	54.5(19.5-89.6)
KDR-PDL1+	4(10.0)	25.0(-54.6-100.0)	4(12.5)	25.0(-54.6-100.0)
KDR+PDL1-	2(5.0)	50.0(-100.0-100.0)	4(12.5)	25.0(-54.6-100.0)
KDR-PDL1-	4(10.0)	50.0(-41.0-100.0)	4(12.5)	0(NE-NE)
Unknown	11(27.5)	72.7(41.3-100.0)	9(28.1)	33.3(-5.1-71.8)
PD-LI and KDR status (CPS)				
KDR+PDL1+	20(50.0)	75.0(54.2-95.8)	12(37.5)	50.0(16.8-83.2)
KDR-PDL1+	6(15.0)	33.3(-20.9-87.5)	6(18.8)	16.7(-26.2-59.5)
KDR+PDL1-	1(2.5)	0(NE-NE)	3(9.4)	33.3(-100.0-100.0)
KDR-PDL1-	2(5.0)	50.0(-100.0-100.0)	2(6.3)	0(NE-NE)
Unknown	11(27.5)	72.7(41.3-100.0)	9(28.1)	33.3(-5.1-71.8)
Previous EGFR inhibitor treatment				
Nimotuzumab/ Cetuximab	11(27.5)	72.7(41.3-100.0)	7(21.9)	57.1(7.7-100.0)
No	29(72.5)	62.1(43.3-80.9)	25(78.1)	24.0(6.0-42.0)

Abbreviations: ECOG=Eastern Cooperative Oncology Group; EBV DNA: Epstein-Barr virus (EBV) DNA; EGFR, epidermal growth factor receptor; KDR, vascular endothelial growth factor receptor 2.

Supplementary Table 7: Results of subgroup analysis of PFS and OS in all 72 patients

Characteristic, n (%)	Cohort 1(n=40)		Cohort 2(n=32)	
	PFS	OS	PFS	OS
Gender				
Male	12.6(2.4-22.8)	NE (NE-NE)	24(75.0)	16.2(11.7-NE)
Female	5.1(0.0-17.1)	NE(11.2-NE)	4.5(0.4-8.6)	16.0(6.3-NE)
Age				
≥ 60	4.2(0.0-17.0)	13.4(10.3-NE)	3.4(NE-NE)	11.7(3.9-19.5)
< 60	13.4(3.6-23.3)	NE(NE-NE)	4.6(4.3-4.8)	NE(NE-NE)
Histology				
WHO II	7.1(2.5-11.7)	10.3(5.2-15.3)	4.5(NE-NE)	6.0(NE-NE)
WHO III	13.4(4.5-22.4)	NE(NE-NE)	4.6(3.3-5.8)	16.2(13.1-NE)
ECOG score				
0	NE(NE-NE)	NE(NE-NE)	3.4(0.0-6.9)	NE(NE-NE)
1	5.5(2.0-8.9)	NE(NE-NE)	4.6(4.4-4.8)	15.4(11.5-19.3)
Liver metastases				
Yes	12.6(0.0-28.6)	NE(NE-NE)	3.8(2.7-4.9)	13.5(8.0-19.0)
No	11.7(0.0-25.6)	NE(NE-NE)	6.1(4.1-8.0)	NE(NE-NE)
Plasma EBV DNA level(copies/ml)				
≥ 10,000	5.5(0.0-22.6)	NE(NE-NE)	3.2(2.2-4.3)	11.2(0.0-22.5)
< 10,000	12.6(3.7-21.6)	NE(NE-NE)	4.6(2.3-6.8)	NE(NE-NE)
Previous lines of therapy for advanced disease				
1	7.1(0.0-18.5)	NE(NE-NE)	3.4(1.0-5.8)	NE(NE-NE)
2+	13.4(9.8-17.0)	NE(NE-NE)	4.5(4.3-4.8)	13.5(9.0-18.0)
Smoking history				
Yes	7.1(0.0-18.8)	NE(NE-NE)	3.4(1.7-5.1)	NE(NE-NE)
No	13.4(9.1-17.8)	NE(NE-NE)	4.6(4.5-4.6)	16.0(12.3-19.7)
Previous radiotherapy				
Yes	11.7(3.9-19.5)	NE(NE-NE)	4.5(3.5-5.6)	16.0(6.9-NE)
No	14.3(0.0-31,9)	NE(NE-NE)	6.1(3.2-9.0)	NE(NE-NE)
PD-L1 status(TPS)				
Negative	6.8(0.0-16.1)	NE(NE-NE)	4.4(3.4-5.4)	6.0(0.0-19.5)
Positive	14.3(0.6-27.9)	NE(NE-NE)	3.4(0.0-6.9)	NE(NE-NE)
Unknown	11.7(1.2-22.2)	NE(NE-NE)	4.6(2.6-6.5)	NE(NE-NE)
PD-L1 status(CPS)				
Negative	14.5(0.0-30.8)	NE(NE-NE)	4.5(3.0-6.1)	15.4(0.0-35.7)
Positive	12.6(1.8-23.5)	NE(NE-NE)	3.4(2.0-4.8)	16.2(11.7-NE)
Unknown	11.7(1.2-22.2)	NE(NE-NE)	4.6(2.6-6.5)	NE(NE-NE)
KDR status				

Negative	4.2(0.0-11.5)	13.6(13.1-NE)	3.2(1.8-4.5)	NE(NE-NE)
Positive	NE (7.1-NE)	NE(NE-NE)	4.5(1.6-7.4)	13.5(9.1-17.9)
Unknown	11.7(1.2-22.2)	NE(NE-NE)	4.6(2.6-6.5)	NE(NE-NE)
Previous EGFR inhibitor treatment				
Nimotuzumab/ Cetuximab	7.1(0.0-15.7)	NE(NE-NE)	4.5(1.6-7.4)	NE(NE-NE)
No	13.4(8.9-17.9)	NE(NE-NE)	4.6(3.6-5.5)	NE(NE-NE)

Abbreviations: ECOG=Eastern Cooperative Oncology Group; EBV DNA: Epstein-Barr virus (EBV) DNA; EGFR, epidermal growth factor receptor; KDR, vascular endothelial growth factor receptor 2.

Supplementary Table 8: Characteristics of patients who developed nasopharynx necrosis

Case	Cohort	Locally Recurrence	Previous radiotherapy	Cumulative Apatinib dose (mg)	Grade of necrosis	Surgery of necrosis	Outcome	Reason of death
1	1	No	No	27500	4	Yes*	Survival	/
2	1	No	Yes	87750	3	NO	Survival	/
3	1	No	Yes	148500	3	NO	Survival	/
4	1	No	Yes	145000	3	NO	Survival	/
5	1	No	No	41000	3	NO	Died	NPC
6	2	No	Yes	121500	3	NO	Survival	/
7	2	No	Yes	14000	3	NO	Survival	/
8	2	No	Yes	81000	3	NO	Survival	/
9	2	Yes	Yes	34250	3	NO	Survival	/

* Only one patient (1/72, 1.4%) experienced severe nasal bleeding, but fortunately, no patients died from nasopharyngeal hemorrhage. This was due to the successful embolization of the affected internal carotid artery and implementation of conservative debridement and hyperbaric oxygen therapy, effectively treating all cases of nasopharyngeal necrosis.

Supplementary Table 9 Clinical characteristics of patients with tumor specimens underwent RNA-Seqs analysis

Sample ID	Time Point	MCP-counter	DEGs analysis	Match (Baseline & Post-treatment)	Sampling position	Overall response	response	Cohort	Patient ID
**Sample_01**	Baseline	Yes	Yes	No	cervical lymph node	CR	R	1	1
**Sample_02**	Baseline	Yes	Yes	Yes	nasopharynx	PR	R	1	2
**Sample_03**	Baseline	Yes	No	No	liver	PR	R	1	3
**Sample_04**	Baseline	Yes	Yes	No	cervical lymph node	CR	R	1	4
**Sample_05**	Baseline	Yes	Yes	Yes	nasopharynx	PR	R	1	5
**Sample_06**	Baseline	Yes	Yes	No	lung	PR	R	1	6
**Sample_07**	Baseline	Yes	No	No	liver	PR	R	1	7
**Sample_08**	Baseline	Yes	Yes	Yes	cervical lymph node	PR	R	1	8
**Sample_09**	Baseline	Yes	Yes	No	liver	PR	R	1	9
**Sample_10**	Baseline	Yes	Yes	No	cervical lymph node	PR	R	1	10
**Sample_11**	Baseline	Yes	No	No	thoracic wall skin	PR	R	1	11
**Sample_12**	Baseline	Yes	Yes	No	liver	PR	R	1	12
**Sample_13**	Baseline	Yes	Yes	No	lung	PD	NR	1	13
**Sample_14**	Baseline	Yes	Yes	Yes	cervical lymph node	PD	NR	1	14
**Sample_15**	Baseline	Yes	No	Yes	nasopharynx	PD	NR	1	15
**Sample_16**	Baseline	Yes	Yes	No	liver	PD	NR	1	16
**Sample_17**	Baseline	Yes	Yes	No	cervical lymph node	SD	NR	1	17
**Sample_18**	Relapse	Yes	No	Yes	cervical lymph node	N/A	N/A	1	8
**Sample_19**	Relapse	Yes	No	Yes	cervical lymph node	PR	R	1	2
**Sample_20**	Relapse	Yes	No	Yes	Liver supraclavicular	PD	NR	1	5
**Sample_21**	Relapse	Yes	No	Yes	lymph node	PD	NR	1	15
**Sample_22**	Relapse	Yes	No	Yes	cervical lymph node	PD	NR	1	14
**Sample_23**	Baseline	Yes	Yes	No	nasopharynx	PR	R	2	18
**Sample_24**	Baseline	Yes	Yes	Yes	cervical lymph node	PR	R	2	19
**Sample_25**	Baseline	Yes	Yes	No	cervical lymph node	PR	R	2	20
**Sample_26**	Baseline	Yes	Yes	No	nasopharynx	PR	R	2	21
**Sample_27**	Baseline	Yes	Yes	No	liver	PR	R	2	22
**Sample_28**	Baseline	Yes	Yes	Yes	nasopharynx	PR	R	2	23
**Sample_29**	Baseline	Yes	Yes	Yes	cervical lymph node	PR	R	2	24
**Sample_30**	Baseline	Yes	Yes	No	liver	PR	R	2	25
**Sample_31**	Baseline	Yes	No	Yes	nasopharynx	PR	R	2	26
**Sample_32**	Baseline	Yes	Yes	Yes	cervical lymph node	SD	NR	2	27
**Sample_33**	Baseline	Yes	Yes	Yes	nasopharynx	SD	NR	2	28
**Sample_34**	Baseline	Yes	No	No	lung	SD	NR	2	29
**Sample_35**	Baseline	Yes	Yes	No	liver	PD	NR	2	30
**Sample_36**	Baseline	Yes	Yes	No	cervical lymph node	PD	NR	2	31

**Sample_37**	Baseline	Yes	No	No	liver	PD	NR	2	32
**Sample_38**	Baseline	Yes	Yes	No	liver	PD	NR	2	33
**Sample_39**	Relapse	Yes	No	Yes	parotid gland	PD	NR	2	24
**Sample_40**	Relapse	Yes	No	Yes	cervical lymph node	PR	R	2	26
**Sample_41**	Relapse	Yes	No	Yes	cervical lymph node	PD	NR	2	19
**Sample_42**	Relapse	Yes	No	Yes	cervical lymph node	PD	NR	2	23
**Sample_43**	Relapse	Yes	No	Yes	cervical lymph node	PD	NR	2	27
**Sample_44**	Relapse	Yes	No	Yes	nasopharynx	SD	NR	2	28

44 specimens from 33 patients from different time point (at baseline and post-treatment) were collected in this study. Clinical characteristics patients corresponding to each sample are listed in this table.

Abbreviations: CR, complete response; PR, partial response; SD, stable disease; PD, progressive disease. R, responder; NR; * indicates response defined as achieving a complete or partial response by RECIST 1.1.

Supplementary Table 10. Univariate and multivariate logistic regression analysis predicting response to camrelizumab plus apatinib in cohort 1 using cell population signatures at baseline.

Cell population	Univariate (n=17)			Multivariate (n=17)		
	OR	95% CI	*P* value	OR	95% CI	*P* value
T cells	1.527	1.026 to 2.274	**0.037**	1.073	0.025 to 46.439	0.971
CD8 T cells	1.323	0.991 to 1.765	0.057			
Cytotoxic lymphocytes	1.654	1.004 to 2.725	**0.048**	0.681	0.058 to 7.974	0.760
B lineage	1.382	1.023 to 1.868	**0.035**	1.702	0.335 to 8.641	0.521
NK cells	7.262	0.762 to 69.222	0.085			
Monocytic lineage	1.303	0.984 to 1.725	0.064			
Myeloid dendritic cells	1.515	1.001 to 2.292	**0.049**	0.948	0.224 to 4.020	0.942
Neutrophils	1.677	0.863 to 3.259	0.127			
Endothelial cells	1.661	0.925 to 2.984	0.089			
Fibroblasts	1.161	0.966 to 1.397	0.112			

Regression analyses for MCP-counter output scores for ten cell populations of baseline tumor specimens grouped by response (n = 12 Rs and 5 NRs). Univariate and multivariate analyses as indicated. Abbreviation: CI, confidence interval; NK cells, natural killer cells; OR, odds ratio; P values were calculated by two-sided Wald's test.

Supplementary Table 11. Enrichment scores of gene signatures in baseline tumor specimens in cohort 2.

Patient ID	Sample_28	Sample_25	Sample_24	Sample_29	Sample_23	Sample_26	Sample_30	Sample_27	Sample_33	Sample_32	Sample_36	Sample_35	Sample_38
**Response**	**R**	**R**	**R**	**R**	**R**	**R**	**R**	**R**	**NR**	**NR**	**NR**	**NR**	**NR**
Angio-categlory	High	High	High	High	High	High	High	Low	High	Low	Low	Low	Low
Immune-categlory	Low	High	High	Low	High	Low	Low	High	Low	High	High	Low	Low
Myeloid-categlory	High	Low	High	Low	Low	High	Low	Low	Low	Low	High	Low	Low
Angio	2.566617	0.989393	0.719136	0.595218	0.5185	0.514345	0.273074	0.009543	0.934299	-0.12158	-0.291217	-1.795846	-2.164809
Immune	-4.707703	5.203593	3.885695	-3.583154	3.541065	-0.240777	-1.572815	0.847365	-4.377531	6.452269	1.165538	-0.12304	-4.954901
Myeloid	1.326377	-1.321578	1.344294	-0.724922	-0.856364	1.089153	-1.340523	-1.285319	-1.539338	-0.657236	2.106576	-1.126359	-1.776339

Scores was calculated for each sample (n = 8 Rs and 5 NRs) using z-score method. Gene signatures were defined as follows: angiogenesis (Angio); immune and antigen presentation (Immune); myeloid inflammation (Myeloid). Sample was defined as high/low groups for the corresponding signatures based on cutoff values of mean scores.

Supplementary Table 12. Detailed clinical information of 98 LA-NPC patients for RNA-Seq validation.

Pat_ID	Treatment	Edition of the AJCC Staging System	Plasma EBV DNA level (copies/ml)	PTVnx (Gy)	PTVnd (Gy)
A1	IC+CCRT	4	2500	70	60
A2	IC+CCRT	3	0	70	64
A3	IC+CCRT	5	4010	70	60
A4	IC+CCRT	5	17200	70	66
A5	IMRT alone	3	0	70	64
A6	IC+CCRT	4	28800	70	60
A7	IC+CCRT	4	7420	70	64
A8	CCRT	3	3410	70	60
A9	IC+CCRT	4	200	70	66
A10	IC+CCRT	3	0	70	62
A11	IC+CCRT	3	0	70	60
A12	IC+CCRT	3	865	70	60
A13	IC+CCRT	3	0	70	64
A14	IC+CCRT	4	69000	70	70
A15	CCRT	3	0	70	64
A16	CCRT	2	0	70	54
A17	IC+CCRT	4	26900	70	66
A18	IC+CCRT	4	2500	70	60
A19	CCRT	3	120	70	60
A20	IC+CCRT	4	3470	70	64
A21	IC+CCRT	4	2100	70	66
A22	IC+CCRT	3	12400	70	66
A23	IC+CCRT	4	32100	70	60
A24	IC+CCRT	3	0	70	60
A25	IC+CCRT	3	4670	70	66
A26	IC+CCRT	4	3770	70	66
A27	IC+CCRT	4	5730	70	66
A28	CCRT	3	1580	70	66
A29	IMRT alone	2	0	70	54
A30	CCRT	3	1380	70	64
A31	CCRT	2	0	70	64
A32	CCRT	3	4200	70	66
A33	CCRT	3	553	70	66
A34	CCRT	3	7940	70	66
A35	CCRT	3	3000	70	66
A36	IC+CCRT	4	0	70	66
A37	IC+CCRT	3	15100	70	66
A38	CCRT	3	180000	70	66
A39	CCRT	4	164000	70	66
A40	CCRT	3	0	70	64
A41	CCRT	4	101000	70	62
A42	CCRT	4	0	70	66

A43	IC+CCRT	3	11700	70	64
A44	IC+CCRT	3	751	68	66
A45	IC+CCRT	4	17500	70	66
A46	CCRT	3	43500	70	66
A47	CCRT	3	195	70	64
A48	IC+CCRT	3	37100	70	68
A49	IC+IMRT	4	767	68	64
A50	IC+CCRT	2	11500	70	68
A51	IC+CCRT	5	415000	70	68
A52	IC+CCRT	3	13600	69	64
A53	CCRT	2	0	68	54
A54	IC+CCRT	4	12800	70	68
A55	IC+CCRT	5	26900	70	66
A56	IC+CCRT	3	1220	70	68
A57	CCRT	3	276	70	66
A58	CCRT	5	34400	70	68
A59	CCRT	3	6730	70	68
A60	IC+CCRT	5	43500	70	64
A61	CCRT+AC	5	35300	70	66
A62	CCRT	3	23500	70	68
A63	CCRT	3	7550	70	68
A64	IC+CCRT	3	315000	70	68
A65	CCRT	3	920	70	70
A66	IC+CCRT	4	448	70	70
A67	IC+CCRT	4	5200	70	70
A68	CCRT	3	1020	70	70
A69	IC+CCRT	4	6950	70	70
A70	IC+CCRT	4	428	70	70
A71	IC+CCRT	3	1970	70	70
A72	IC+CCRT	4	445	70	70
A73	IC+CCRT	4	447	70	70
A74	IC+CCRT	4	144	70	70
A75	IC+CCRT	3	2040	70	70
A76	IC+CCRT	4	580	70	70
A77	IC+CCRT	4	755	70	70
A78	IC+CCRT	4	455	70	70
A79	IC+CCRT	4	4630	70	70
A80	IC+CCRT	4	0	70	70
A81	CCRT	4	177	70	68
A82	CCRT+AC	3	310	70	70
A83	CCRT+AC	3	16700	70	70
A84	CCRT+AC	4	217	70	70
A85	CCRT+AC	3	0	70	70
A86	CCRT+AC	4	680	70	70
A87	CCRT+AC	3	243	70	70
A88	CCRT	3	0	70	70
A89	CCRT+AC	3	965	70	70
A90	IMRT alone	1	0	70	70
A91	CCRT+AC	4	815	70	70

A92	CCRT	4	20200	70	70
A93	CCRT+AC	4	758	70	70
A94	CCRT+AC	4	154	70	70
A95	CCRT+AC	3	51.5	70	70
A96	CCRT+AC	3	0	70	70
A97	CCRT+AC	3	223	70	70
A98	CCRT+AC	4	0	70	70

Abbreviations：IMRT Intensity-modulated proton therapy，CCRT Concurrent chemoradiotherapy, IC Induction chemotherapy, AC Ajuvant chemotherapy

Supplementary Table 13. MCP-counter outputs at baseline in 98 LA-NPC patients for RNA-Seq validation.

Pat_ID	T cells	CD8 T cells	Cytotoxic lymphocytes	B lineage	Monocytic lineage	Myeloid dendritic cells	Neutrophils	Endothelial cells	Fibroblasts	B lineage level
A1	4.930055025	4.540190605	5.171122452	5.462001336	6.581232378	4.471150259	5.396409401	3.869729374	6.214768138	high
A2	5.615348899	5.714892028	4.993981075	6.534304648	6.204134648	5.01797237	4.894386291	4.215419466	6.645483412	high
A3	4.121146723	3.715059297	3.948795759	5.381007074	6.035190236	4.484164577	5.330308058	4.889356713	7.985782392	low
A4	4.212014814	3.682928272	3.822493007	2.984076635	4.769748045	4.38269833	4.816910811	3.92300509	6.57272158	low
A5	4.853886125	3.235254071	4.292034996	5.83538265	6.113051419	4.606085281	4.953298362	4.036580859	6.326601963	high
A6	5.12344786	5.442643685	4.877078584	6.857553035	5.779068506	3.953764875	5.380689211	4.540349311	7.288728534	high
A7	4.423956201	3.788811145	4.551951922	4.622909679	6.034562554	3.948116237	5.208855825	4.217186553	7.192795223	high
A8	4.939376203	4.014538844	5.29147551	6.112461072	6.38117549	5.005554008	5.30875956	3.487981338	6.434376566	high
A9	5.224908478	4.45968154	5.64328704	5.037739645	6.476248685	4.268166064	5.100959712	3.719683159	7.367633481	high
A10	4.418232617	3.505231424	4.610827261	5.375494104	5.583155267	4.837989064	4.802527798	3.332057936	6.091948559	high
A11	5.106721342	5.000193324	5.847967203	4.995642884	6.452138059	3.419240861	4.936609656	5.256309541	8.410551044	high
A12	5.239287065	4.57961396	4.614347952	6.786702136	6.140045046	5.135563604	4.815312441	3.969254916	5.701404502	high
A13	5.029292889	4.491041172	5.352105622	5.059550515	6.961721302	4.851259206	4.42149464	3.731833578	6.885351497	high
A14	5.207837785	4.553055537	4.129613482	6.881112957	5.817175843	4.454778378	4.867300679	4.220349336	6.010006089	high
A15	3.869716214	3.597779379	4.782622068	3.615891509	5.688650203	3.773457035	5.138335982	3.968102767	7.488262249	low
A16	4.758518791	4.454083771	5.166696754	5.28493637	6.362136979	4.136130692	4.959763029	3.596521163	6.52906878	high
A17	4.4354846	3.844289881	4.679537897	4.670696722	6.738452299	3.012462538	5.566014172	3.341581492	7.281448211	high
A18	5.67395536	4.169405412	5.197914716	7.283240254	7.033991597	4.204217388	4.87846186	4.963215994	6.884351558	high
A19	4.959916805	4.658330201	5.018025352	7.662155767	6.325353749	4.644467279	4.894350034	3.82331835	6.615294774	high
A20	5.176643667	3.974466233	4.739630413	7.770444015	6.144490125	4.417722438	4.993066489	4.046885103	6.496718784	high
A21	4.55439663	3.436178714	4.2478799	4.657608171	5.900706821	5.137934338	5.020772707	3.930900942	6.436916534	low
A22	4.882631768	5.008474611	5.600626179	3.32373564	6.023629052	5.033095922	4.642702399	3.656297523	6.068513037	high
A23	5.773447103	6.034069892	5.556820298	5.851167386	6.655490006	4.041122799	5.211352889	3.305239546	5.735658634	high
A24	5.359110432	4.552168075	5.347222865	6.978486816	5.984581213	5.256559517	4.875763102	3.88793955	5.996280675	high

A25	3.149088128	2.794235444	3.55900796	4.028185476	5.673385192	3.643711917	4.238734951	3.337684023	5.793967549	low
A26	4.384253514	1.998207866	3.740443504	6.23090535	6.795862736	3.585775553	5.460057086	5.124547822	7.563083472	low
A27	4.802483371	4.447577083	4.788460513	3.53241313	5.505576497	3.939830134	4.214021208	3.272221115	6.395218603	low
A28	5.761676282	5.648938757	6.19293746	5.670639021	6.631418726	4.422047933	5.51248896	4.040203991	6.926856767	high
A29	3.990560984	3.038775073	4.427133103	4.828411659	5.449712102	4.811981745	4.956877046	3.760533588	5.31470573	low
A30	5.760141324	5.109264267	6.049348495	7.131073369	6.991682489	5.144985811	5.114008644	4.234110025	6.20334257	high
A31	4.553449628	3.050031805	4.463012506	3.540419789	5.007367977	4.370254691	4.766717633	3.670212841	6.907300736	low
A32	2.865288744	2.110090801	3.248960732	3.152738307	5.269713268	2.304433903	4.215397017	2.925439187	6.336514474	low
A33	4.222214829	2.901750481	4.697445585	5.179482547	6.217342946	4.317236428	4.648911419	4.066833159	7.880164755	low
A34	4.082394044	4.471697829	5.189169969	2.811957487	5.874951423	3.64488573	4.909273366	4.483768205	5.471990073	low
A35	4.36883734	4.021448293	4.606421689	4.772570664	5.513539555	5.012011668	5.078319142	3.662315327	6.647410946	low
A36	4.011317741	3.686443398	4.156618689	2.559968445	5.671679129	5.061764905	5.66698191	3.850831557	6.442221665	low
A37	3.808614036	3.434429243	3.643780593	3.358486519	5.270823418	5.586150221	4.161308481	3.631022608	6.656844804	low
A38	4.429857086	3.787491865	4.34910035	3.800582884	6.240637062	4.545102579	4.311855526	3.017507127	5.950527056	low
A39	4.444531048	4.345391885	5.11764319	4.537978576	6.292250213	3.642132865	4.406682249	3.001749447	5.441392082	low
A40	5.340475684	5.487440583	4.976793277	6.331175651	6.020694461	4.828997868	5.313042868	4.515747291	7.881622009	high
A41	3.443852764	2.972296309	3.575702305	3.069051184	4.804992018	3.128229056	4.907478352	3.840306342	5.968745782	low
A42	2.606219807	1.081858631	1.912501097	0.594433992	4.720686369	2.992376744	4.298426782	3.953816637	8.251311625	low
A43	4.979479118	3.709712205	4.604454943	6.929835451	5.915412336	5.018386267	4.900395684	4.24946748	6.374329585	high
A44	5.080285695	5.510179899	5.759432387	4.009811317	6.63685996	5.470465804	4.479847927	3.615344882	6.187691376	high
A45	3.206787873	3.592146027	4.407984034	2.415836092	4.676722765	3.58382544	4.401787955	3.473724282	8.066337228	low
A46	3.179679307	2.829372681	3.118462897	2.597068492	5.075442446	2.892883932	3.985687404	2.964738081	4.10453727	low
A47	5.054358409	4.626566413	5.585896499	6.073531782	6.058707649	5.513387994	4.680195093	3.800036181	6.673903617	high
A48	2.873505522	3.147562354	4.929591768	2.710895204	6.489505774	3.919978092	4.611250949	4.106388604	5.850861915	low
A49	4.596603658	3.89962049	4.782144105	4.547982529	6.599264669	5.113476733	5.498668183	4.420414447	7.14483678	low
A50	4.227425177	3.588738551	3.976622408	3.575780523	6.019482348	3.745350502	7.024254033	4.413399165	8.02734741	low
A51	3.756065813	3.801779079	4.265837319	3.73676648	4.796735391	3.372209328	4.438648544	3.722903405	6.322390239	low
A52	3.934368607	3.544694132	4.563451916	4.007152879	5.390359915	3.921971186	4.082990175	3.847840017	5.872384333	low
A53	5.688909671	5.425994769	6.022366478	7.026854289	6.534964345	4.346878775	4.409539677	3.559789857	6.295872217	high

A54	4.565930286	3.706611679	4.558575336	3.761723895	5.578099701	4.362383818	4.418382569	3.320825413	5.949761078	low
A55	4.034557438	2.450286354	3.682926525	3.956361826	7.055054526	3.94548237	4.205479422	4.005604656	7.000856775	low
A56	3.430335465	1.805182905	2.192456277	2.660745178	5.289919704	3.80585018	4.429221848	3.111424597	6.780410565	low
A57	4.597733094	4.44836958	4.297861121	3.39658641	6.324955399	4.322552889	4.506877042	3.042566318	4.56782851	low
A58	3.847949492	1.974296078	2.488116294	5.60533268	4.564598777	2.631153015	5.084408054	3.44778861	6.017303338	low
A59	4.440106348	4.621025537	5.29074373	5.334666721	6.758584115	3.199687049	6.358544209	3.628424206	6.357368686	high
A60	5.217409463	4.675995554	5.665231819	7.594882099	6.651382267	5.042965348	4.85402355	4.067241669	6.954714029	high
A61	4.565255167	4.088263571	4.895263833	5.181153286	6.116286742	4.213899272	4.79869886	3.62902968	6.484452426	high
A62	2.401150092	1.675144676	2.54393155	1.598539971	5.061821461	2.169760509	3.948955894	2.97632677	5.094860562	low
A63	2.647631085	2.172935784	2.434662402	2.950719929	5.317116353	2.823957852	4.26349369	2.881129593	6.120955928	low
A64	5.261069303	5.435114459	5.760662799	4.512625713	6.277568681	4.086812165	5.096472316	3.564778255	6.007520418	high
A65	4.931562956	3.975992734	4.620797208	5.746826746	6.138984136	4.418842412	4.752556576	3.229262849	5.64074397	high
A66	5.566004389	4.824088879	5.085891905	7.573432152	6.605229116	4.826674377	5.32038251	4.606822349	7.583839116	high
A67	4.074648614	2.965685316	3.647231561	4.059249121	5.43839541	3.170734243	4.256709863	2.894130218	4.848279659	low
A68	4.801940518	4.784901069	4.013525247	7.246202135	5.955753167	4.716076319	5.095528612	3.972320789	6.124389987	high
A69	4.196053787	3.043858665	4.17116459	4.90354554	6.615120571	4.151594304	5.221703438	3.697161107	6.776251447	low
A70	4.061352512	3.105703192	3.888541884	3.767469101	5.973700045	5.062967939	4.634948152	4.331627247	6.772496726	low
A71	4.056886612	2.997554039	3.377682869	5.189357105	5.843184827	3.681764995	5.447443823	3.639544428	6.287689642	low
A72	5.158955532	5.285338515	4.66216345	6.407642276	5.571715613	4.910871737	4.425696124	3.444488044	6.697967696	high
A73	5.365665542	4.099145738	4.682627206	7.31684001	6.07523338	4.720081006	5.168411418	4.412936332	6.3624623	high
A74	3.238750281	3.262020592	4.405549185	3.245666864	5.304711233	3.117165326	4.304265367	3.828801155	7.232881517	low
A75	4.341247032	4.15197996	4.805786748	4.847160741	6.844664295	3.607536561	4.631293828	3.989898744	7.199418901	high
A76	3.508907072	2.494719328	3.442614785	2.688433241	5.289829762	4.402434783	4.764673594	3.577651251	6.264337187	low
A77	4.19477231	3.646634393	4.464610247	3.556653519	5.703300927	4.375071887	5.231396539	3.515570575	5.29197078	low
A78	3.583039419	3.65629791	4.444056442	2.348893315	4.940480746	3.076688558	4.793196783	3.401319339	6.50479098	low
A79	5.151224267	5.61815097	6.461104114	4.93362107	7.021476317	4.197637038	4.855833416	4.463667036	7.207946247	high
A80	4.959441858	4.465731948	5.221385741	4.955043557	5.73661245	5.219799626	4.785290409	3.392270414	6.07303513	high
A81	2.710372683	3.015121465	4.191910384	1.378527422	4.538876748	2.297709934	3.402145734	2.765129414	5.226666085	low
A82	4.089901074	2.459892669	4.057232727	4.466245641	5.879527309	4.566269273	5.838495749	4.161921922	6.910372149	low

A83	3.665382461	2.581203155	3.750560045	4.748570222	5.71868096	4.79699473	4.639548129	3.035985261	6.101297886	low
A84	5.131569936	5.678038861	5.750294085	5.575388116	6.256828543	3.819410245	4.787314178	2.72210452	5.038460984	high
A85	3.392426472	3.071621018	3.519458387	3.666287477	4.297953373	2.158654157	5.043809768	3.521957669	6.181115685	low
A86	3.856127948	3.890156776	4.605492553	4.908792776	6.114417775	3.31485188	5.069930818	3.553568499	6.463678685	high
A87	5.501782977	5.322751303	4.197264957	6.432608136	6.610059283	4.430369883	5.687175251	4.50392691	5.759837465	high
A88	2.973423056	1.833902015	3.237897043	2.832557413	4.835110097	2.751209895	4.50811771	3.257601171	5.7526428	low
A89	4.081749761	3.032350929	4.168415806	3.088407979	5.071792734	4.674433671	5.040671234	4.411053616	7.915489005	low
A90	5.540382142	4.768758372	4.456102933	7.239441745	5.949351427	5.240766007	4.887035787	4.264097994	6.28193212	high
A91	5.055948913	4.122889173	4.502798957	6.597091146	6.229884677	5.029084933	5.098640019	4.614221548	7.371139026	high
A92	5.587536348	4.550208136	4.566522643	6.288720375	5.903536904	4.884815654	5.073917867	4.641136335	6.563656953	high
A93	4.2561156	3.883137271	3.904042257	4.438327562	5.917091342	5.583332705	4.04735573	3.276102953	5.682922695	low
A94	4.603891231	4.456116286	5.280971601	1.893770201	6.736713308	4.72842061	4.000443615	3.667113823	7.676540772	low
A95	5.206940537	4.608757444	5.307578986	7.081212267	6.631656455	3.636087281	4.808605539	3.690496984	6.119200657	high
A96	3.58974262	3.862238514	4.915792964	2.589473133	6.062404978	2.597644101	4.229185145	3.239577063	6.846180643	low
A97	5.309056271	5.106096453	5.820127625	6.989918704	6.40207872	4.491888012	4.9503001	4.205508125	6.294265211	high
A98	4.882845414	4.376774606	6.331140411	3.85156054	6.897706053	4.380286589	4.498192087	4.124480959	7.122415939	high

MCP-counter output scores for ten cell populations (eight immune cell types, endothelial cells and fibroblasts) of tumor specimens from 98 LA-NPC patients are showed in this table. Unsupervised hierarchical clustering analysis were applied to divide this validation cohort into B lineage score high group and low group. The corresponding grouping of each sample patient is summarized in the "B lineage level" column.

Supplementary Table 14. Summary of clinical endpoints of included trials.

	Nivolumab	Pembrolizumab	Camrelizumab	Toripalimab	Apatinib	Apatinib+Camrelizumab
**PFS**						
**Median(months)**	2.8(1.8-7.4)	3.7(2.1-13.4)	5.6(3.3-7.9)	1.9(1.8-3.5)	3.9(3.1-5.5)	12.6(1.5-23.7)
**1-year rate (%)**	19.3	33.4	27.1	17.4	NR	52.5
**OS**						
**Median(months)**	17.1(10.9-NE)	16.5(10.1-NE)	NR	17.4(11.7-22.9)	5.8(4.5-8.0)	NE(NE-NE)
**1-year rate (%)**	59	63	NR	NR	34.3	82.5

Comparison of Anti-PD-1 Monotherapy (Pembrolizumab, Nivolumab, Toripalimab, Camrelizumab), Single-Agent Apatinib, and our Combination Therapy (Cohort 1) in RM-NPC. We have listed the median PFS, 1-year median PFS (months), 1-year PFS rate (%), median OS (months), and 1-year OS rate (%) for each study. Abbreviations: NR not reported, OS overall survival, PFS progression-free survival, NE not reached

Supplementary Table 15. Gene signatures representing potentially clinically relevant pathways from RNA sequencing data in cohort 2.

Gene signature	Gene content
Angiogenesis[1]	*ANGPT4, CD34, ESM1, FLT1, KDR, PECAM1, VEGFA*
Immune and antigen presentation[2]	*CD27, CD274, CD8A, CTLA4, CXCL10, CXCL11, CXCL9, EOMES, FOXP3, GZMA, GZMB, IDO1, IFNG, PRF1, PSMB8, PSMB9, TAP1, TAP2, TIGIT*
Myeloid inflammation[3-6]	*CXCL1, CXCL2, CXCL3, CXCL8, IL6, PTGS2*

Three gene expression signatures (angiogenesis, immune and antigen presentation and myeloid inflammation) were defined based on their respective biology (gene content) from previously published studies.

Supplementary figure 1

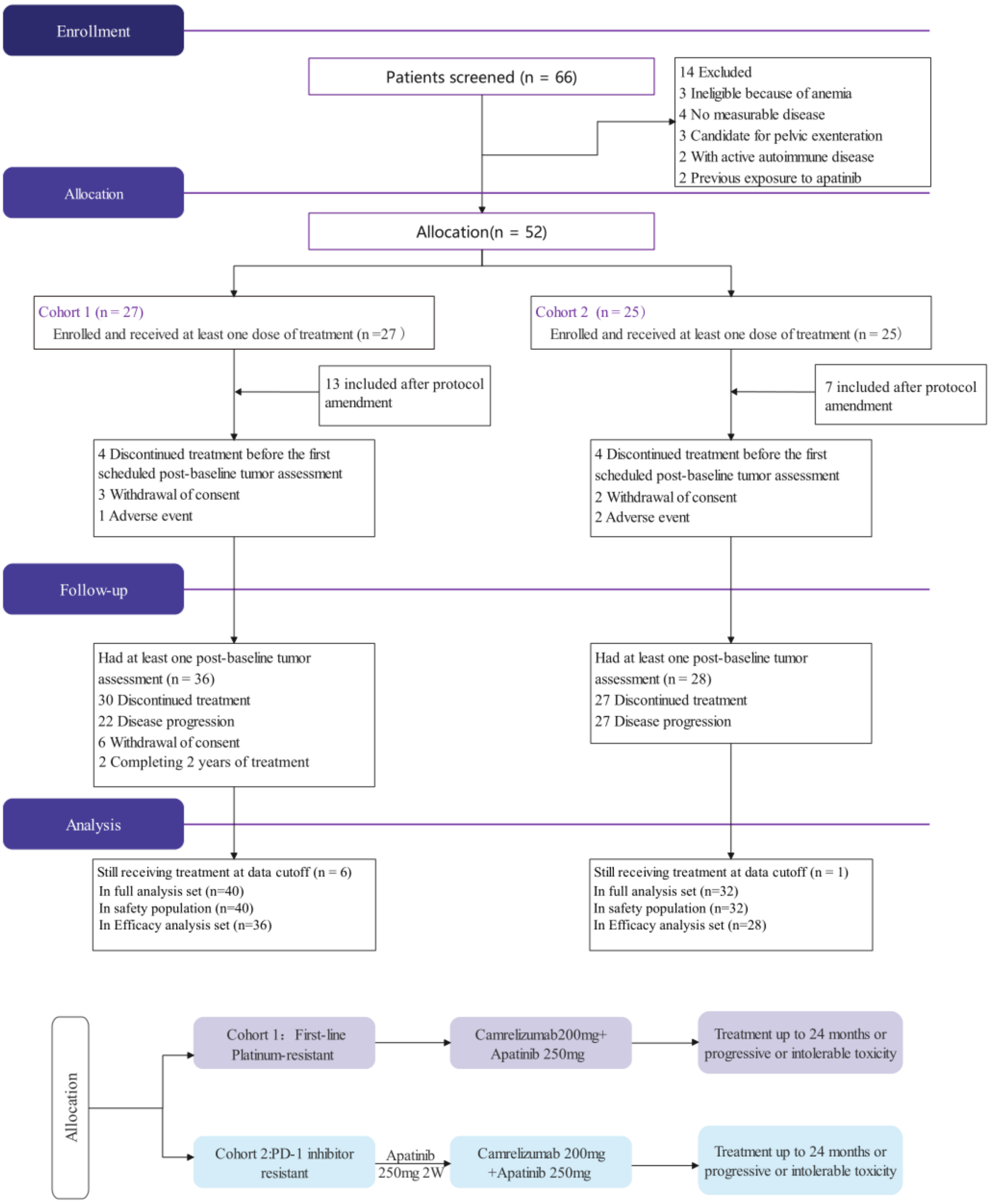

Supplementary Figure. 1. Flow chart of the study.

This Fig. illustrates the study design.

Supplementary figure 2

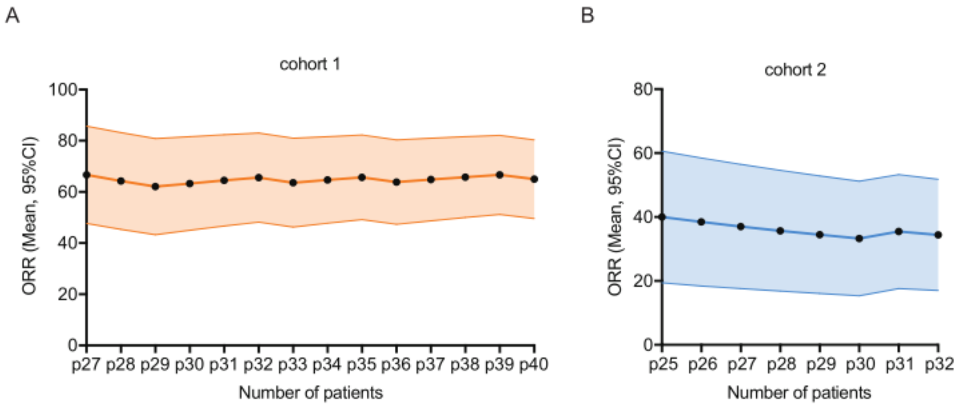

Supplementary Figure 2. The stability of objective response rate (ORR) as the sample size increased.

ORR and 95% confidence intervals of cohort1 (A, n=14) and cohort 2 (B, n=8).

Supplementary figure 3

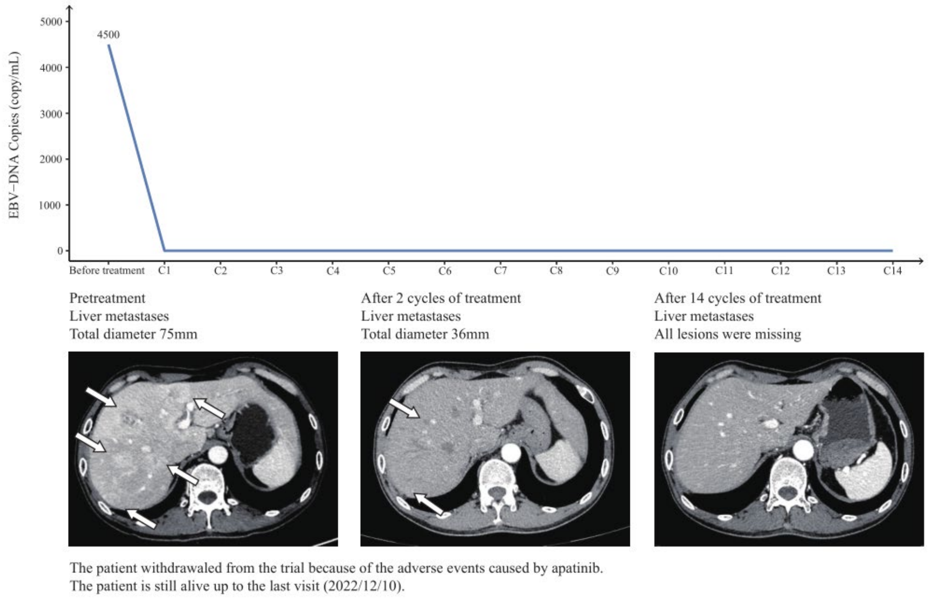

Supplementary Figure 3. Radiological images of a representative patient in cohort 1.

The upper part shows the plasma EBV DNA level of the representative patient at baseline and every time before camrelizumab plus apatinib cycle started.

The lower part shows the CT scanning of liver metastases at baseline, 2 cycles after treatment, and 14 after treatment.

Supplementary figure 4

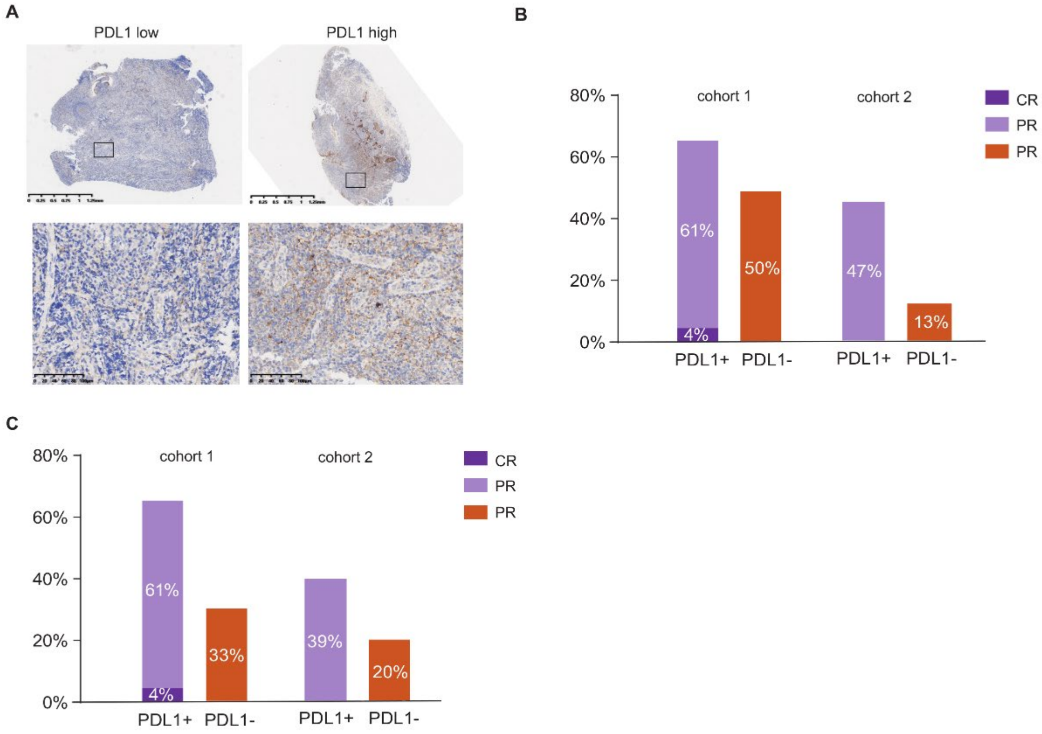

Supplementary Figure 4. IHC of PD-L1 expression.

A. Representative IHC images of negative (left) and positive (right) expression of PDL1. Scale bar is 1.25 mm or 100 μm. **B- C.** Quantification of PD-L1 expression (B. CPS of PD-L1 and C. TPS of PD-L1) by immunohistochemistry and association with response to camrelizumab combined with apatinib in patients with first-line platinum-resistant (cohort 1, total n = 40, CR n=3, PR=23) or PD-1 inhibitor resistant (cohort 2, total n = 32, PR n=11) recurrent/metastatic nasopharyngeal carcinoma. Source data are provided as a Source Data file.

Supplementary figure 5

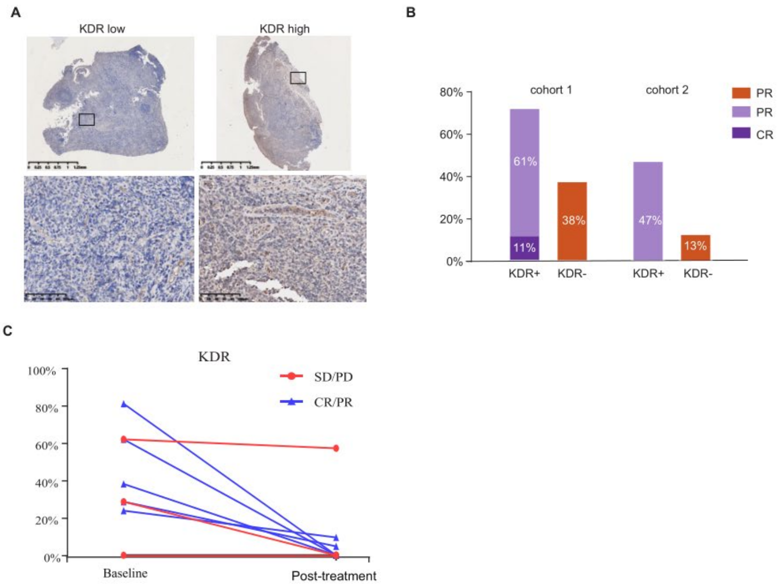

Supplementary Figure 5. KDR expression in different cohort.

A. Representative IHC images of negative (left) and positive (right) expression of KDR. Scale bar is 1.25 mm or 100 µm. **B.** Quantification of KDR expression by immunohistochemistry and association with response to camrelizumab combined with apatinib in patients with first-line platinum-resistant (cohort 1, total n = 40, CR n=3, PR=23) or PD-1 inhibitor resistant (cohort 2, total n = 32, PR n=11) recurrent/metastatic nasopharyngeal carcinoma. **C.** Quantification of KDR expression before and after treatment and between responses (n = 9 baseline and 9 post-treatment). Source data are provided as a Source Data file.

Supplementary figure 6

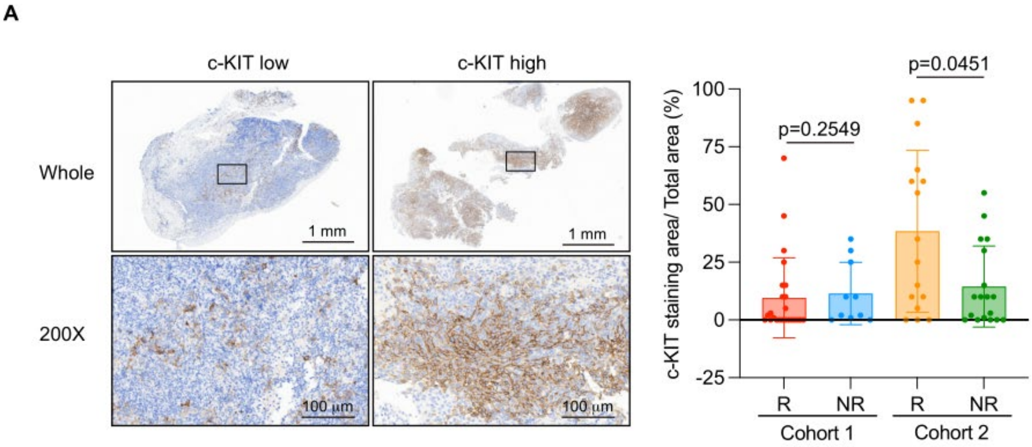

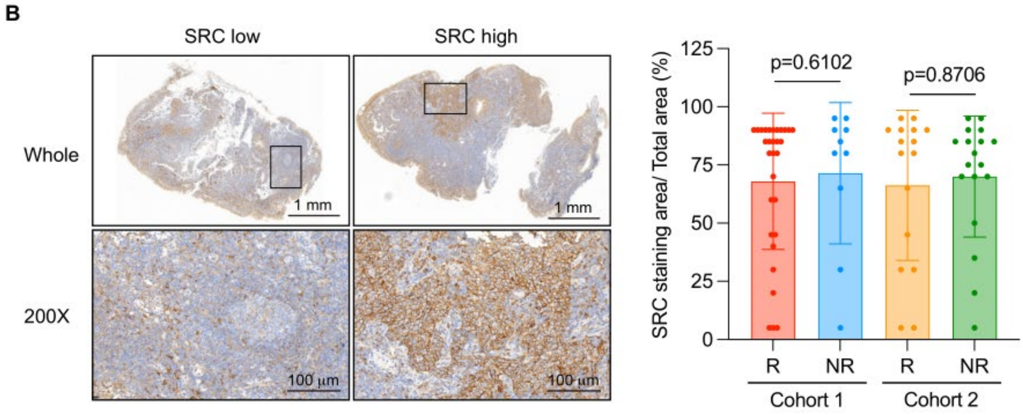

Supplementary Figure 6. C-KIT and SRC expression.

A-B. Representative image of c-KIT (A) and SRC (B) immunohistochemistry staining in patients (left, Scale bar is 1 mm or 100 μm.) and quantification of c-KIT or SRC density (right) by immunohistochemistry and association with response to camrelizumab combined with apatinib in patients with first-line platinum-resistant (cohort 1, n = 10 NRs and 24 Rs) or PD-1 inhibitor resistant (cohort 2, n = 18 NRs and n=16 Rs) recurrent/metastatic nasopharyngeal carcinoma. Data were presented as mean values ± SD, each dot represented one sample. *P* values were calculated by two-tailed, Mann-Whitney U-test. Source data are provided as a Source Data file.

Supplementary figure 7

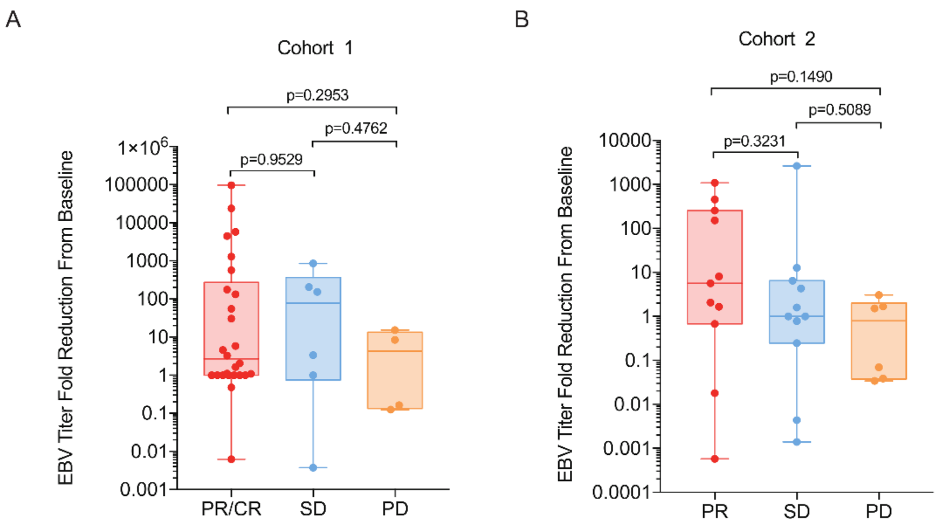

Supplementary Figure 7. Plasma EBV DNA titer fold reduction from baseline.

Plasma EBV DNA copy numbers of cohorts 1 (A, n=40) and 2 (B, n=32) were assessed at baseline and on day 28. In patients with objective response, stable disease, or progressive disease, the fold decrease of EBV titer from baseline to day 28 was shown. All data in box and whiskers plots are represented as median value, the first and third quartile, maximum and minimum. *P* values were calculated by two-tailed, Mann-Whitney U-test. Source data are provided as a Source Data file.

*Fold reduction = (baseline EBV tier)/ (day 28 EBV titer). CR, complete response; EBV, Epstein-Barr virus; PR, partial response.

Supplementary figure 8

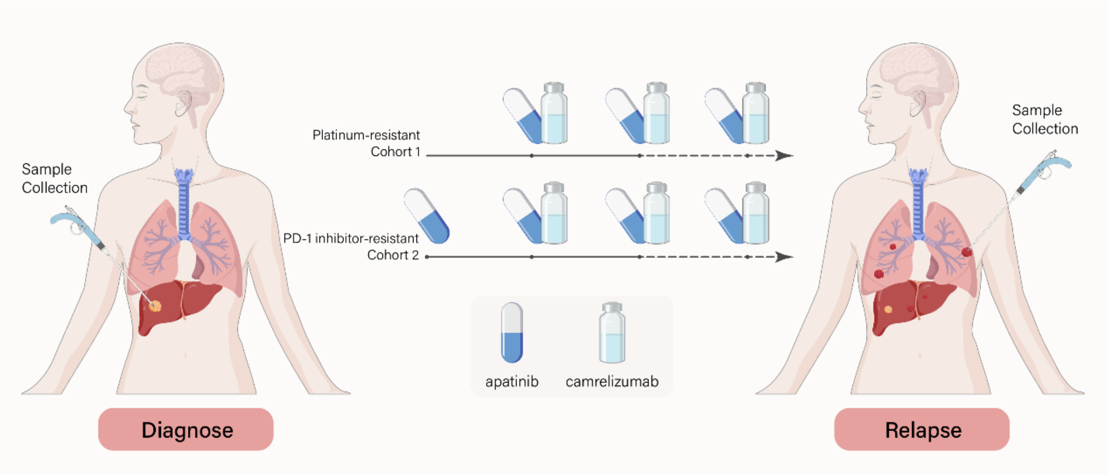

Supplementary Figure 8. Schematic diagram of longitudinal tumor samples.

Supplementary figure 9

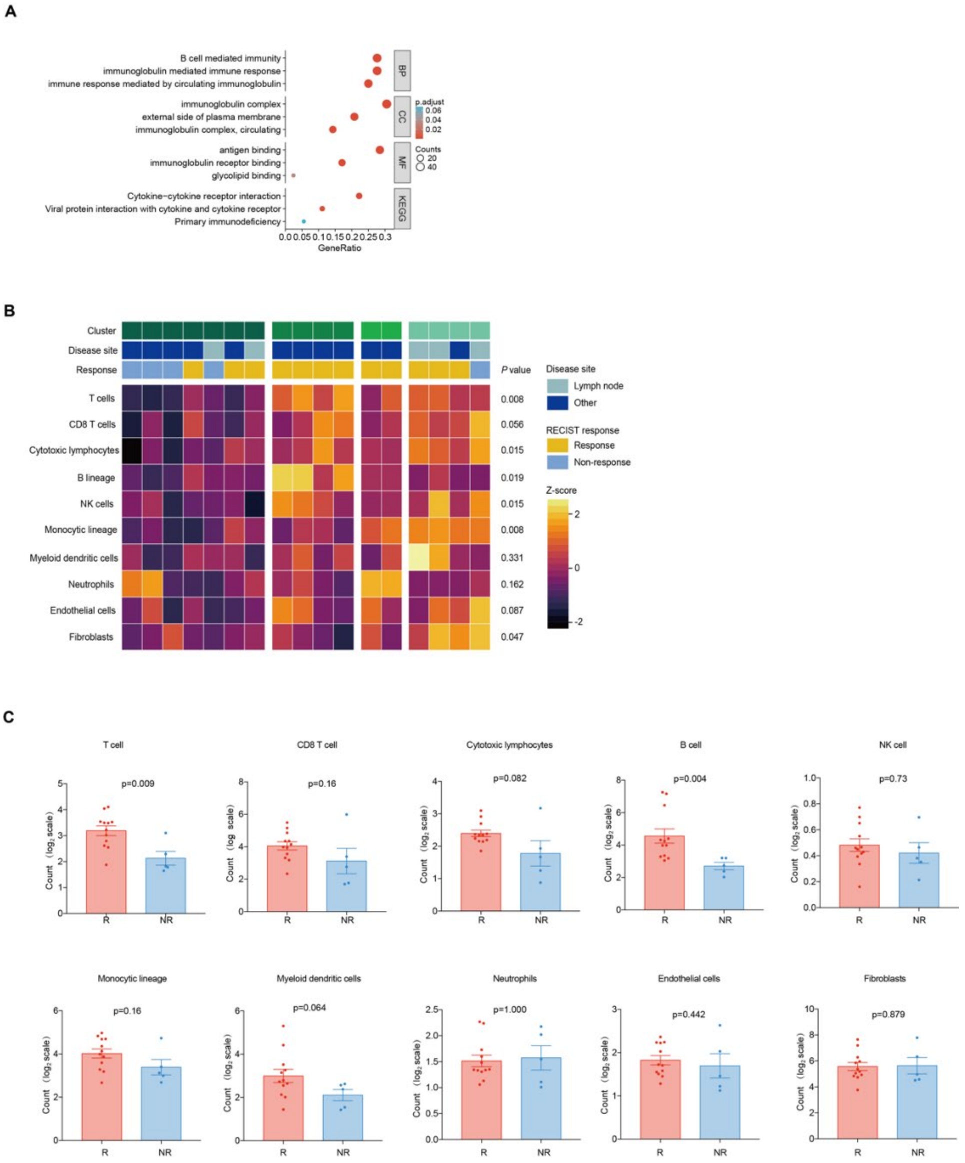

Supplementary Figure 9. Gene expression characteristics and enrichment in cohort 1.

A. KEGG enrichment analysis of differentially expressed genes from baseline tumour specimens by response (n = 4 non- responders and 9 responders). **B.** Unsupervised hierarchical clustering analysis shown for baseline tumour specimens by response (n = 12 Rs and 5 NRs). Unique clusters are indicated by green color on top row. *P* values were calculated by two-sided Mann-Whitney U-test. **C.** Box plot representation of heat maps for patients as presented in Fig. 2C (n = 12 Rs and 5 NRs). Medians with interquartile range are shown. *P* values were determined by two-sided Mann–Whitney U-test.

Supplementary figure 10

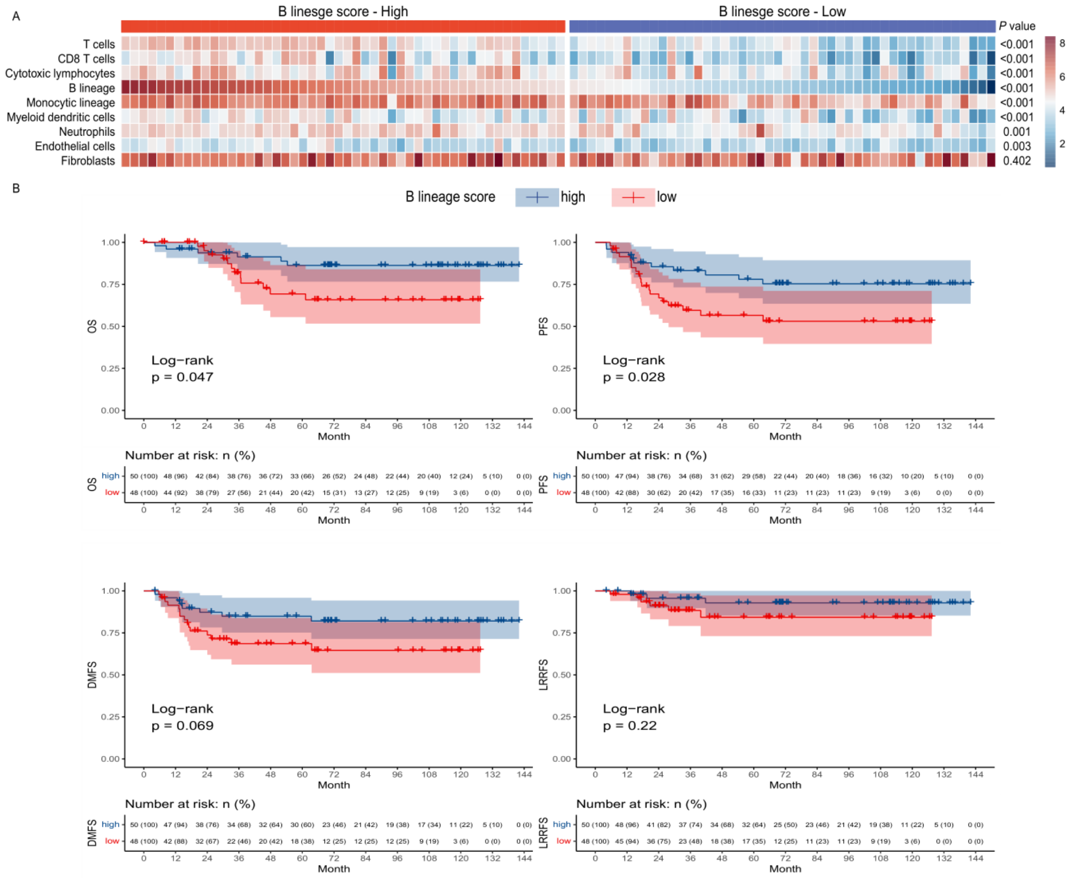

Supplementary Figure 10. B cell is associated with prognosis of patients with nasopharyngeal carcinoma

A. Supervised clustering of MCP-counter scores in a bulk RNA-Seq cohort of LA-NPC patients. According to the mean value of B lineage score (4.767), patients were divided into B lineage score- high (n=50) and low (n=48) group. *P* values were calculated by two-sided Mann-Whitney U-test. **B.** Kaplan–Meier curves showing the survival rate of LA-NPC validation cohort in different group (B lineage-score high n=50 and low n=48). *P* values were calculated by Log-rank test.

Supplementary figure 11

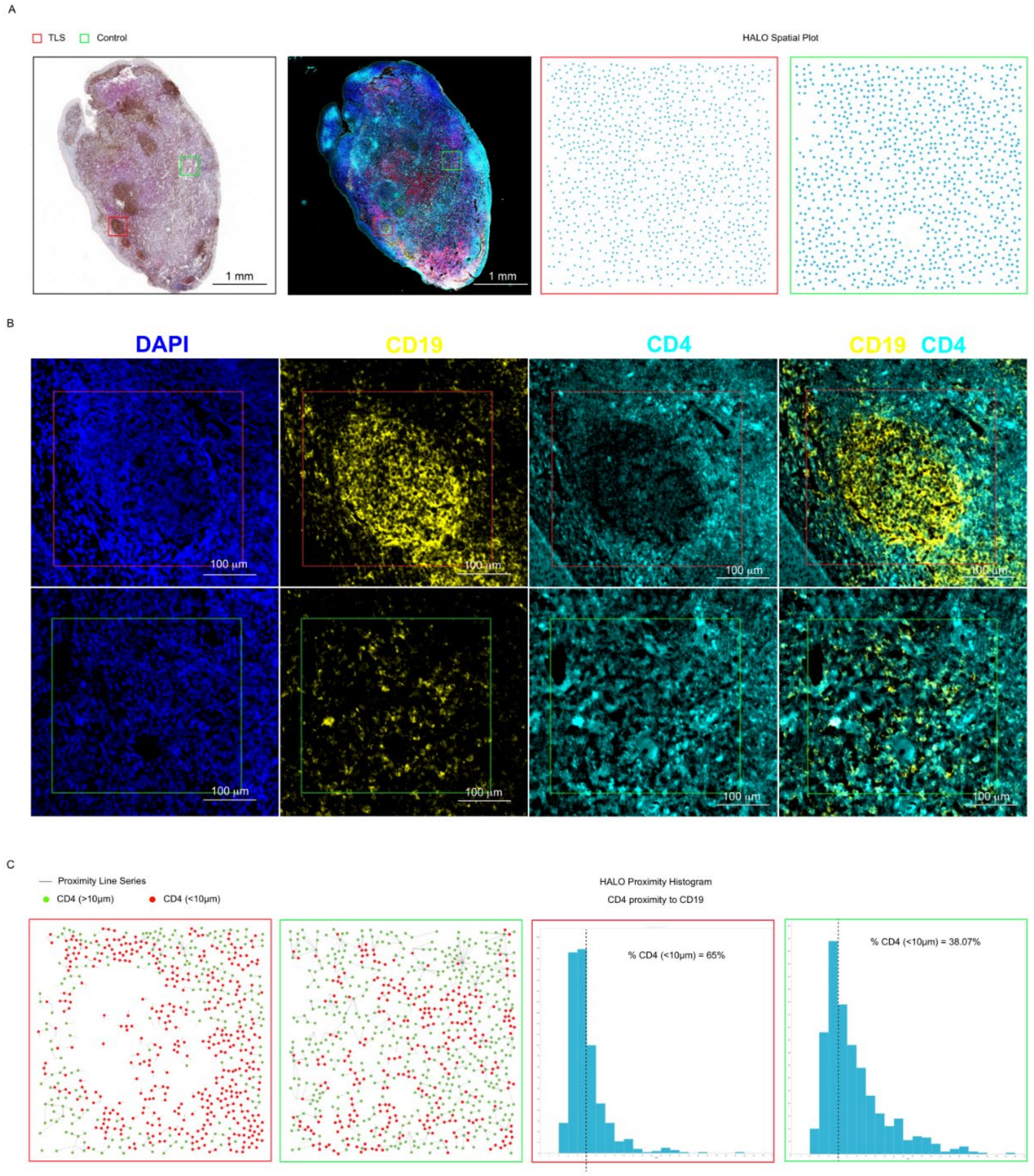

Supplementary Figure 11. Tertiary lymphatic structure and spatial distance analysis.

A. IHC and mIHC image of Tertiary lymphatic structures (the red box) and control (the green box) from the same patient. The plot maps on the right show the spatial location of each cell identified by the halo analysis software. Scale bar is 1 mm. **B.** Representative images of multiplex immunofluorescence staining of TLS and control

in NPC tissues. Proteins detected using respective antibodies in the assays are indicated on top. The red, blue, and yellow plots indicated the representative cells positive for CD8, CD4, and CD19, respectively. Magnification: 200x. Scale bar is 100 μm. **C.** Taking the CD19 positive cells as the center, the CD4 cells with distances less than 10mm (red plots) and greater than 10mm (green plots) were displayed, and the numbers were counted according to the distance.

Supplementary figure 12

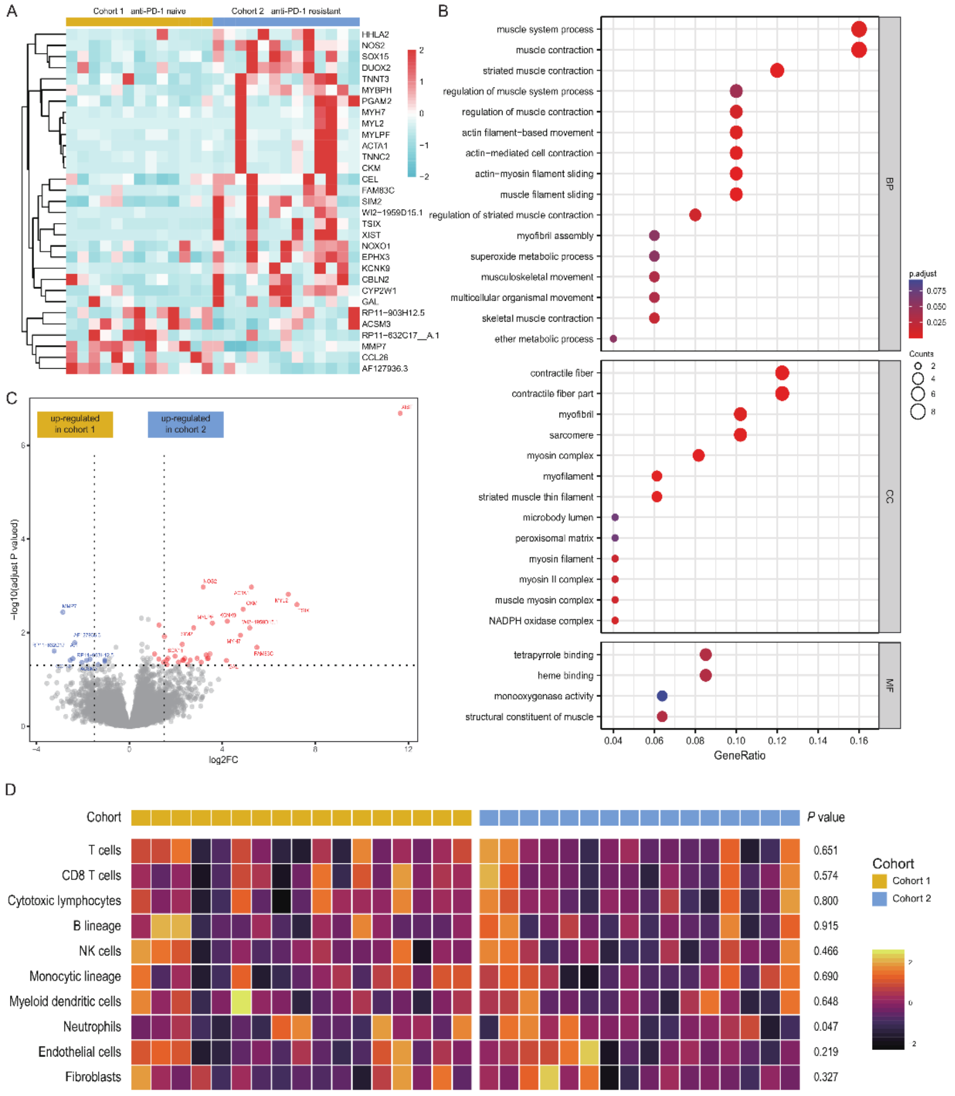

Supplementary Figure 12. Transcriptional analysis of tumor specimens from patients in different cohorts at baseline.

A. Supervised hierarchical clustering of differentially expressed genes (DEGs) on RNA-seq analysis of tumor specimens from cohort 1 (N=13) and cohort 2 (N=13) at baseline. DEGs were selected based on following criteria: log_2-transformed FC >2 or <-2 and a DESeq2-adjusted *P* value of <0.05. **B.** Volcano plot depiction of DEGs from different cohorts. **C.** GO analysis were applied in DEGs with DESeq2-adjusted *P* value of <0.05. **D.** Supervised clustering by cohort of MCP-counter scores for z-score normalization in baseline tumor specimens (n = 12 Rs and 5 NRs). NK cells, natural killer cells. *P* values were calculated by two-sided Mann-Whitney U-test.

Supplementary figure 13

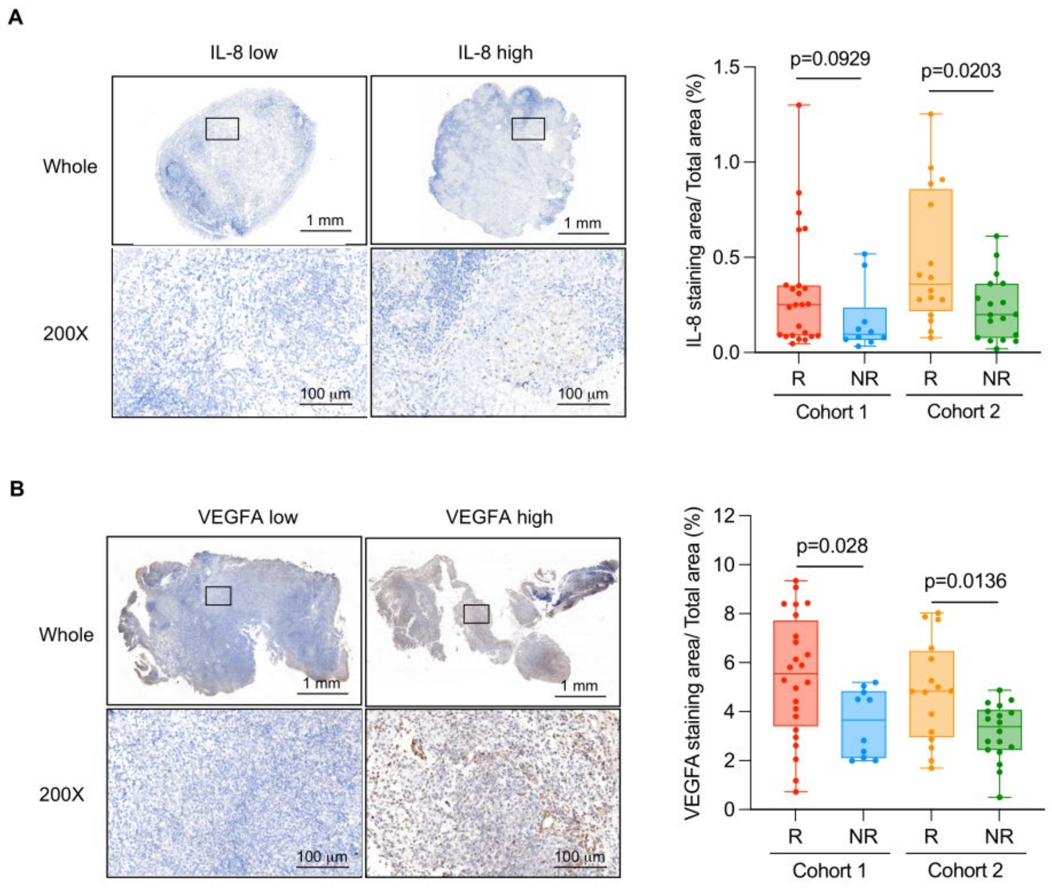

Supplementary Figure 13. Blood vessel markers (VEGFA and IL8) expression.

A-B. Representative image of IL-8 (A) and VEGFA (B) immunohistochemistry staining in patients (left, Scale bar is 1 mm or 100 µm.) and quantification of IL-8 or VEGFA density (right) by immunohistochemistry and association with response to camrelizumab combined with apatinib in patients with first-line platinum-resistant (cohort 1, n = 10 NRs and 24 Rs) or PD-1 inhibitor resistant (cohort 2, n = 18 NRs and n=16 Rs) recurrent/metastatic nasopharyngeal carcinoma. All data in box and whiskers plots are represented as median value, quartile, maximum and minimum; each dot represented one sample. *P* values were calculated by two-tailed, Mann-Whitney U-test. Source data are provided as a Source Data file.

Supplementary figure 14

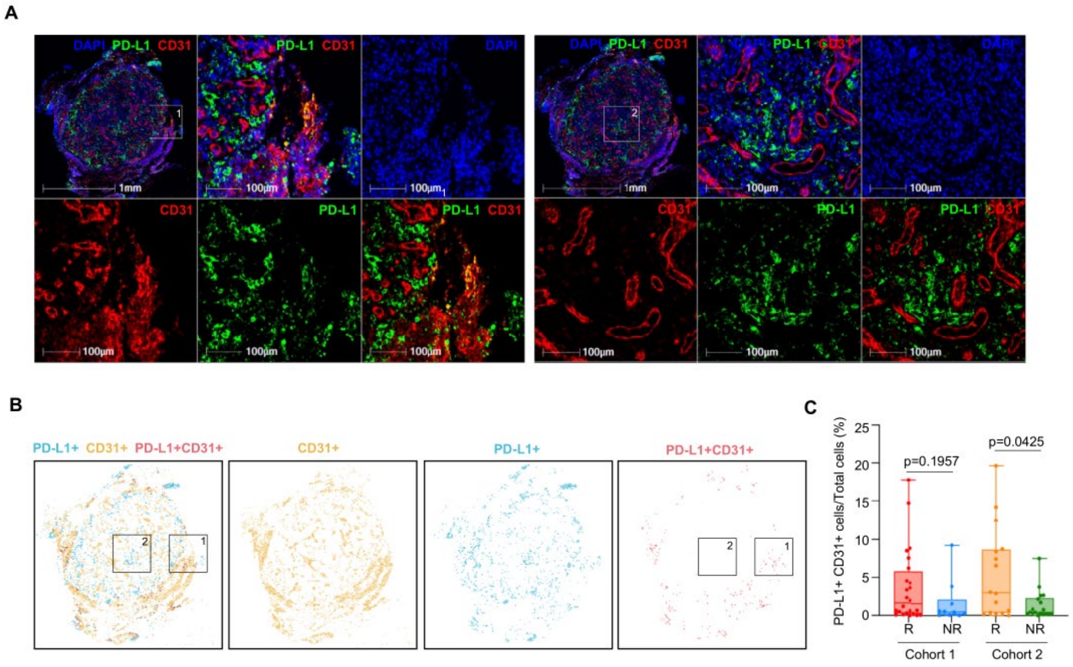

Supplementary Figure 14. PD-L1 was detected on vascular endothelium.

A. Multiplex immunofluorescence assay of PD-L1(green), CD31(red) and DAPI. Original magnification, ×200. Scale bar is 100 μm. B. HALO Spatial Plot of PD-L1+, CD31+ and PD-L1+CD31+ cells; C. Quantification of PD-L1+CD31+ cells in patients with first-line platinum-resistant (cohort 1, n = 10 NRs and 24 Rs) or PD-1 inhibitor resistant (cohort 2, n = 18 NRs and n=16 Rs) recurrent/metastatic nasopharyngeal carcinoma. Responders defined as having complete or partial response by RECIST 1.1 and non-responders as having less than a partial response. Data were presented as mean values ± SD, each dot represented one sample. *P* values were calculated by two-tailed, Mann-Whitney U-test. Source data are provided as a Source Data file.

Supplementary figure 15

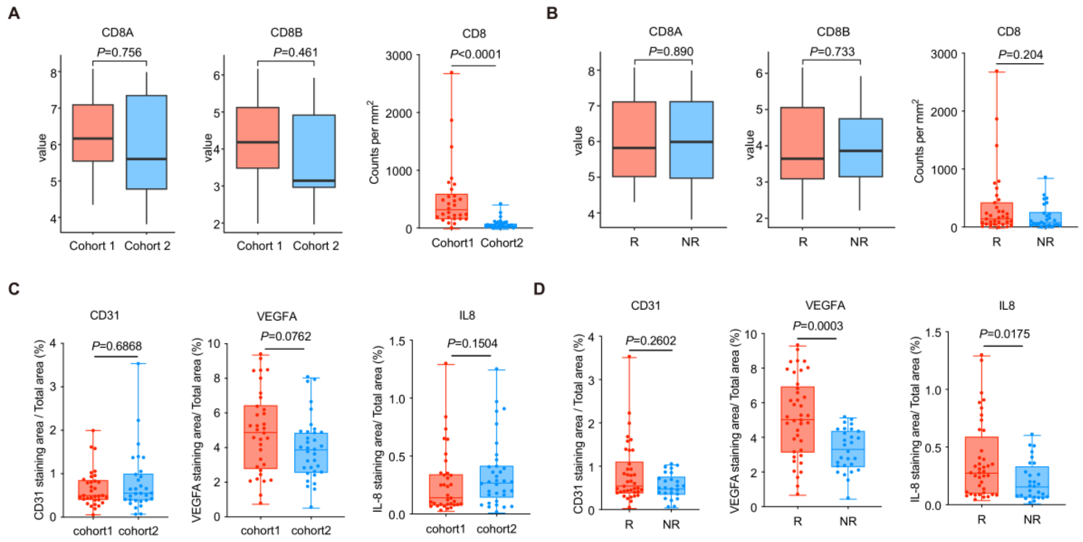

Supplementary Figure 15. CD8 and blood vessel markers expression.

A. CD8 expression in one arm versus another (transcriptomic CD8A, CD8B and IHC CD8 staining) (cohort1 n=29 and cohort2 n=30). B. CD8 expression in responders versus non-responders (transcriptomic CD8A, CD8B and IHC CD8 staining) (response n=33 and non-response n=26); C. Blood vessel markers (CD31, VEGFA and IL8) expression in one arm versus another (cohort1 n=33 and cohort2 n=30); D. Blood vessel markers (CD31, VEGFA and IL8) expression in responders versus non-responders (response n=38 and non-response n=25). All data in box and whiskers plots are represented as median value, quartile, maximum and minimum; each dot represented one sample. *P* values were calculated by two-tailed, Mann-Whitney U-test. Source data are provided as a Source Data file.

Supplementary figure 16

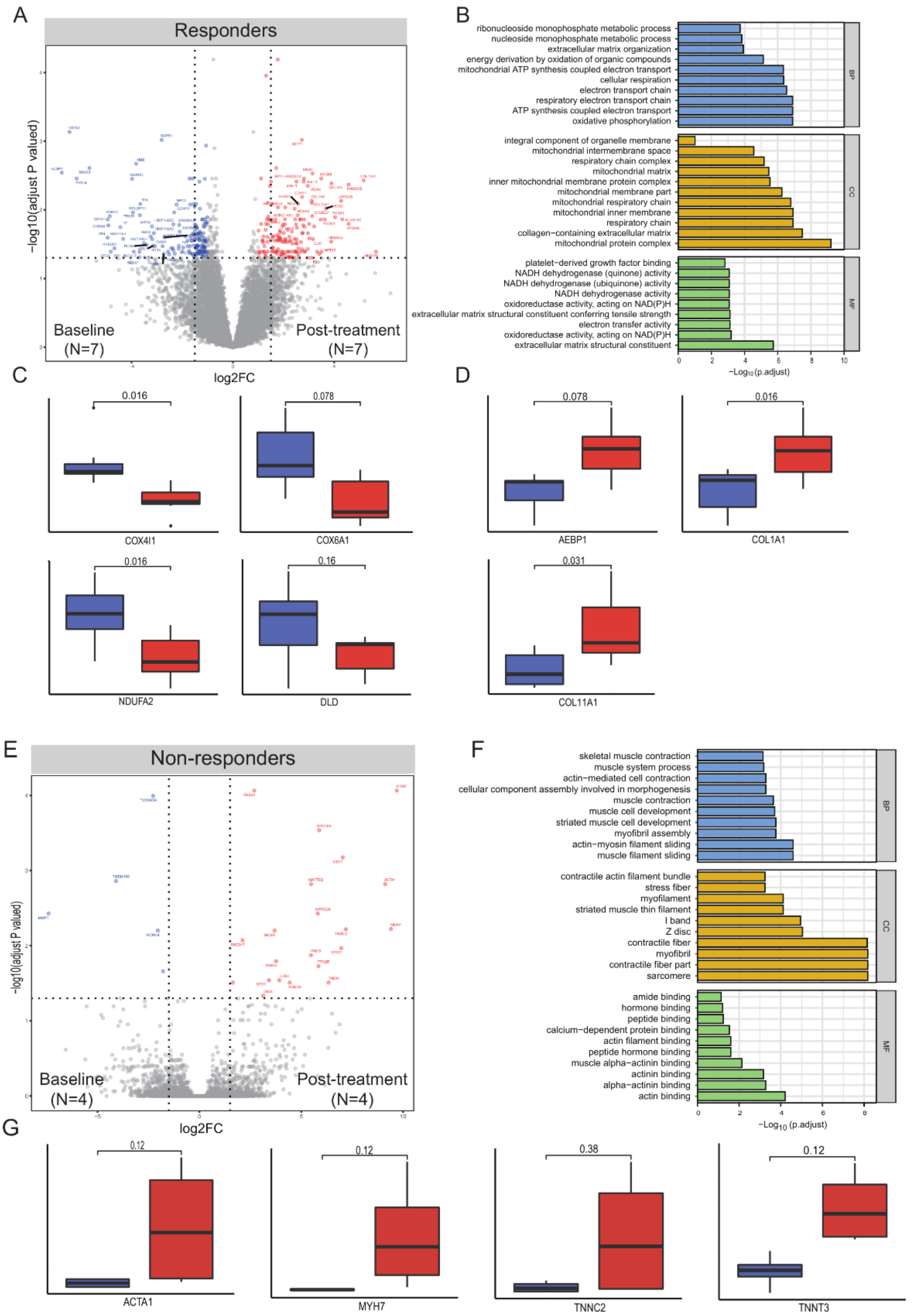

Supplementary Figure 16. Transcriptional analysis of the difference of tumor specimens between baseline and post-treatment in responders and non-responders.

A. Volcano plot depiction of DEGs between baseline (n=7) and post-treatment (n=7) in

responders according to RECIST 1.1. A cutoff fold change (log2-transformed) of ＞2 or ＜−2 and a false discovery rate (FDR) q-value of ＜0.05 were applied. **B.** GO analysis of responders were applied in DEGs with DESeq2-adjusted P value of ＜0.05. **C.** Representative genes which highly expressed in responders at baseline (responders, baseline n=7 and post-treatment n=7). **D.** Representative genes which highly expressed in responders on progression after treatment (responders, baseline n=7 and post-treatment n=7). **E.** Volcano plot depiction of DEGs between baseline (n=4) and post-treatment (n=4) in non-responders according to RECIST 1.1. A cutoff fold change (log2-transformed) of ＞2 or ＜−2 and a false discovery rate (FDR) q-value of ＜0.05 were applied. **F.** GO analysis of non-responders was applied in DEGs with DESeq2-adjusted P value of ＜0.05. **G.** Representative genes which highly expressed in non-responders on progression after treatment (non-responders, baseline n=4 and post-treatment n=4).

*Paired Samples Wilcoxon Signed Rank Test was applied in representative gene comparison between different time point.

Supplementary figure 17

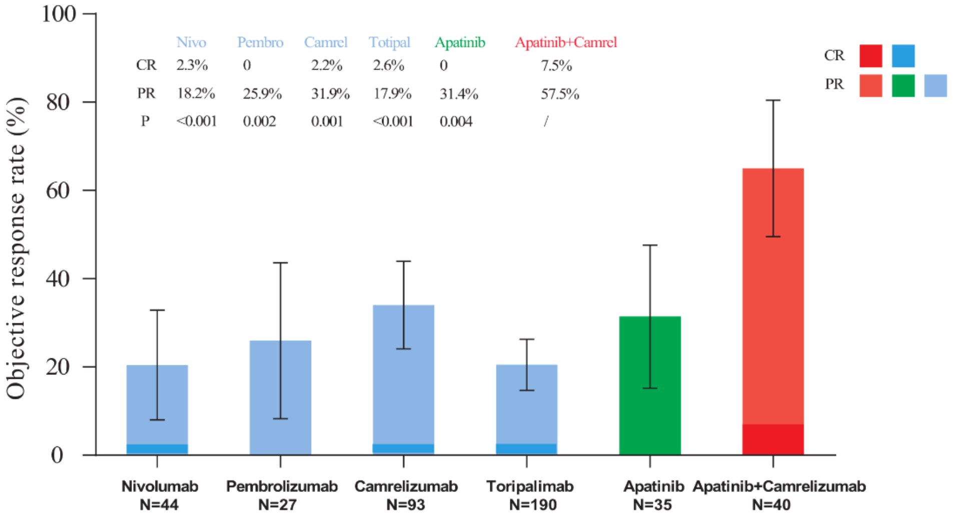

Supplementary Figure 17. Efficacy of anti-PD-1 or apatinib monotherapy, and our combination therapy (cohort 1) in RM-NPC.

A Bar plot shows the proportion of patients with response to pembrolizumab, nivolumab, toripalimab, camrelizumab, apatinib, and camrelizumab plus apatinib. The *P*-value indicates the statistical difference between the ORR of historical monotherapy with PD-1 and apatinib and our studys results.

Supplementary References:

1. Brauer MJ, Zhuang G, Schmidt M, et al. Identification and analysis of in vivo VEGF downstream markers link VEGF pathway activity with efficacy of anti-VEGF therapies. Clin Cancer Res. 2013;19(13):3681-3692.
2. McDermott DF, Huseni MA, Atkins MB, et al. Clinical activity and molecular correlates of response to atezolizumab alone or in combination with bevacizumab versus sunitinib in renal cell carcinoma [published correction appears in Nat Med. 2018 Dec;24(12):1941]. Nat Med. 2018;24(6):749-757.
3. Scheller J, Chalaris A, Schmidt-Arras D, Rose-John S. The pro- and anti-inflammatory properties of the cytokine interleukin-6. Biochim Biophys Acta. 2011;1813(5):878-888.
4. Russo RC, Garcia CC, Teixeira MM, Amaral FA. The CXCL8/IL-8 chemokine family and its receptors in inflammatory diseases. Expert Rev Clin Immunol. 2014;10(5):593-619.
5. Zelenay S, van der Veen AG, Böttcher JP, et al. Cyclooxygenase-Dependent Tumor Growth through Evasion of Immunity. Cell. 2015;162(6):1257-1270.
6. Ha H, Debnath B, Neamati N. Role of the CXCL8-CXCR1/2 Axis in Cancer and Inflammatory Diseases. Theranostics. 2017;7(6):1543-1588.

Supplementary Note 1. Study Protocol

Protocol update information

Study protocol: Efficacy and Safety of Camrelizumab Combined With Apatinib in Patients With First-line Platinum-resistant or PD-1 inhibitor Resistant Recurrent/Metastatic Nasopharyngeal Carcinoma: a Single-center, Phase 2 Trial.

Date of update: January 31, 2021

Section	Previous description	Change
3.3 Number of Subjects	A total of 52 patients were included and divided into two cohorts: Cohort 1 includes 27 patients at least and cohort 2 includes 25 patients at least.<br>(1) Cohort 1: patients with RM-NPC who had failed first-line platinum-based chemotherapy and had not been treated with anti-PD-1 monoclonal antibody.<br>(2) Cohort 2: patients with RM-NPC who failed first-line platinum-based chemotherapy and continued to progress after treatment with anti-PD-1 monoclonal antibody.	A total of 52 patients were included and divided into two cohorts: includes 27 patients in cohort 1 and 25 patients cohort 2. Due to the promising efficacy for the patients treated with camrelizumab plus apatinib both in cohort 1 and cohort 2, many patients took a strong desire to join in this trial and informed consent was signed for some of patients before enrolment was halted. Considering the limited sample in cohort 1 (27 patients) and cohort 2 (25 patients), the stability and reliability of ORR and its 95% confidence intervals (CI) will not be keep stable and reliable due to the small sample size. In order to make more patients will benefit from this trial and continue to evaluate stability and reliability of ORR and its 95% (CI) as the sample size increased, the principal investigator applied to the Research Ethics Committee of Sun Yat-sen University Cancer Center to amend the

		protocol and expanded the sample size, including 13 additional patients in the cohort 1 and 7 in the cohort 2.<br>(1) On January 31, 2021, the protocol was changed to include 13 more cohort 1 patients and 7 additional cohort 2 patients, for a total of 40 cohort 1 patients and 32 cohort 2 patients. Cohort 1(n=40): patients with RM-NPC who had failed first-line platinum-based chemotherapy and had not been treated with anti-PD-1 monoclonal antibody.<br>(2) Cohort 2(n=32): patients with RM-NPC who failed first-line platinum-based chemotherapy and continued to progress after treatment with anti-PD-1 monoclonal antibody.

Efficacy and Safety of Camrelizumab Combined With Apatinib in Patients With First-line Platinum-resistant or PD-1 inhibitor Resistant Recurrent/Metastatic Nasopharyngeal Carcinoma: a Single-center, Phase 2 Trial.

Activation date: July 13, 2020

Last amended: January 31, 2021

Principle Investigator: Prof. Hai-Qiang Mai

Sponsor: Sun Yat-sen University Cancer Center

ClinicalTrials.gov Identifier: cohort 1, NCT04547088 and cohort 2, NCT04548271

Table of contents

Summary of protocol 53
LIST OF ABBREVIATIONS 60
Part I: Study Design and Execution 61
1.0 Background 61
2.0 Study Objectives 68
2.1 Primary Endpoint 69
2.2 Secondary Endpoint 69
2.3 Exploratory Endpoints 69
3.0 Research Design 69
3.1 Research Design Overview 69
3.2 Rationales for Study 70
3.3 Number of Subjects 71
3.4 Research Center 72
4.0 Study Subjects 72
4.1 Target subjects 72
4.2 Inclusion Criteria 72
4.3 Exclusion Criteria 74
4.4 Criteria for Discontinuation 77
4.5 Criteria for Early Withdrawal 78
4.6 Concomitant Drugs and Treatment 78
5.0 Evaluation Schedule 79
5.1 Screening Examination and Qualifying Screening Forms 86
5.2 Procedures for the Inclusion of Qualified Subjects 86
5.3 Clinical Evaluation and Procedures 86
5.4 Laboratory Evaluation 87
5.5 Clinical Treatment After the Study 87
5.6 Subsequent Antitumor Therapy 87
5.7 Follow-up Time 88
6.0 Information on The Investigational Drugs 88
6.1 Dose and Administration Schedule 88

6.2 Preparation and Management of Drugs 89
6.3 Compliance Evaluation 89
7.0 Safety 90
7.1 Adverse Events and Abnormal Laboratory Tests 90
7.2 Security Parameter Processing 94
7.3 Dose Adjustment for Toxicity 95
7.4 Warnings and Precautions 112
8.0 Statistical Matters and Analysis Plans 114
8.1 Primary and Secondary Analysis Endpoints 114
8.2 Sample Size Calculation 115
8.3 Analytical Methods 116
9.0 Data Collection and Management 118
9.1 Requirements for Researchers to Fill Out Clinical Trial Records 118
9.2 Inspector's Examination of Data Records 118
10.0 Quality Control and Quality Assurance 118
10.1 Quality Control 118
10.2 Quality Assurance 119
Part II: Ethics and General Research Management 120
11.0 Ethical Matters 120
11.1 Local Regulations /Declaration of Helsinki 120
11.2 Informed Consent 120
11.3 Independent Ethics Committee /Institutional Review Committee 120
12.0 Conditions of Modifying Protocol 121
13.0 Conditions for Ending the Study 121
14.0 Preservation of Research Documents, Case Reports and Records 121
14.1 Preservation of Researcher Documents /Records 121
14.2 Original Documentation and Background Data 122
14.3 Inspection 122
14.4 Case Report Form 122
15.0 Research Monitoring 122
16.0 Confidentiality of Trial Documents and Patient Records 123
References: 124

Appendix 1) RECIST Version 1.1 ... 128
1. Measurability of tumour at baseline ... 128
1.1. Definitions ... 128
1.2. Specifications by methods of measurements ... 129
2. Tumour response evaluation ... 130
2.1. Assessment of overall tumour burden and measurable disease ... 130
2.2. Baseline documentation of ‘target’ and ‘non-target’ lesions ... 131
2.3. Response criteria ... 132
2.4 Evaluation of response ... 135
Appendix 2) ECOG Performance Status Scale ... 139

Summary of protocol

**Study Title**	Efficacy and Safety of Camrelizumab Combined With Apatinib in Patients With First-line Platinum-resistant or PD-1 inhibitor Resistant Recurrent/Metastatic Nasopharyngeal Carcinoma: a Single-center, Phase 2 Trial.
**Phase**	Phase II
**Objectives**	To evaluate antitumor activity and safety of SHR-1210 plus apatinib for RM-NPC patients after first-line platinum-based chemotherapy failure or having resistance to anti-PD-1 monoclonal antibody.<br>To explore biomarker and the mechanism of SHR-1210 combined apatinib for nasopharyngeal carcinoma immunotherapy.<br>To explore the effect of SHR-1210 combined apatinib on tumor microenvironment.
**Endpoint**	**Primary Endpoint**<br>・Objective response rate (ORR) by Response Evaluation Criteria In Solid Tumors (RECIST) ver1.1
	**Secondary Endpoint**<br>・Safety<br>- The incidences and types of adverse events that occur during treatment will be evaluated according to the National Cancer Institute (NCI) Common Terminology Criteria for Adverse Events (CTCAE) version 5.0.<br>- Acute and chronic toxicity<br>・Efficacy<br>- Duration of response (DoR)<br>- Progression-free survival (PFS)<br>- Overall survival (OS)<br>- Disease control rate (DCR)<br>by Response Evaluation Criteria In Solid Tumors (RECIST) ver1.1

	**Exploratory endpoints**<br>To explore the correlation between PD-L1 and VEGFR-2(KDR) expression in tumor tissue and SHR-1210 antitumor activity. To evaluate the correlation between plasma EBV-DNA changes and SHR-1210 antitumor activity. The RNA sequences were performed to explore biomarkers from the tumor immune contexture that potentially predict the antitumor activity of SHR-1210 and apatinib . To explore the mechanism of SHR-1210 combined apatinib for nasopharyngeal carcinoma immunotherapy. To explore the effect of SHR-1210 combined apatinib on tumor microenvironment.
**Study subjects**	**Inclusion Criteria**<br>1. Written informed consent.<br>2. Histologically or cytologically confirmed with recurrent or metastatic nasopharyngeal carcinoma which is not amenable to curative treatment with surgery and/or radiation therapy.<br>3. Age ≥ 18 years and ≤ 75 years, both genders.<br>4. Eastern Cooperative Oncology Group (ECOG) performance status (PS) 0 or 1.<br>5. The life expectancy of at least 3 months.<br>6. Prior failure of first-line platinum-based chemotherapy (single or combined drugs). Treatment failure is defined as disease progression during or after treatment with platinum-based chemotherapy. For patients who have previously received neoadjuvant chemotherapy, concurrent chemoradiotherapy, or adjuvant chemotherapy, the original treatment is defined as the first-line treatment if recurrence/metastasis occurs within 6 months of the end of the previous treatment. All changes in treatment due to drug intolerance do not count as treatment failure. There is at least one measurable lesion (according to RECIST V1.1), and a measurable lesion that has been treated with radiotherapy and progressed according to radiographic judgment can be considered as a target lesion.<br>7. Cohort 2 only: progression after treatment with PD-1 monoclonal antibody therapy or combined chemotherapy. Treatment failure is defined as disease progression during or after receiving anti-PD-1 monoclonal antibody. There is at

	least one measurable lesion (according to RECIST V1.1). Measurable lesion that has been treated with radiotherapy and progressed according to radiographic judgment can be considered as a target lesion.<br>8. Patients with adequate organ function at the time of enrollment as defined below (No blood components or cell growth factor support therapy is allowed for 2 weeks prior to treatment initiation):<br>a. Absolute neutrophil count ≥ 1.5 × 10^9/L (1,500 mm3); Platelets ≥ 75×10^9/L (100,000 mm3); Hemoglobin > 9 g/dL.<br>b. Serum creatinine ≤ 1.5 × ULN or calculated creatinine clearance ≥ 50 mL/min (Cockcroft-Gault formula).<br>c. Total bilirubin ≤ 1.5 × ULN, AST and ALT ≤ 2.5 × ULN for patients without liver metastases. Total bilirubin ≤ 3.0 × ULN, AST and ALT ≤ 5.0 × ULN for patients with liver metastases.<br>d. Serum albumin ≥ 28 g/L.<br>e. TSH ≤ 1 × ULN (if abnormal, FT3 and FT4 levels should be investigated simultaneously; if FT3 and FT4 levels are normal, they can be included in the group).<br>f. INR and APTT ≤ 1.5 × ULN (unless patient is receiving anticoagulant therapy and coagulation parameters (PT/INR and APTT) are in the expected range of anticoagulant therapy at screening time)<br>9. All women with fertility potential must undergo a urine or serum pregnancy test during screening and the results are negative.
	**Exclusion Criteria**<br>1. Known history of hypersensitivity to any components of the camrelizumab formulation, or other monoclonal antibodies.<br>2. Cohort 1 only: prior exposure to any anti-PD-1, anti-PD-L1, and anti-PD-L2 antibody treatment.<br>3. Prior therapy with tyrosine kinase-inhibitor agent targeting at VEGFR.<br>4. There was a history of severe bleeding, and any bleeding events with a serious

grade of 3 or more in CTCAE5.0 occurred within 4 weeks before screening. Or any other events 4 weeks prior to screening is identified by the investigator as being at high risk of bleeding.

5. Imaging showed that the tumor has invaded important vessels peripherally or the investigator determined that the patients tumor was highly likely to invade important vessels and cause fatal hemorrhage during treatment (retropharyngeal lymph nodes, cervical lymph nodes, hilar lymph nodes, mediastinal lymph nodes, or tumor lesions invaded vascular structures).
6. Patients with necrotizing lesions found on examination within 4 weeks. The investigator judged the risk as massive hemorrhage.
7. Patients with abnormal coagulation function and bleeding tendency (INR must be within the normal range without anticoagulant use 14 days before signing informed consent). Patients who treated with anticoagulants or vitamin K antagonists such as warfarin, heparin, or their analogues are permitted to use low-dose warfarin (1 mg orally, once daily) or low-dose aspirin (100 mg daily or less) for preventive purposes if the INR < 1.5.
8. Patients with arteriovenous thrombosis events, such as cerebrovascular accident (including temporary ischemic attack, cerebral hemorrhage, cerebral infarction), deep venous thrombosis (except venous thrombosis caused by intravenous catheterization due to early chemotherapy) and pulmonary embolism, occurred within 6 months prior to screening.
9. Patients with hypertension who cannot be reduced to the normal range by antihypertensive medication (systolic blood pressure ≥ 140 mmHg or diastolic blood pressure≥90 mmHg which based on mean BP readings from more than two measurements), prior presence of hypertensive crisis or hypertensive encephalopathy.
10. Patients with renal insufficiency: urine routine indicated urine protein ≥ (++) and 24-hour urine protein quantification > 1.0 g.
11. Patients with CYP3A4 inhibitor treatment given within one week before

enrollment, or CYP3A4 inducer treatment given within two weeks before enrollment.

12. Patients with pre-existing or existing inflammatory bowel disease (such as Crohns disease, ulcerative colitis, or chronic diarrhea).
13. Patients with previous or existing history of gastrointestinal perforation and, or, fistula.
14. Patients with disadvantageous factors that affect oral medication (inability to swallow, chronic diarrhea, intestinal obstruction, etc.).
15. Patients with active malignant tumor within the previous 5 years, excluding basal cell carcinoma of the skin and squamous cell carcinoma of the skin after radical treatment, and, or radical resected carcinoma in situ, in addition to the tumors already suffered at enrollment.
16. Patients who currently participating in an interventional clinical study or receiving another study drug within 4 weeks prior to initial administration. Received Chinese herbal or proprietary medicines with antitumor effects within 2 weeks prior to initial administration.
17. Patients with any active autoimmune disease or a history of autoimmune disease (including but not limited to autoimmune hepatitis, interstitial lung disease, uveitis, enteritis, hepatitis, hypophysitis, vasculitis, nephritis, hyperthyroidism, vitiligo; asthma with complete remission in childhood and without any intervention in adulthood could be included to the study; patients with asthma requiring medical intervention with bronchodilators were excluded to the study).
18. Corticosteroids were required within 14 days prior to administration of the study drug (> 10 mg/day equivalent dose of prednisone) or other immunosuppressive drugs for systemic therapy.
19. Patients with congenital or acquired immune deficiency (e.g. HIV positive).
20. Patients with active tuberculosis (TB). Patients suspected of having active TB should be excluded by examination of chest X-rays, sputum, together with clinical signs and symptoms.

	21. Patients with severe infection occurring within 4 weeks of initial administration, including, but not limited to, complications requiring hospitalization, sepsis, or severe pneumonia.<br>22. Patients with HBV DNA > 2000 IU/ml (or 10^4 copies/ml) or HCV RNA > 10^3 copies/ml or HBsAg positive and anti-HCV antibody positive patients;<br>23. Patients with symptomatic central nervous system metastases.<br>24. Severe, uncontrolled angiocardiopathy (heart failure > class II NYHA, unstable angina, myocardial infarction within past 1 year, supraventricular or ventricular arrhythmia which need medical intervention, or QT interval male ≥ 450 ms, female ≥ 470 ms.).<br>25. Live vaccine was administered within 30 days prior to initial administration or was planned for the study period.<br>26. Pregnant or nursing.<br>27. Patients with any condition that, in the investigators opinion, could lead to a risk of receiving the study drug or would interfere with the evaluation of the study drug or with the patients safety or study results interpretation.
**Duration of study participation**	The number of treatment cycles is not fixed. Subjects who achieve complete response (CR), partial response (PR), or stable disease (SD) will receive continuous treatment without withdrawal of their informed consent form, confirmed progression disease, or unacceptable toxicity. Data will be analyzed after the last subject got at least 24 weeks of follow-up (the interval between data cutoff date and first dosing date≥24 weeks). If any subject is still receiving the study drug at that time, the subject may continue receiving the study drug until the investigator confirmed that the subject will no longer benefit, occurrence of progression disease, occurrence of unacceptable toxicity, determination of the investigator, withdrawal of informed consent, or death.
**Number of subjects**	Cohort 1: 27 patients ;Cohort 2: 25 patients<br>On January 31, 2021, the protocol was amended, with an additional 13 patients in cohort 1 and 7 patients in cohort 2 , resulting in a total of 40 patients and 32 patients in cohort 1 and cohort 2 , respectively.

**Administration method**	Cohort 1: SHR-1210 200 mg (every 3 weeks [Q3W], intravenous [IV])<br>Apatinib 250 mg /day orally<br>One cycle will last 21days<br>Cohort 2: Apatinib 250 mg /day orally for the first two weeks.<br>SHR-1210 200 mg (every 3 weeks [Q3W], intravenous [IV])<br>Apatinib 250 mg /day orally<br>One cycle will last 21days
**Safety analysis**	Evaluations will be performed using CTCAE version 5.0.
**Efficacy analysis**	The antitumor effect will be evaluated in accordance with and RECIST version 1.1 every 6 weeks.<br>Image evaluation: computed tomography (CT) or magnetic resonance imaging (MRI)<br>In all patients, ORR, DoR, DCR, PFS and OS will be evaluated
**Trial design**	This trial is a single-center, multi-arm, non-random open label phase II study to evaluate the safety and efficacy of apatinib with SHR-1210 in patients with recurrent or metastatic nasopharyngeal carcinoma who had failed first-line platinum-based chemotherapy or were resistant to Anti-PD-1 monoclonal antibody. The Simon's Optimal two-stage design will be used in this trial.
**Study period**	Planned enrollment period: Aug 2020 to Jun 2021<br>Planned follow-up period: Oct 2021 to Jun 2023
**Principal investigator**	Haiqiang Mai
**Participant center**	Sun Yat-sen University Cancer Center

LIST OF ABBREVIATIONS

AE	Adverse Event
ALT	Alanine Aminotransferase
aPTT	Activated Partial Thromboplastin Time
AST	Aspartate Aminotransferase
BUN	Blood Urea Nitrogen
CR	Complete Response
CT	Computed Tomography
CTCAE	Common Terminology Criteria for Adverse Events
CTLA-4	Cytotoxic T-Lymphocyte-Associated Protein 4
DC	Dendritic Cell
DRSAE	Drug-Related Serious Adverse Event
EBV	Epstein-Barr Virus
ECOG	Eastern Cooperative Oncology Group
HIV	Human Immunodeficiency Virus
irAE	Immune-related Adverse Event
MRI	Magnetic Resonance Imaging
NCI	National Cancer Institute
NPC	Nasopharyngeal Carcinoma
ORR	Overall Response Rate
OS	Overall Survival
PD	Progressive Disease
PD-1	Programmed Death-1
PD-L1	Programmed Death Ligand-1
PET	Positron Emission Tomography
PFS	Progression-free Survival
PR	Partial Response
RECIST	Response Evaluation Criteria In Solid Tumors
SAE	Serious Adverse Event
SD	Stable Disease
TSH	Thyroid-Stimulating Hormone
VEGFR	Vascular Epidermal Growth Factor Receptor
WBC	White Blood Cells

Part I: Study Design and Execution

1.0 Background

Nasopharyngeal carcinoma (NPC) is a malignant tumor originating from the epithelium of the nasopharynx with an unbalanced geographical distribution. According to GLOBOCAN 2018 published by the International Agency for Research on Cancer (IARC), 70% of new cases of NPC occur in East and Southeast Asia, and China accounts for 47.7% of new cases worldwide[1]. As Cancer Statistics in China, 2015 published in CA: A Cancer Journal for Clinicians by Hao et al. shown, China has a high incidence of nasopharyngeal cancer, with approximately 60,000 new cases of NPC reported in 2015, including 34,000 deaths[2]. Radiation is the most important therapy for NPC. With the rapid development of computer technology, imaging diagnosis and extensively applied in clinical practice at the end of the 20th century, radiation therapy has entered the era of modern precision radiotherapy represented by intensity modulated radiation therapy (IMRT). Since IMRT can significantly increase the radiation dose of target area and reduce the radiation dose of surrounding normal tissues and organs, it has become the main radiation therapy of NPC. With the development of the radiation therapy, the local control rate of NPC has significantly improved. However, locoregional recurrence occurs in 10%-15% of patients[3-7]. Besides, distant metastasis occurs in 10% of untreated NPC patients[8-9] and distant metastasis still occurs in 30% of patients with locally advanced NPC after treatment[9]. Locoregional recurrence and distant metastasis have already become the main reasons for the failure of NPC treatment, which are urgent clinical problems in the treatment of NPC. On account of the large heterogeneity of recurrent/metastatic NPC(RM-NPC) and plan of treatment lack of standardization, patients' prognoses vary widely. Both the treatment of recurrent NPC and the treatment of metastatic NPC are difficult, which are major challenges for oncologists to implement treatment strategies.

GEM20110714, a muti-center, random, open-label Phase III Study, compared gemcitabine plus cisplatin with fluorouracil plus cisplatin as first-line therapy for RM-NPC, registered with ClinicalTrials.gov identifier: NCT01528618. The study showed

that the progression-free survival (PFS) was significantly longer with GP than with FP (7.0 months versus 5.6 months), the overall survival (OS) was significantly longer with GP than with FP (29.1 months versus 20.1 months), the objective response rate (ORR) was significantly higher with GP than with FP (64.1% versus 42.0%)[10]. Gemcitabine or cisplatin has been confirmed as the standard first-line treatment of RM-NPC by the NCCN guidelines for head and neck cancer. Other recommended therapy plans include fluorouracil, paclitaxel, docetaxel, carboplatin, cetuximab and so on. As we have learned, at present, the ORR of first-line chemotherapy was only 50%-60%, the average PFS was only 6-7 months and the average OS was round to 2 years. Even if patients accepted second-line chemotherapy, the ORR was only 10%-20%, duration of disease control was only 3-4 months and the average OS was about only 1 year. Although patients with RM-NPC experience disease remission after first-line treatment, chemotherapy responses are often transitory and most patients experience disease progression, which implies that the effect of chemotherapy is not so well as expected[11]. For second-line treatment of RM-MPC, no preferred treatment is currently recommended in the NCCN guidelines. Except for first-line treatment of chemotherapy, immune checkpoint inhibitors are recommended for treatment. RM-NPC patients often face an embarrassing situation of lack of effective second-line treatment drugs once the first-line chemotherapy fails. It is an urgent need for patients with RM-NPC to explore new and effective treatment strategies to improve efficacy and prolong survival time.

Immune checkpoint inhibitor, an innovative treatment, has already become a hot topic in the research field of tumor therapy. By combining immune checkpoints, immune checkpoint inhibitors remove the tolerance of the immune system to tumor cells, activate T cell, improve tumor cell recognition and killing, and restart the tumor immune cycle. Currently, tumor immune checkpoint targets that have shown significant clinical efficacy include cytotoxic T lymphocyte-associated antigen-4 (CTLA-4) and programmed cell death receptor 1/ligand 1 (PD-1/PD-L1). Up to now, the National Medical Products Administration has approved 6 PD-1/PD-L1 monoclonal antibody products for marketing, namely Bristol Myers Squibb’s anti-PD-1 monoclonal antibody Nivolumab (Opdivo), Merck’s anti-PD-1 monoclonal antibody Pembrolizumab

(Keytruda), TopAlliance's anti-PD-1 monoclonal antibody Toripalimab (JS001), Innovent's anti-PD-1 monoclonal antibody Sintilimab, Hengrui's anti-PD-1 monoclonal antibody Camrelizumab (SHR-1210) and AstraZenecas anti -PD-L1 monoclonal antibody Durvalumab, approved indications include advanced melanoma, advanced non-small cell lung cancer and advanced classic Hodgkin lymphoma. PD-1 is expressed on the surface of activated T cells, and the combination of PD-1 and PD-L1 significantly inhibits the proliferation and activation of T cells, resulting in immunosuppression. Previous clinical research reported that PD-L1 overexpression was associated with poor prognosis of tumors, while blocking PD-1 signal could improve the prognosis of patients with malignant tumors[12-13]. NPC is a tumor with high incidence in China. Its main pathological classifications are WHOII&III that are associated with Epstein-Barr virus infection, which is manifested by intensive lymphocyte infiltration and increased PD-L1 expression. Since 89%-95% NPC cells' surfaces express PD-L1, which implies suboptimal outcomes of conventional chemotherapy and favorable PD-L1 antibody effect, creates conditions for immune checkpoint inhibitor therapy[14-15]. KEYNOTE-028, a phase Ib clinical trial (NCT02054806), reported the anti-tumor activity and safety of Pembrolizumab (Keytruda) as a treatment of RM-NPC. In 27 patients with PD-L1 positive RM-NPC who received 10 mg/kg Pembrolizumab once every two weeks, after being evaluated by the investigator, the ORR was 25.9%, the median PFS was 17.1 months, along with one unconfirmed partial response. After being evaluated by the IRRC, partial response was achieved in 5 of 19 subjects (26.3%). The median OS was 16.5 months and 1-year OS rate was 63%[14]. NCI-9472, a phase II clinical trial (NCT02339558), evaluated the clinical efficacy of the immune checkpoint inhibitor Nivolumab treating RM-NPC. In 44 patients with RM-NPC who received 3 mg/kg Nivolumab once every two weeks, after being evaluated by the investigator, the ORR was 20.5%, median duration of response was 9.3 months, median OS was 17.1 months and one-year OS rate was 59%. The ORR was 33% in 18 PD-L1 positive patients, while among 23 PD-L1 negative patients, the ORR was 13%[16]. According to the latest clinical progress and data of POLARIS-02 (NCT02915432) reported at ASCO 2019, using TopAlliance's

toripalimab (JS001) in Chinese patients with metastatic NPC who had previously received systemic treatment and failed, the enrolled patients received 3 mg/kg JS001 once every two weeks. As of May 10, 2019, a total of 165 patients underwent at least one post-treatment evaluation, with clinical efficacy evaluated by the investigator: 4 patients achieved CR, 38 patients achieved PR, 48 patients achieved SD, the ORR was 25.5%, the disease control rate (DCR) was 47.1% and the mean time to onset of response was 3.4 months. The median duration of response had not yet been reached, as 22 of 42 patients who responded to treatment continued to respond. Another research about camrelizumab (SHR-1210), a phase I clinical trial (NCT02721589), evaluated whether immune checkpoint inhibitor is a potentially effective and well-tolerated treatment option for RM-NPC patients that progressed after first-line therapy. Patients were treated biweekly at 1 mg/kg, 3 mg/kg, 10 mg/kg, or 200 mg SHR-1210, resulting the ORR of 34% and the DCR of 59% in 91 patients. 2 (2%) patients achieved CR, 29 (32%) patients achieved PR, 23 (25%) patients achieved SD, and 37 (40%) patients experienced PD. The median PFS was 5.6 months[17]. The above anti-PD-1 monoclonal antibody therapy for RM-NPC reported ORR was about 20% -30%, and the toxic and side effects can be tolerated. Recommended by the latest NCCN guidelines, Nivolumab can be used as second or later-line therapy with PD-L1 positive and non-keratinizing RM-NPC (class 2B evidence) and Pembrolizumab can be used as second or later-line therapy for PD-L1 positive RM-NPC (class 2B evidence). Immune checkpoint inhibitor therapy has opened a new era for the treatment of refractory NPC, but the efficacy of single immune checkpoint inhibitor therapy for RM-NPC is extremely limited, and the guidelines are also lack of corresponding recommended treatment after NPC cells get anti-PD-1 monoclonal antibody resistance. Therefore, immunotherapy for refractory NPC combined with other treatments to improve the control of the disease is an urgent treatment strategy to be explored.

With the further understanding of tumor pathophysiology and the identification of cancer-related molecular biomarkers, targeted antitumor drugs have emerged. Abnormal angiogenesis is a key feature of tumor growth and metastasis, therefore drugs that inhibit tumor angiogenesis offer new strategies for treatment. The mechanism of

antiangiogenic drugs is as followed: (1) degenerate existing tumor blood vessels, thus cut off oxygen and other nutrients needed for tumor cell growth; (2) normalize surviving tumor vessels, reduce interstitial pressure, and improve the delivery of therapeutic drugs to tumor tissues; (3) inhibit tumor angiogenesis, thus continuously inhibit the growth and metastasis of tumor cells. Vascular endothelial growth factor (VEGF) pathway is the key to angiogenesis[18]. Previous clinical studies have shown that the expression level of VEGF in NPC tissues is correlated with the prognosis of NPC patients. Studies have found that VEGF is positively correlated with T stage, lymph node metastasis, N stage and clinical stage, and negatively correlated with the survival rate. VEGF expression is an independent risk factor affecting the prognosis of NPC patients[19]. Other studies have found that the expression of VEGF is related to NPC angiogenesis, lymph node metastasis[20], and distant metastasis[21]. VEGF expression occurs in more than 60% of NPC patients undergoing clinical biopsy[22]. Vascular endothelial growth factor receptor (VEGFR) inhibitors have been used as targeted drug therapy for NPC, and apatinib has shown strong clinical effects. Apatinib is an oral small molecule tyrosine protease inhibitor, which mainly inhibits the activity of tyrosine kinase and blocks the signal transduction pathway of VEGF binding to its receptor by acting on VEGFR-2, thereby inhibiting tumor angiogenesis, tumor growth and tumor progression[23]. Previous clinical studies have confirmed that apatinib shows anti-tumor activity and tolerable toxicity in gastric adenocarcinoma, ovarian cancer, breast cancer and other malignancies[24-26], and it was approved by National Medical Products Administration in 2014. A prospective multi-arm open-label phase II clinical trial (NCT02867956) conducted by Huang et al., is about apatinib plus etoposide in the treatment of platinum-resistant ovarian epithelial carcinoma. A total of 35 patients with platinum-resistant epithelial ovarian cancer received a starting dose of 500 mg apatinib once daily and oral etoposide starting dose of 50 mg, once daily (days 1-14, repeat every 21 days). Oral etoposide was administered for a maximum of 6 courses, after which apatinib therapy was maintained until disease progression or toxicity becomes intolerable. As the study showed, in the intent to treat (ITT) population, the ORR was 54.3% and the DCR was 85.7%. The study has been published in the Lancet Oncology,

and has been widely recognized by the academic community[27]. In an open-label, single-arm, exploratory study, 51 patients with RM-NPC who had progressed after failure of the first-line platinum-containing chemotherapy and second-line single or combined chemotherapy were given apatinib(500 mg/ day). The result showed that 16 (31.37%) patients had achieved ORR, with 0 cases of CR, 16 cases (31.37%) of PR, 10 cases (19.61%) showed SD and 25 cases (49.02%) showed PD. The median OS was 16 months (95% CI, 9.32–22.68) and the median PFS was 9 months (95% CI, 5.24–12.76)[28]. Data from these studies confirmed that apatinib has good efficacy and controllable safety in patients with advanced NPC that has progressed after first-line chemotherapy. Given that apatinib is well tolerated in patients with RM-NPC and can achieve long-term disease control, further studies can be conducted in a maintenance setting.

Studies have shown that both immunotherapy and anti-angiogenic targeted therapy act on the tumor microenvironment (TME), and there is a complex interaction between tumor immune microenvironment reprogramming and tumor vascular remodeling. VEGF released from TME can alter the expression of intercellular cell adhesion molecule-1 (ICAM1) and vascular cell adhesion molecule-1 (VCAM1) on endothelial cells (ECs) and immune cells which leads to reduce T cell transport to tumor[29-30]. VEGF can enhance the aggregation, activation and proliferation of regulatory T cells (Treg), promote the recruitment of tumor-associated macrophages (TAM) and the reprogramming of TAM to M2 phenotype, and promote the proliferation of myeloid-derived suppressor cells (MDSC), thus achieving immunosuppression[31-32]. These cells continue to release VEGF and thus activate autocrine and paracrine pathways. VEGF can also directly inhibit cytotoxic lymphocyte (CTL) related to tumor immunity[33]. Dendritic Cells (DC), which are essential for CTL maturation, lose their ability to mature and release antigen after exposure to VEGF[34]. In conclusion, immunosuppressive cells eventually established a tumor-friendly microenvironment by inhibiting CTL under the effect of VEGF. In addition, it was found that anti-angiogenic drugs can block VEGF-induced immune checkpoint expression, and anti-VEGF therapy can selectively inhibit the expression of immune checkpoint molecules such as

PD-1, CTLA-4 and T cell immunoglobulin-3 (TIM-3) on CTL surface, since VEGF can up-regulate PD-1 expression by activating VEGFR2-PLC γ-calcineurin-NFAT signaling pathway[33]. Therefore, anti-PD-1 combined with anti-VEGF therapy can effectively block the PD-1/PD-L1 axis and synergically inhibit tumor growth, especially in tumors with high VEGF secretion. Anti-angiogenic drugs that inhibit VEGF and VEGFR signal transduction pathway can change the disorder of tumor blood vessels, normalize blood vessels, improve tissue perfusion, improve hypoxia environment, and enhance the infiltration of immune cells to tumor, and relieve immune suppression. Normalization of tumor blood vessels can enhance the efficacy of immunotherapy and positively regulate the immunity of the body. Activation of immune cell function/immune reprogramming can also adversely affect tumor blood vessels and promote vascular and TME remodeling[35-39]. Thus, tumor vascular normalization and immune reprogramming have a synergistic effect and can enter a mutually reinforcing virtuous cycle by improving TME. The IMpower150 study (NCT02366143), a multi-center, open-label, randomized, controlled phase III clinical trial, evaluated atezolizumab plus chemotherapy plus bevacizumab versus chemotherapy plus bevacizumab in the first-line treatment of metastatic non-squamous NSCLC. Results of this study presented at the 2019 European Lung Cancer Congress (ELCC) showed that a total of 1202 newly treated patients with advanced NSCLC were enrolled. In the ITT-WT population, the atezolizumab plus chemotherapy plus bevacizumab group had a longer median OS (19.8 months vs 14.9 months), and an improved median PFS (8.4 months vs 6.8 months) and an improved ORR (56% vs 40%) were also observed compared to the chemotherapy plus bevacizumab group[40]. IMpower150 is the first phase III clinical trial of immunotherapy combined anti-angiogenesis therapy combined with chemotherapy, and has reached the end point of the study. Clinically, it has been confirmed that immunotherapy combined anti-angiogenesis therapy has synergistic effect, which provides important inspiration and confidence for the subsequent similar studies. A phase I clinical trial (NCT02942329), conducted by Xu et al., focused on SHR-1210 plus apatinib for advanced hepatocellular carcinoma (HCC), gastric or esophagogastric junction cancer, enrolled 43 patients.

Phase Ia dose climb results showed that the recommended dose of apatinib was 250 mg/d, and in phase Ib apatinib (250 mg/d) was used in combination with the immune checkpoint inhibitor SHR1210 (200 mg/Q2W). The ORR of 39 assessable patients was 30.8% and the disease response rate was 84.6%, among which the ORR of 16 HCC patients reached 50%[41], showing encouraging efficacy. At present, SHR-1210 combined with apatinib has been used in advanced liver cancer (NCT03463876), non-squamous non-small cell lung cancer (NCT03083041), extensive-stage small cell lung cancer (NCT03417895), triple negative breast cancer (NCT03394287), and gastric/gastroesophageal junction carcinoma (NCT03472365), esophageal squamous cell carcinoma (NCT03603756), osteosarcoma (NCT03359018), cervical cancer (NCT03816553) and other clinical trial.

In conclusion, tumor immune checkpoint inhibitors have made major breakthroughs in a variety of solid tumors, and currently NCCN guidelines also recommend Nivolumab and Pembrolizumab as second or later-line treatment for PD-L1 positive RM-NPC. There are increasing evidences that immunotherapy combined with antiangiogenic therapy has synergistic effects, and SHR-1210 combined with apatinib has shown initial success in a variety of solid tumors. Therefore, the purpose of our study was to evaluate the efficacy and safety of SHR-1210 plus apatinib in the treatment of RM-NPC with failure of first-line platinum-based chemotherapy or resistance to PD-1 monoclonal antibody, and to explore whether SHR-1210 plus apatinib can be used as the second-line or subsequent line treatment for RM-NPC in order to explore and establish an effective and individualized comprehensive treatment regimen for refractory RM-NPC with first-line chemotherapy failure or anti-PD-1 monoclonal antibody resistance.

2.0 Study Objectives

To evaluate antitumor activity and safety of SHR-1210 with apatinib for RM-NPC patients after first-line platinum-based chemotherapy failure or having resistance to anti-PD-1 monoclonal antibody. To explore biomarker and the mechanism of SHR-1210 combined apatinib for nasopharyngeal carcinoma immunotherapy. To explore the effect of SHR-1210 combined apatinib on tumor microenvironment.

2.1 Primary Endpoint

・Objective response rate (ORR) by Response Evaluation Criteria In Solid Tumors (RECIST) ver1.1

2.2 Secondary Endpoint

・**Safety**

- The incidences and types of adverse events that occur during treatment will be evaluated according to the National Cancer Institute (NCI) Common Terminology Criteria for Adverse Events (CTCAE) version 5.0.
- Acute and chronic toxicity

・**Efficacy**

- Duration of response (DoR)
- Progression-free survival (PFS)
- Overall survival (OS)
- Disease control rate (DCR) by Response Evaluation Criteria in Solid Tumors (RECIST) ver1.1

2.3 Exploratory Endpoints

To explore the correlation between PD-L1 and VEGFR-2(KDR) expression in tumor tissue and SHR-1210 antitumor activity. To evaluate the correlation between plasma EBV-DNA changes and SHR-1210 antitumor activity. To explore biomarkers (mRNA) that potentially predict the antitumor activity of SHR-1210, including but not limited to tumor mutation load (TMB).

3.0 Research Design

3.1 Research Design Overview

3.1.1 Administration Method

(1) Cohort 1: SHR-1210 is given 200 mg IV every 3 weeks and apatinib is given 250 mg every day orally, continuing to give drugs for a 3-week treatment cycle. Until a treatment discontinuation event specified in the protocol occurs (an unacceptable toxicity, or disease progression, or withdrawal of consent, or the investigators judgment that the patient needs to withdraw from treatment, or a

maximum treatment period of 2 years).

(2) Cohort 2: Apatinib is given 250 mg orally every day in the first two weeks. Followed by SHR-1210 given 200 mg IV every 3 weeks and apatinib given 250 mg every day orally, continuing to give drugs for a 3-week treatment cycle. Until a treatment discontinuation event specified in the protocol occurs (an unacceptable toxicity, or disease progression, or withdrawal of consent, or the investigators judgment that the patient needs to withdraw from treatment, or a maximum treatment period of 2 years).

3.1.2 Analysis Plan

After the patients are enrolled in the study, safety visits will conduct for each treatment cycle D1 before medication. Tumor evaluation will perform every 6 weeks (±7 days) from the beginning of medication until patients have confirmed disease progression, withdrawal of informed consent forms (ICF), death, or study closure, whichever occurred first. Possible adverse events (AE) will be monitored throughout the trial and graded in severity according to the guidelines of the NCI-CTCAE version 5.0. After treatment, subjects will be followed up for 30 days to monitor AE.

3.2 Rationales for Study

3.2.1 Rationales for Study Design

Clinical data can confirm that the ORR of anti-PD-1 monoclonal antibody therapy for the treatment of RM-NPC was about 20%-30%, and the toxic and side effects can be tolerated. The latest NCCN guidelines recommend anti-PD-1 monoclonal antibody as second-line therapy for PD-L1 positive RM-NPC (class 2B evidence). Apatinib alone has a good efficacy and controllable safety in patients with advanced NPC who progressed after first-line chemotherapy. Basic studies have demonstrated that tumor vascular normalization and immune reprogramming have a synergistic effect and can enter a mutually reinforcing virtuous cycle by improving the tumor microenvironment. There are increasing evidences that immunotherapy combined with antiangiogenic therapy has synergistic effects, and SHR-1210 combined with apatinib has shown initial success in a variety of solid tumors.

3.2.2 Rationales for Dose Selection

In a phase I clinical trial (NCT02942329), phase Ia dose climb results showed that the recommended dose of apatinib was 250 mg/d in patients with advanced liver cancer and gastric or esophagogastric junction cancer. After a certain period of time, apatinib treatment with lower than the standard treatment dose can induce normalization of abnormal blood vessels in the tumor, improve hypoxia, and reshape the immunosuppressive microenvironment of the tumor to favor the immune-supportive phenotype, which will be conducive to immunotherapy efficacy. In a Phase Ib study of NSCLC (NCT03083041), patients were treated with SHR-1210 (200 mg/Q2W) plus apatinib (250 mg/d, 375 mg/d, or 500 mg/d). The results showed that the low-dose apatinib group (250 mg/d) alleviated tissue hypoxia, increased CD8+ T cell infiltration, and reshaped the immunosuppressive tumor microenvironment to make it more conducive to immunotherapy, thereby slowing down tumor growth. In the high-dose group (500 mg/d) and medium-dose group (375 mg/d), although apatinib inhibited tumor growth more significantly, CD8+ T cell infiltration was significantly reduced and tissue hypoxia was aggravated, making the tumor microenvironment not conducive to PD-1/PD-L1 monoclonal antibody therapy. Therefore, apatinib 250 mg/d was used in this study. Information about SHR-1210 refer to the medicine instruction.

3.2.3 End of Study

End of study refers to the date of the last patients last visit or the date of the last data point required for efficacy analysis or safety follow-up, whichever occurs later. The primary end point will be analyzed after the last subject completed at least 24 weeks of follow-up (the interval between data cutoff date and initial dosing date ⩾ 24 weeks). In addition, the sponsor may at any time decide to terminate the study. The total length of the study from the first patient screening to the end of the study is expected to be approximately 24 months. At the end of the study, the sponsor will continue to supply drugs to patients who are still under treatment.

3.3 Number of Subjects

A total of 52 patients at least were included and divided into two cohorts: includes 27 patients in cohort 1 and 25 patients cohort 2. Due to the promising efficacy for the

patients treated with camrelizumab plus apatinib both in cohort 1 and cohort 2, many patients took a strong desire to join in this trial and informed consent was signed for some of patients before enrolment was halted. Considering the limited sample in cohort 1 (27 patients) and cohort 2 (25 patients), the stability and reliability of ORR and its 95% confidence intervals(CI) will not be keep stable and reliable due to the small sample size. In order to make more patients will benefit from this trial and continue to evaluate stability and reliability of ORR and its 95% (CI) as the sample size increased, the principal investigator applied to the Research Ethics Committee of Sun Yat-sen University Cancer Center to amend the protocol and expanded the sample size, including 13 additional patients in the cohort 1 and 7 in the cohort 2.

(1) On January 31, 2021, the protocol was changed to include 13 more cohort 1 patients and 7 additional cohort 2 patients, for a total of 40 cohort 1 patients and 32 cohort 2 patients. Cohort 1(n=40): patients with RM-NPC who had failed first-line platinum-based chemotherapy and had not been treated with anti-PD-1 monoclonal antibody.

(2) Cohort 2(n=32): patients with RM-NPC who failed first-line platinum-based chemotherapy and continued to progress after treatment with anti-PD-1 monoclonal antibody.

3.4 Research Center

This study was a single-center trial conducted in Sun Yat-sen University Cancer Center.

4.0 Study Subjects

4.1 Target subjects

(1) Patients with RM-NPC who had failed first-line platinum-based chemotherapy and had not been treated with anti-PD-1 monoclonal antibody.

(2) Patients with RM-NPC who had failed first-line platinum-based chemotherapy and continued to progress after treatment with anti-PD-1 monoclonal antibody.

4.2 Inclusion Criteria

Patients must meet all of the following criteria to be eligible for inclusion in the study:

1. Written informed consent.
2. Histologically or cytologically confirmed with recurrent or metastatic nasopharyngeal carcinoma which is not amenable to curative treatment with surgery and/or radiation therapy.
3. Age ⩾ 18 years and ⩽ 75 years, both genders.
4. Eastern Cooperative Oncology Group (ECOG) performance status (PS) 0 or 1.
5. The life expectancy of at least 3 months.
6. Prior failure of first-line platinum-based chemotherapy (single or combined drugs). Treatment failure is defined as disease progression during or after treatment with platinum-based chemotherapy. For patients who have previously received neoadjuvant chemotherapy, concurrent chemoradiotherapy, or adjuvant chemotherapy, the original treatment is defined as the first-line treatment if recurrence/metastasis occurs within 6 months of the end of the previous treatment. All changes in treatment due to drug intolerance do not count as treatment failure. There is at least one measurable lesion (according to RECIST V1.1), and a measurable lesion that has been treated with radiotherapy and progressed according to radiographic judgment can be considered as a target lesion.
7. Cohort 2 only: progression after treatment with PD-1 monoclonal antibody therapy or combined chemotherapy. Treatment failure is defined as disease progression during or after receiving anti-PD-1 monoclonal antibody. There is at least one measurable lesion (according to RECIST V1.1). Measurable lesion that has been treated with radiotherapy and progressed according to radiographic judgment can be considered as a target lesion.
8. Patients with adequate organ function at the time of enrollment as defined below (No blood components or cell growth factor support therapy is allowed for 2 weeks prior to treatment initiation):
   a. Absolute neutrophil count ≥ 1.5 × 10^9/L (1,500 mm3); Platelets ≥ 75×10^9/L (100,000 mm3); Hemoglobin > 9 g/dL.
   b. Serum creatinine ≤ 1.5 × ULN or calculated creatinine clearance ≥ 50 mL/min

(Cockcroft-Gault formula).

c. Total bilirubin ≤ 1.5 × ULN, AST and ALT ≤ 2.5 × ULN for patients without liver metastases. Total bilirubin ≤ 3.0 × ULN, AST and ALT ≤ 5.0 × ULN for patients with liver metastases.

d. Serum albumin ≥ 28 g/L.

e. TSH ≤ 1 × ULN (if abnormal, FT3 and FT4 levels should be investigated simultaneously; if FT3 and FT4 levels are normal, they can be included in the group).

f. INR and APTT ≤ 1.5 × ULN (unless patient is receiving anticoagulant therapy and coagulation parameters (PT/INR and APTT) are in the expected range of anticoagulant therapy at screening time)

9. All women with fertility potential must undergo a urine or serum pregnancy test during screening and the results are negative.

4.3 Exclusion Criteria

Patients who meet any of the following criteria will not be eligible for inclusion in the study:

1. Known history of hypersensitivity to any components of the camrelizumab formulation, or other monoclonal antibodies.
2. Cohort 1 only: prior exposure to any anti-PD-1, anti-PD-L1, and anti-PD-L2 antibody treatment.
3. Prior therapy with tyrosine kinase-inhibitor agent targeting at VEGFR.
4. There was a history of severe bleeding, and any bleeding events with a serious grade of 3 or more in CTCAE5.0 occurred within 4 weeks before screening. Or any other events 4 weeks prior to screening is identified by the investigator as being at high risk of bleeding.
5. Imaging showed that the tumor has invaded important vessels peripherally or the investigator determined that the patients tumor was highly likely to invade important vessels and cause fatal hemorrhage during treatment (retropharyngeal lymph nodes, cervical lymph nodes, hilar lymph nodes, mediastinal lymph nodes, or tumor lesions invaded vascular structures).

6. Patients with necrotizing lesions found on examination within 4 weeks. The investigator judged the risk as massive hemorrhage.
7. Patients with abnormal coagulation function and bleeding tendency (INR must be within the normal range without anticoagulant use 14 days before signing informed consent). Patients who treated with anticoagulants or vitamin K antagonists such as warfarin, heparin, or their analogues are permitted to use low-dose warfarin (1 mg orally, once daily) or low-dose aspirin (100 mg daily or less) for preventive purposes if the INR＜1.5.
8. Patients with arteriovenous thrombosis events, such as cerebrovascular accident (including temporary ischemic attack, cerebral hemorrhage, cerebral infarction), deep venous thrombosis (except venous thrombosis caused by intravenous catheterization due to early chemotherapy) and pulmonary embolism, occurred within 6 months prior to screening.
9. Patients with hypertension who cannot be reduced to the normal range by antihypertensive medication (systolic blood pressure ≥ 140 mmHg or diastolic blood pressure≥90 mmHg which based on mean BP readings from more than two measurements), prior presence of hypertensive crisis or hypertensive encephalopathy.
10. Patients with renal insufficiency: urine routine indicated urine protein ≥ (++) and 24-hour urine protein quantification ＞1.0 g.
11. Patients with CYP3A4 inhibitor treatment given within one week before enrollment, or CYP3A4 inducer treatment given within two weeks before enrollment.
12. Patients with pre-existing or existing inflammatory bowel disease (such as Crohns disease, ulcerative colitis, or chronic diarrhea).
13. Patients with previous or existing history of gastrointestinal perforation and, or, fistula.
14. Patients with disadvantageous factors that affect oral medication (inability to swallow, chronic diarrhea, intestinal obstruction, etc.).
15. Patients with active malignant tumor within the previous 5 years, excluding basal

cell carcinoma of the skin and squamous cell carcinoma of the skin after radical treatment, and, or radical resected carcinoma in situ, in addition to the tumors already suffered at enrollment.

16. Patients who currently participating in an interventional clinical study or receiving another study drug within 4 weeks prior to initial administration. Received Chinese herbal or proprietary medicines with antitumor effects within 2 weeks prior to initial administration.
17. Patients with any active autoimmune disease or a history of autoimmune disease (including but not limited to autoimmune hepatitis, interstitial lung disease, uveitis, enteritis, hepatitis, hypophysitis, vasculitis, nephritis, hyperthyroidism, vitiligo; asthma with complete remission in childhood and without any intervention in adulthood could be included to the study; patients with asthma requiring medical intervention with bronchodilators were excluded to the study).
18. Corticosteroids were required within 14 days prior to administration of the study drug (＞10 mg/day equivalent dose of prednisone) or other immunosuppressive drugs for systemic therapy.
19. Patients with congenital or acquired immune deficiency (e.g. HIV positive).
20. Patients with active tuberculosis (TB). Patients suspected of having active TB should be excluded by examination of chest X-rays, sputum, together with clinical signs and symptoms.
21. Patients with severe infection occurring within 4 weeks of initial administration, including, but not limited to, complications requiring hospitalization, sepsis, or severe pneumonia.
22. Patients with HBV DNA＞2000 IU/ml (or 10^4 copies/ml) or HCV RNA＞10^3 copies/ml or HBsAg positive and anti-HCV antibody positive patients;
23. Patients with symptomatic central nervous system metastases.
24. Severe, uncontrolled angiocardiopathy (heart failure > class II NYHA, unstable angina, myocardial infarction within past 1 year, supraventricular or ventricular arrhythmia which need medical intervention, or QT interval male ≥ 450 ms, female ≥ 470 ms.).

25. Live vaccine was administered within 30 days prior to initial administration or was planned for the study period.
26. Pregnant or nursing.
27. Patients with any condition that, in the investigators opinion, could lead to a ri sk of receiving the study drug or would interfere with the evaluation of the study drug or with the patients safety or study results interpretation.

4.4 Criteria for Discontinuation

The patients treatment should be discontinued if any of the following occurs :

1. ICF was withdrawn by the patient.
2. Patients with objective PD (imaging or clinical assessment to determine progression).
3. Patients who have accumulated 2 years of medication (no imaging progression).
4. Patients who develop serious adverse events (SAE) and the investigators confirm that the treatment should be terminated.
5. Patients who develop comorbidities that the investigator consider that the treatment should be terminated.
6. The investigators consider that the best option for patients is to stop treatment.
7. In the investigators opinion, patients intentionally or unintentionally fail to comply with the studys regulations and termination of the treatment study is necessary.
8. If a patient is confirmed to have imaging progression, the investigator determines that it will benefit to continue using the study drug; if the patient is fully informed and signs the ICF to continue using the drug after PD, the patient can continue using the study drug until imaging progression occurs again or no longer benefits from the treatment. Patients who continue to take medication after PD will be visited periodically and evaluated for efficacy according to the visit procedure specified in the trial protocol. Patients who continue to take medication after PD must be fully informed of the possible alternative treatment and the potential risks of continuing medication.

4.5 Criteria for Early Withdrawal

Patients have the right to withdraw from the study at any time for any reason. The investigator also reserves the right to withdraw subjects from the study in the event of an episodic illness, AE, protocol violation, administrative or other reasons. Once subjects decide to withdraw from the study, every effort should be made to complete and report the treatment evaluation endpoint as fully as possible. The subject should be asked if he/she can be contacted for further information. The results of this discussion should be documented in the patient history and case report form (CRF). The investigator should contact the patient or his/her relatives by telephone or in person to fully substantiate the reason for withdrawal as far as possible. At the time of withdrawal, a complete final evaluation should be made by explaining why the subject withdrew from the study. If the reason for withdrawal is an adverse event, the major specific event should be recorded in the CRF. An excessively high drop-out rate could leave the study in an unexplained state, so unnecessary subject drop-outs should be avoided. If the subject decides to withdraw from the study, every effort should be made to complete and report the observations as completely as possible prior to withdrawal.

4.6 Concomitant Drugs and Treatment

Concomitant treatment included any medications (e.g., prescription drugs, OTC drugs, herbal, nutritional supplements) used by the patient during the 7 days prior to the administration of the study drug through the visit at the end of treatment. All concomitant drugs should be reported to the investigator and documented in concomitant drugs for CRF.

The following drugs are prohibited during the study:

a) Any other study drug.

b) Palliative radiotherapy for target lesions, any anticancer therapy, antibot-based therapy, retinoid therapy, hormone therapy, nitrosourea therapy, mitomycin C therapy, small molecule tyrosine kinase inhibitors therapy, or proprietary Chinese medicines with antitumor activity.

c) Immunosuppressive drugs include, but are not limited to, systemic glucocorticoids (doses greater than 10 mg per day of prednisone or equivalent),

methotrexate, azathioprine, and TNF-α antagonists. The use of immunosuppressive therapy to treat study drug associated AE or in subjects allergic to contrast agents is acceptable. In addition, inhaled, topical, and intranasal glucocorticoids are permitted. Glucocorticoids are permitted as a prophylactic treatment for hypersensitivity (e.g., before CT examination). Short-term use of glucocorticoids for the treatment of underlying diseases or comorbidities may be permitted after discussion with the medical examiner.

d) Live vaccine (during the study period to 120 days after the last administration).

e) Concomitant use of dietary supplements, non-investigator prescribed medications and alternative/complementary therapies are not encouraged but are not prohibited and should be discussed with the medical examiner.

f) Prior medication (primary prophylaxis) to prevent an infusion-related reaction is not permitted before the first infusion of SHR-1210. The use of any planned prophylaxis (secondary prophylaxis) in subsequent SHR-1210 infusion is permitted after an infusion related reaction has occurred.

5.0 Evaluation Schedule

The treatment period is 21 days per cycle. The schedule of assessment from screening to treatment is shown in Table 1. During treatment, the investigator may increase the number of laboratory tests or other laboratory tests required by the patients condition.

1. Screening and Baseline Evaluation (within 0-14 days):
    a. Eligibility for the study was determined and signed ICF
    b. Medical history review
    c. The present medications and treatment
    d. Body examinations, include height, weight and vital signs
    e. Physical examination of head and neck region, include nasopharynx and cervical lymph nodes
    f. Physical examination of the nervous system
    g. ECOG performance status score
    h. Nasal endoscopy and lesion biopsy
    i. Enhanced MR of neck and nasopharynx

j. Systematic PET/CT
k. ECG
l. UCG
m. Test about Hepatitis B, Hepatitis C and HIV
n. Blood biochemistry (liver function, kidney function, electrolytes, etc.)
o. Blood routine (white blood cells, neutrophils, platelets, hemoglobin)
p. Urine routine
q. Thyroid function (TSH, FT3, FT4)
r. Pregnancy test
s. Blood coagulation function
t. Quantitative detection of plasma EBV-DNA
u. Fresh tumor tissue from nasopharyngeal biopsy collected before treatment
v. Biomarkers in peripheral blood (including but not limited to TMB assessed by genome sequencing)

2. During Treatment
   a. Tumor imaging evaluation schedule is shown in below part “Imaging Evaluation”.
   b. Physical examination
   c. Vital signs
   d. ECOG performance status score
   e. Laboratory tests: ECG, blood routine, blood biochemistry, urine routine, thyroid function, blood coagulation function and Quantitative detection of HBsAg (for HBsAg abnormality at baseline) are required on Day 1 per treatment cycle. Blood routine, blood biochemistry, urine routine are required on Day 8 and Day 15 per treatment cycle.
   f. Biomarker test: plasma EBV DNA test and peripheral blood samples were collected before each treatment cycle.
   g. Administration records: administration records of SHR-1210 and apatinib. In order to improve patient compliance and ensure that apatinib is taken after breakfast every day, only SHR-1210 can be given on the day of the first cycle

Day 1, and apatinib can be taken orally after breakfast in Day 2 for continuous 3 weeks as a treatment cycle. Acute allergic reactions should be closely observed within 24 hours after the initial administration of SHR-1210, and should be treated according to hospitals medical practice and relevant guidelines if they occur.

h. Use of concomitant drugs.

i. Acute toxicity (according to NCI-CTCAE 5.0), including hematological toxicity, dermal toxicity, gastrointestinal reaction, liver and kidney function impairment, neurotoxicity, etc..

j. Blood pressure monitoring during treatment was completed by the patient and recorded in the patient diary card.

3. Imaging Evaluation

In period of treatment imaging evaluation should be performed under the same conditions as baseline examination (thickness of scan, contrast agent, etc.). Every 6 weeks (±7 days) (relative to the date of first SHR-1210 plus apatinib administration), the tumor was evaluated with enhanced CT or MRI of the lesion and the preferred imaging method of distant metastasis until the patient had confirmed PD, withdrawn ICF, died, or until the study was closed, whichever occurred first. After 12 cycles of treatment, every 12 weeks (±7 days) (relative to the date of first SHR-1210 plus apatinib administration), the tumor was evaluated with enhanced CT or MRI of the lesion and the preferred imaging method of distant metastasis until the patient had confirmed PD, withdrawn ICF, died, or until the study was closed, whichever occurred first. If a new lesion is suspected, it can be examined timely. The first time of PR or CR must be confirmed after 4 weeks ±7 days. If PD first appears according to RECIST (version 1.1), imaging evaluation should be performed 4-6 weeks later to confirm it (except those with rapid progression and significant clinical progression). If the patients clinical condition is unstable after the initial evaluation of radiographic progress, the investigator may directly determine that the subject terminate treatment without confirmation 4-6 weeks later. Unplanned imaging examination may be performed when disease progression is suspected, such as worsening of symptoms. If no imaging

examination is performed within 4 weeks before the end of treatment, an imaging examination should be performed at the end of treatment or the end of study to evaluate the efficacy. For patients with non-radiographic evidence of progression (intolerable, other situation), tumor evaluation is performed every 3 months until disease progression, death, or initiation of other anti-tumor therapy.

4. Safety Visit

Performed at 30 days after treatment termination or completion (30 days ±3d after last administration). It included vital signs, physical examination, ECOG score, blood routine, blood biochemistry, urine routine, adverse events, and concomitant medications.

5. Survival follow-up

Subjects will be contacted every 3 months after the safety visit (telephone visits are available) to obtain as much information as possible about survival and about any subsequent systemic anti-tumor treatment and disease progression information (for subjects with no radiographic progression of disease). Long-term follow-up will continue until death or the end of the study.

Table 1: The step of study from screening to treatment

	During treatment until disease progression													End of treatment/exit treatment
**Therapy cycle**		**1**			**2**			**3**			**Subsequent treatment if applicable**			
**Days**	**-14~0**	**1**	**8**	**15**	**22**	**29**	**36**	**42**	**49**	**56**	**D1**	**D8**	**D15**	
Radiographic evaluation of tumors by RECIST 1.1 (including use of appropriate imaging techniques) [a]	×							×						×
Informed consent form	×													
Medical history, treatment history, and medication history[b]	×													
Physical examination	×	×			×			×			×			×
Vital sign[c]	×	×			×			×			×			×
ECOG performance status [d]	×	×			×			×			×			×
ECG	×	×			×			×			×			
UCG	×				×									
Pregnancy test	×				×									
Blood coagulation test	×	×			×			×			×			
Blood routine, blood biochemistry, urine routine	×	×	×	×	×	×	×	×	×	×	×	×	×	×
Thyroid function：TSH, FT4, FT3	×	×			×			×			×			
Test about Hepatitis B, Hepatitis C and HIV [e]	×	**Check regularly if there is any abnormality**												
Biomarkers in peripheral blood	×	×			×			×			×			×
Quantitative detection of plasma EBV-DNA	×	×			×			×			×			×
Archived tumor tissue samples/fresh tissue biopsy[f]	×													
Study drug administration records		×			×			×			×			
concomitant medication/concomitant therapy[g]	×	×	×	×	×	×	×	×	×	×	×	×	×	×
Evaluation of AE/SAE	×	×	×	×	×	×	×	×	×	×	×	×	×	×
Study temination record		**Keep records and report as required**												
Study End Record														×

Abbreviations:

AE: Adverse Event; **ECG:** Electrocardiograph; **ECOG:** Eastern Cooperative Oncology Group; **HIV:** Human Immunodeficiency Virus; **RECIST:** Response Evaluation Criteria In Solid Tumors; **SAE:** Serious Adverse Events; **T3:** Triiodothyronine; **T4:** Tetraiodothyronine; **TSH:** Thyroid Stimulating Hormone.

Annotation:

1. Assessment at baseline included nasopharynx and neck MRI and whole-body PET-CT. If there is evidence of metastasis to any other lesions at the time of screening, CT/MRI scans of these lesions should be performed based on the clinical signs and symptoms of the individual subject and followed throughout the study. All known lesions must be recorded during screening and repeated at each subsequent tumor evaluation. In the opinion of the investigators, other methods may be used to assess measurable lesions according to RECIST1.1. To evaluate efficacy, baseline and post-treatment testing methods should be consistent as it is easy to compare.
2. Past medical history: any past medical history, drug allergy, smoking history and alcohol consumption history which are inappropriate to this indication, starting before ICF collection and signing. Previous tumor history and treatment history: Previous tumor history includes pathological diagnosis, histological grade, clinical stage, time of initial diagnosis, time of recurrence, etc. Previous treatment history included previous surgical history, chemotherapy history, targeted therapy history, immunotherapy history and radiotherapy history;
3. Vital signs include sitting blood pressure, pulse, respiration, and body temperature.
4. ECOG performance status is an indicator of patient's general health status and tolerance to treatment by the physical strength of the patient. There are six levels from 0 to 5. The score was low and the patient was in good health. It is generally considered that patients with a score above 3 are not suitable for chemotherapy.
5. Hepatitis B virus tests include hepatitis B surface antigen (HBsAg), hepatitis B surface antibody (HBsAb) (HBV DNA test if required), hepatitis C core antibody (HBcAb) (if antibody test is positive, Hepatitis C RNA qualitative detection is required) and HIV-Ab.
6. For all subjects, fresh tumor biopsy will not be required at screening, if archived tumor biopsy samples are available, archived tumor biopsy samples will be

provided at baseline or progressed after treatment. If archived tumor tissue samples are not suitable for use, fresh tumor biopsies will be obtained at baseline before subjects receive their first administration.

7. Concomitant medication/concomitant treatment: records are made of medications and concomitant treatment for diseases other than this indication within 30 days prior to screening and during treatment.

Table 2: Safety and survival visit

Months (after treatment/withdrawal treatment)	Safety visit	Post-treatment monitoring			
	**1**	**4**	**7**	**10**	Every three months
AE associated with drugs[a]	×	Information about AE associalte the drug will be collected until resolved or stabilized, regardless of how long it has been since the last administration of any study drug.			
Survival evaluation[b]		×	×	×	×
Late stage of antitumor therapy		Information will be collected about any subsequent antitumor therapy(late stage of study administration), including all therapeutic drug names, usage, and treatment start and end dates.			

Abbreviations:

AE: Adverse Event;

Annotation:

a. Collect only drug-related AE, that is, AE that are thought to be related to the study administration (SHR-1210, apatinib).

b. Patients survival will be followed every 12 weeks until the end of the study. It started one month after the safety visit.

5.1 Screening Examination and Qualifying Screening Forms

All patients must be provided written ICF prior to any specific study evaluation and procedure. Screening tests should be performed within 14 days prior to the start of treatment. If screening is done within the baseline window (-7 to 0 days), no additional baseline visit is required. Patients who sign ICF and meet all eligibility criteria will be enrolled in the study.

5.2 Procedures for the Inclusion of Qualified Subjects

All eligibility criteria were evaluated for each patient in parallel, and baseline evaluations were guaranteed within 14 days prior to enrollment. If the patient meets all enrollment criteria, the patient will receive an official study number and can be enrolled in the study.

5.3 Clinical Evaluation and Procedures

5.3.1 Tumor Remission Evaluation

Patients with measurable lesions could be enrolled in this study. Tumor evaluation is required according to RECIST criteria (version 1.1) (see Appendix 1). Only measurable lesions can be used as target lesions, and when more than one measurable lesion is present at baseline, all lesions representing all affected organs (a maximum of 5 lesions in total, and 2 lesions per organ) should be identified as target lesion. For measurable lesions, CT scan or MRI examination should accurately measure at least one dimension of the tumor lesion (record the maximum diameter of the measurement plane), and the minimum size of the target lesion should be≥10mm (scan layer thickness should not exceed 5mm). Malignant lymph nodes: when evaluated by CT scan (CT scan thickness is recommended not to exceed 5mm), the short diameter of lymph nodes must exceed 15mm to be considered pathologically enlarged and measurable. For each patient, the same assessment method must be used throughout the study, and if more than one method is used during the study, the most accurate method should be selected for data recording according to RECIST criteria (version 1.1).

5.3.2 Physical Status

The PS score will be based on the ECOG PS score (Appendix 2). Evaluations will be made at baseline and at planned visits every 21 days thereafter. If possible, it is

recommended that the PS of the same patient be assessed by an investigator throughout the study.

5.3.3 Clinical Safety Evaluation

In this study, the clinical safety of the treatment will be evaluated by using NCI-CTC AE (Version 5.0). Subjects should be assessed for adverse events at each clinical visit and whenever necessary throughout the study. Complete medical history, physical examination, vital signs, and baseline information should be recorded during screening.

5.4 Laboratory Evaluation

Hematologic analyses and blood biochemical are required as part of routine safety evaluation. Details are as follows:

1. Blood routine: hemoglobin, white blood cell count, neutrophil count, platelet count.
2. Blood biochemistry: ALT, AST, total bilirubin, alkaline phosphatase, creatinine, urea nitrogen, albumin, electrolytes (sodium, potassium, chlorine, calcium).
3. Urine routine will be used to check the presence of protein, glucose and blood in the urine.
4. Blood routine, blood biochemistry, and urine routine are scheduled as follows: within 14 days at screening, within 7 days before treatment initiation at baseline, D1, D8, D15 at each subsequent treatment cycle, and at study exit. Blood routine and blood biochemistry can be performed more frequently if clinical indications require.

5.5 Clinical Treatment After the Study

Clinical treatment of patients after completion of this study will be based on the judgment of the investigator.

5.6 Subsequent Antitumor Therapy

After investigators' initial evaluation of PD as defined by RECIST (Version 1.1) criteria, subjects will be allowed to continue study therapy if they are evaluated as clinically beneficial and tolerate to the study drug. After evidence of further PD, subjects must terminate study treatment. After radiographic confirmation of PD,

subjects may continue to receive SHR-1210 plus apatinib if all of the following criteria are met:

1. The subject showed no progression-related clinical symptoms or signs, and the investigator assessed that the subject would still be clinically stable on SHR-1210 plus apatinib.
2. Subjects could tolerate SHR-1210 plus apatinib treatment.
3. ECOG score is stable.
4. Do not delay the management of serious complications (such as central nervous system metastasis) that require urgent intervention.
5. Before proceeding with SHR-1210 plus apatinib, the investigator needs to clarify all foreseeable risks or discomfort and other treatment options.

5.7 Follow-up Time

The last subject needs to complete at least 24 weeks of follow-up (more than 24 weeks between data cutoff date and initial administration date).

6.0 Information on The Investigational Drugs

Jiangsu Hengrui Pharmaceutical Co., Ltd. will provide the investigator with or through designated distribution centers sufficient quantities of the study drug.

6.1 Dose and Administration Schedule

SHR-1210,200 mg is administered every three weeks and every three weeks is one treatment cycle. The drug is administered intravenously at 60 minutes (±10 minutes), and may be extended up to 120 minutes (±15 minutes) for subjects who cannot tolerate a 60 minute-infusion. Until toxicity is unacceptable, or PD, or ICF is withdrawn, or the investigator determines that the patient needs to terminate treatment, or the maximum treatment period of 2 years is reached. Generally, the delay of drug administration is no more than 3 days. In special cases, if the delay is no more than 7 days, it is recommended to recalculate the time window based on the actual time of drug administration, and the time window for subsequent drug administration is still 3 days. If the delay is more than 7 days, it is recommended not to give the drug this time and to continue drug administration next time according to the scheduled date. Delay drug administration for

up to 12 weeks since last dosing, or terminate treatment.

Apatinib, 250 mg once daily, orally administered half an hour after breakfast, is administered every 3 weeks as a cycle of treatment until toxicity is unacceptable, PD occurs, ICF is withdrawn, the investigator determines the patient needed to withdraw from treatment, or a maximum of 2 years of treatment is reached. Apatinib dose adjustment and administration delay are allowed for up to 4 weeks from last dosing, otherwise treatment will be terminated.

6.2 Preparation and Management of Drugs

1. Study drug 1: Camrelizumab SHR-1210 for injection

   Manufacturer: Suzhou Shengdiya Biological Medicine Co., LTD

   Dosage form: lyophilized powder

   Specification: 200 mg, packed in 20 mL vial.

   Usage: intravenous drip

   Storage conditions: sealed, away from light, stored in 2-8°C medical refrigerator, not frozen.

2. Study drug 2: Apatinib mesylate tablet

   Manufacturer: Jiangsu Hengrui Pharmaceutical Co., LTD

   Dosage form: tablet

   Specifications: 250 mg/tablet

   Usage: Oral

   Storage conditions: shading, sealed, stored below 25°C.

6.3 Compliance Evaluation

The investigator administer all study drugs (SHR-1210, apatinib). Appropriate records of study drug receipt (e.g., drug receipt record) and disposal (e.g., drug release log) must be maintained. Drug counts and patient compliance are evaluated by maintaining appropriate records of drug delivery and return.

Accurate records of the study drugs provided by the sponsor must be maintained. These records must contain the following information:

1. Records of drug shipments receive from the sponsor (received date and quantity).
2. Disposition of unused study drugs not issued to patients.

Updated SHR-1210 release records must be maintained and should contain the following information:

1. Identifying information of patients who receive the study drug.
2. The date and quantity of the study drug given to the patient.

Updated apatinib release and return records must be maintained and should contain the following information:

1. Identifying information of the patients who receive the study drug.
2. The date and quantity of the study drug given to patients.
3. The date and quantity of the study drug returned by patients.

7.0 Safety

7.1 Adverse Events and Abnormal Laboratory Tests

7.1.1 Clinical Adverse Events

Any adverse medical event occurring between the time the subject signs the ICF and is enrolled in the study and the last visit, regardless of whether it is causally related to the study drug, will be considered an AE.

AE include the following:

1. All suspected adverse drug reactions.
2. All reactions due to drug overdose, abuse, withdrawal, allergy or toxicity.
3. Apparently unrelated diseases, including exacerbations of pre-existing diseases.
4. Injury or accident, record the result of this accident in remarks.
5. Abnormalities detected by physiological examination or physical examination that require clinical treatment or further examination.

7.1.1.1 Strength Grade of Adverse Event

The study physician should use concise medical terminology to report all AE observed directly by the physician or reported spontaneously by the subject. In addition, patients should be asked about AE at each visit at the beginning of treatment, and truthfully fill in the AE record form to record the occurrence time, severity, duration, measures taken and outcomes of AE. AE should be recorded in the CRF adverse event table. When filling out the CRF adverse event table, researchers will use mild, moderate,

and severe to describe the intensity of AE. As a unified standard, AE intensity is graded as follows:

1. Mild: does not affect normal function of the subject.
2. Moderate: the subjects normal function is affected to some extent.
3. Severe: the normal function of the subject is significantly affected.

Care should be taken to distinguish the severity and intensity of adverse events. Severe is used to describe strength, not necessarily SAE. A headache, for example, may be severe in intensity but not SAE unless it meets SAE criteria.

7.1.1.2 Serious Adverse Events

AE are classified as serious adverse events (SAE) when they meet one or more of the following criteria:

1. Resulting in death.
2. Life-threatening.
3. Requiring hospitalization or prolonged hospitalization.
4. Causes persistent or severe disability or dysfunction.
5. Congenital malformations or birth defects.

A medical event that does not result in death, life-threatening or hospitalization shall also be considered SAE if it is determined by proper medical judgment that it may cause harm to the patient or subject or that medication or surgical treatment is required to prevent such occurrence.

When SAE occur in subjects during clinical trial, clinicians will report the types and severity of SAE to the head of the center within 30 minutes. Report serious SAE to director of GCP Office, director of medical service, director of the hospital in charge and director of ethics committee of Sun Yat-sen University Cancer Center within 24 hours after receiving the report, fill in the corresponding form, sign and date of signature.

7.1.2 Relationship Between Drugs and Events

After the end of the study, the relationship between adverse reactions and the drugs under study will be evaluated by five grades: drug-related, very likely drug-related, likely drug-related, likely drug-unrelated and drug-unrelated. The first three are counted as adverse reactions, and the incidence of adverse reactions is counted.

The investigator should evaluate the possible association between AE and the study drug or combination drug by referring to the following 5-level classification criteria:

1. Relevant: reactions occur in a reasonable time sequence after administration, and reactions conform to the known AE type of the suspected drug. The reaction improves after discontinue administration and recurs after repeated administration.
2. Possible relevant: reactions occur in a reasonable time sequence after administration, and reactions conform to the known AE type of the suspected drug. The subjects clinical status or other treatment may also contribute to this reaction.
3. Possible irrelevant: reactions do not appear in a reasonable time sequence after administration, and reactions do not conform to the known AE type of the suspected drug. The subjects clinical status or other treatment may also contribute to this reaction.
4. Irrelevant: reactions occur that do not conform to a reasonable post-administration time sequence, and reactions that conform to the known AE type of the non-study drug. The reaction may be due to the subjects clinical status or other treatment. Reactions are eliminated with improvement of the disease status or cessation of other treatments, and reactions occurs with repeated use of other treatments.
5. Undeterminable: The occurrence of reactions has no significant relationship to the time sequence after administration. Reaction are similar to the known type of AE of this drug and may be caused by other drugs used at the same time.

7.1.3 Follow-up of Adverse Events

All AE should be followed up to 30 ±3 days after the last administration of the study therapy. Ongoing AE should be followed up as described below:

1. AEs related to study therapy: follow up until:
   a. Remission or improvement to baseline level.
   b. The relationship was reassessed and deemed irrelevant.
   c. Death.
   d. Start a new antitumor treatment.
   e. The researchers confirm that no further improvement is expected.
   f. The sponsor no longer collects clinical or safety data, or the database is

ultimately closed.

2. Severe or life-threatening AEs not related to study treatment: follow up until:
   a. Remission or improvement to baseline level.
   b. Severity improved to grade 2.
   c. Death.
   d. Start a new antitumor treatment.
   e. The researchers confirm that no further improvement is expected.
   f. The sponsor no longer collects clinical or safety data, or the database is ultimately closed.
3. Grade 1 or 2 AEs not related to study treatment: follow up until:
   a. The researchers confirm that no further improvement is expected.
   b. The sponsor no longer collects clinical or safety data, or the database is ultimately closed.

The final result of each AE must be recorded on CRF.

7.1.4 Abnormal Laboratory Examination

All laboratory examination results should be recorded on the CRF laboratory examination results page or on the electronic laboratory report provided directly by the central laboratory (whichever is appropriate).

Any unexpected laboratory examination result that is clinically significant for the treatment of one or more of the following conditions should be recorded as a diagnosis on the AE page of the CRF:

1. Accompanies with clinical symptoms.
2. Causes study drug to change (e.g., dose adjustment, suspension or permanent cessation).
3. Require a change in combination therapy (e.g., addition, suspension, termination, or other change in combination medication, therapy or treatment).

The above conditions apply to laboratory test results for safety and efficacy of any protocol and non-protocol description after initial administration if they are outside the laboratory reference range and meet the criteria for clinical significance.

The above conditions do not apply to any laboratory test results that fall outside the

laboratory reference range but do not meet the criteria of clinical significance (to be analyzed and reported as laboratory abnormalities). For example, AEs that are explicitly defined as exempt types in the schema and those resulting from laboratory tests that have been reported for AE.

Please note: Any abnormal laboratory results that meet SAE standards are reported as SAE and recorded as AE in the CRF.

7.1.4.1 Follow-up of Abnormal Laboratory Results

Abnormal laboratory results that are medically significant but unclear should be redone and followed up until they return to normal ranges, baseline values, and/or adequate explanations for the outliers can be found. If there is a clear explanation, it should be recorded on the CRF notes page.

7.2 Security Parameter Processing

7.2.1 Adverse Event Reporting

All AE (related and unrelated) occurring during the study period up to 30 days after the last administration of any study drug must be reported.

7.2.2 Serious Adverse Event Report (immediately reported)

When SAE occur in subjects during clinical trial, clinicians will report the types and severity of SAE to the head of the center within 30 minutes. Report serious SAE to director of GCP Office, director of medical service, director of the hospital in charge and director of ethics committee of Sun Yat-sen University Cancer Center within 24 hours after receiving the report, fill in the corresponding form, sign and date of signature.

7.2.3 Pregnancy

If a woman becomes pregnant during the study, she must be advised to stop taking the drug and notify the investigator immediately. Pregnancies that occurred six months after completion of the study also had to be reported to the researchers. The researcher should report all pregnancies to the sponsor and Jiangsu Hengrui Pharmaceutical Co., LTD within 24 hours. Researchers should advise patients and discuss the risks of continuing the pregnancy and possible effects on the fetus. Patients should continue to be monitored until the end of pregnancy. Pregnancy of a male subjects partner during

the study period should also be reported to the investigator and sponsor. As noted above, patient's partner should also be counseled and followed up.

7.3 Dose Adjustment for Toxicity

7.3.1 Dose Adjustment of SHR-1210

Dose adjustment of SHR-1210 (AE occurring during treatment will be graded according to NCI CTCAE version 5.0, and treatment of SHR-1210 was adjusted based on the grading to manage potential irAE.).

1. Dose adjustment is not required for grade 1 irAE.
2. For grade 2 irAE, SHR-1210 treatment will be discontinued until grade 2 regresses to grade 1:
   a. If toxicity worsens, treat it as grade 3 or 4.
   b. If toxicity improves to baseline, treatment is performed at the next scheduled treatment visit.
   c. SHR-1210 will be discontinued permanently if grade 2 irAE does not regraded to grade 1 or baseline within 8 weeks.
3. For grade 3 irAE, SHR-1210 treatment can be permanently discontinued depending on individual toxicity.
4. For grade 4 irAE, ShR-1210 therapy must be permanently discontinued.

The longest time that SHR-1210 allows to pause is 8 weeks at a time. If no recovery is achieved within 2 weeks, subjects will permanently discontinue SHR-1210 and enter the follow-up phase. The exception is that SHR-1210 is suspended for more than 8 weeks due to glucocorticoid reduction in the treatment of irAE or for AE that may be unrelated or unrelated to SHR-1210. Guidelines for the treatment adjustment and toxicity management of SHR-1210 for immune-related AE are shown in Table 3. For irAE not covered in Table 3 or for specific cases of specific toxicity, refer to ASCO guidelines for treatment.

Table 3: Therapeutic adjustments and recommended toxicity management guidelines for SHR-1210 on immune-related adverse events

Reactive capillary hyperplasia		
The severity of reactive capillary hyperplasia (ESMO v2017)	Treatment	Follow-up
**Grade 1**<br>Single or multiple nodules, maximum diameter<10 mm, with or without ruptured bleeding	Continue treatment with SHR-1210<br>Symptomatic treatment	For bleeding patients, local antibiotic ointment treatment to prevent infection.
**Grade 2**<br>Single or multiple nodules, maximum diameter>10 mm, with or without ruptured bleeding	Continue treatment with SHR-1210	Consider local treatment, such as laser or surgical excision, depending on the condition.<br>For bleeding patients, local antibiotic ointment treatment to prevent infection.
**Grade 3**<br>Multiple nodules with infection.	Suspend SHR-1210 treatment<br>Symptomatic treatment	If improved to grade 1: restart SHR-1210 treatment Consider local treatment, such as laser or surgical excision, depending on the condition.<br>Patients with local infection should be treated with anti-infective therapy.
Gastrointestinal tract irAE		
Severity of diarrhea/colitis (NCI-CTCAE v5.0)	Treatment	Follow-up
**Grade 1**	Continue treatment with SHR-1210	Monitor closely whether symptoms are getting worse.

Diarrhea: increased stool frequency < 4 times per day compared to baseline<br>Colitis: asymptomatic	Symptomatic treatment (e.g., loperamide)	Instruct subjects to report deterioration immediately.<br>If it worsens, treat as grade 2 or 3-4.
**Grade 2**<br>Diarrhea: increased stool frequency by 4-6 times per day compared to baseline; ADL is not disturbed by intravenous fluid replacement no longer than 24 hours.<br>Colitis: abdominal pain; hematochezia.	Delay SHR-1210 treatment Symptomatic treatment	If improved to grade 1: restart SHR-1210 therapy (if glucocorticoid has been given, glucocorticoid dose should be gradually reduced before restarting therapy).<br>If it continues 5 to 7 days or relapse: 0.5 to 1.0 mg/kg/ day of methylprednisolone or equivalent medicine, when symptoms improve to grade 1, gradually reduce glucocorticoid dose for at least 1 month, consider prophylactic antibiotics to prevent opportunistic infections, and then restart SHR-1210 therapy as scheduled.<br>If symptoms worsen or persist after orally giving glucocorticoids for 3 to 5 days: treat as grade 3 to 4.
**Grade 3-4**<br>Diarrhea (grade 3): increased stool frequency by ≥7 times per day compared to baseline fecal incontinence, intravenous infusion longer than 24 hours with ADL interference.<br>Colitis (grade 3): severe abdominal pain, medical intervention, peritoneal signs.	Grade 3：delay SHR-1210 treatment.<br>Grade 4: permanently discontinue SHR-1210 therapy.<br>Methylprednisolone or its equivalent is given intravenously at 1.0 to 2.0 mg/kg/ day.<br>Increase prophylactic antibiotics for the prevention of opportunistic infections.<br>Consider endoscopy of the lower abdomen.	If it improves:<br>Glucocorticoid therapy will be continued until regression to grade 1 and then tapered for at least 1 month.<br>If it continues longer than 3 to 5 days, or recurrence after improvement:<br>Add 5 mg/kg infliximab (if no contraindications).<br>Note: Infliximab should not be used in cases of perforation or sepsis.

level 4: life-threatening, perforation.		
Skin irAE		
Severity of rash<br>(NCI-CTCAE v5.0)	Treatment	Follow-up
**Grade 1-2**<br>Covering ≤ 30% body surface area (BSA)	Symptomatic treatment (e.g., antihistamines, topical glucocorticoids)<br>Continue treatment with SHR-1210	If it continues longer than 1 to 2 weeks or recurrence:<br>Consider a skin biopsy<br>Delay SHR-1210 treatment<br>Consider intravenously infusing methylprednisolone 0.5 to 1.0 mg/kg/ day or its equivalent. If there is improvement, gradually reduce glucocorticoid dose for at least 1 month, consider prophylactic antibiotics to prevent opportunistic infections, and restart SHR-1210 therapy.<br>If it worsens, treat it as grade 3 to 4.
**Grade 3-4**<br>Covering > 30% BSA	Grade 3: delay SHR-1210 treatment.<br>Grade 4：permanently discontinue SHR-1210 therapy.<br>Consider a skin biopsy<br>Dermatological consultation<br>Intravenously infuse methylprednisolone or its equivalent at 1.0 to 2.0 mg/kg/ day.	If the grade 3 rash does not improve to grade 1 or baseline within 30 days after a temporary delay of treatment of SHR-1210, SHR-1210 treatment will be permanently discontinued.<br>If improved to Level 1:<br>Gradually reduce glucocorticoid dose for at least 1 month and increase prophylactic antibiotics to prevent opportunistic infection.<br>Restart SHR-1210 treatment (for grade 3 events improved to grade 1).

Lung irAE		
Severity of Pneumonitis (NCI-CTCAE v5.0)	Treatment	Follow-up
**Grade 1**<br>Asymptomatic; clinical or diagnostic observations only	Continue treatment with SHR-1210;<br>Consider consultation for pulmonary infectious diseases;<br>Monitor symptoms and oxygen saturation every 2-3 days (using a personal pulse oximeter); Weekly clinic visit	Imaging examination should be performed at least once every 3 weeks.<br><br>If worsen, treat it as grade 2 or 3-4.
**Grade 2**<br>Symptomatic; medical intervention indicated;<br>limiting instrumental ADL	Suspend SHR-1210 treatment;<br>Respiratory and infectious disease counselling;<br>Bronchoscopy and bronchoalveolar lavage;<br>Consider biopsy for atypical lesions;<br>Monitor symptoms daily and consider hospitalization;<br>Methylprednisolone 1.0 mg/kg/ day intravenously infusion or its equivalent	Imaging examinations are performed every 1 to 3 days<br>If improves:<br>When symptoms return to near baseline levels, reduce glucocorticoid dose gradually for at least 1 month, then restart SHR-1210 therapy and consider prophylactic antibiotics.<br>If there is no improvement or even deterioration after 2 weeks:<br>Treat it as grade 3 to 4.
**Grade 3-4**<br>Newly appeared severe symptoms;<br>Hypoxia symptoms or worsening hypoxia symptoms; Life-threatening	Permanently discontinue SHR-1210 therapy;<br>Hospitalization: ICU care is considered;<br>Counselling for lung and infectious	If improves to baseline level:<br>Reduce glucocorticoid dose gradually for at least 6 weeks.<br>If there is no improvement or even deterioration after

respiratory	diseases<br>Bronchoscopy and bronchoalveolar lavage; Consider biopsy for atypical lesions;<br>Infuse methylprednisolone (2 to 4 mg/kg/day) or its equivalent intravenously;<br>Increase prophylactic antibiotics for the prevention of opportunistic infections	48 hours:<br>Increase other immunosuppressive agents (e.g., infliximab, cyclophosphamide, IVIG, or mycophenolate mofetil).<br>Consider prophylactic use of antibiotics to prevent pneumocystis pneumonia (PCP) in subjects receiving 20mg methylprednisolone or equivalent dose more than 4 weeks;<br>Calcium and vitamin D supplements should be considered for prolonged steroid use;<br>All subjects receiving steroid therapy for grade 2-4 pneumonia should also be given proton pump inhibitors to prevent gastrointestinal complications;<br>For all subjects considering anti-TNF therapy, t-SPOT should be performed to rule out tuberculosis before initiation of anti-TNF therapy.
Hepatic irAE		
Liver function test parameters increased grade (NCI-CTCAE v5.0)	Treatment	Follow-up
**Grade 1**<br>AST or ALT > ULN to 3.0 × ULN and/or TBil > ULN to 1.5 × ULN	Continue treatment with SHR-1210;	Continue to monitor liver function<br>If worsens:<br>Treat it as grade 2 or 3-4

**Grade 2**<br>AST or ALT > 3.0 ULN to ≤ 5 × ULN or/and TBil > 1.5 ULN to ≤ 3 × ULN	Delay treatment with SHR-1210;<br>Increase monitoring frequency to once every 3 days	If it returns to baseline level:<br>Routine monitoring will be resumed and SHR-1210 therapy will be restarted<br>If the elevation continues 5 to 7 days or worse:<br>0.5 to 1 mg/kg/ day of methylprednisolone or its equivalent, gradually reduce glucocorticoid dose for at least 1 month when LFT returns to grade 1 or baseline, consider prophylactic antibiotics to prevent opportunistic infections, and restart SHR-1210 therapy
**Grade 3-4**<br>AST or ALT > 5 × ULN and/or TBil > 3 × ULN	Terminate treatment with SHR-1210; (If AST/ALT ≤ 8 × ULN and TBil ≤ 5 × ULN, possible delay will be take in SHR-1210 administration)<br>Increase the frequency of monitoring to once every 1-2 days<br>Intravenously infuse 1.0 to 2.0 mg/kg/day of methylprednisolone or its equivalent (in the presence of grade 4 hepatitis, the recommended starting dose is 2.0 mg/kg/day of methylprednisolone or its equivalent); prophylactic antibiotics for the prevention of opportunistic infections; Consult a gastroenterologist;<br>If clinically warranted, consider MRI/CT of the liver, or liver biopsy	If restore to grade 2:<br>Reduce glucocorticoid dose gradually for at least 1 month<br>If no improvement, even deterioration or rebound for more than 3 to 5 days:<br>Add 1g of mycophenolate mofetil , twice a day<br>If there is no response within an additional 3-5 days, other immunosuppressants should be considered according to local guidelines.

Kidney irAE		
Elevated grade of creatinine (NCI-CTCAE v5.0)	Treatment	Follow-up
**Grade 1**<br>Creatine > ULN and > baseline level but ≤ 1.5 × baseline level	Continue treatment with SHR-1210<br>Monitor creatinine levels weekly	If returns to baseline level:<br>Routine creatinine monitoring will be resumed according to the protocol<br>If worsens:<br>Treat it as grade 2-3 or 4
**Grade 2-3**<br>Creatine > 1.5 × baseline level and ≤ 6 × ULN	Delay treatment with SHR-1210<br>Monitor creatinine level once two or three days<br>Intravenously infuse 0.5 to 1.0 mg/kg/day of methylprednisolone or its equivalent<br>Consider biopsy of kidney	If returns to grade 1:<br>Gradually reduce glucocorticoid dose for at least 1 month, consider prophylactic antibiotics to prevent opportunistic infections, restart SR-1210 therapy according to protocol, and initiate routine creatinine monitoring.<br>If the elevation continues 7 days or worse:<br>Treat it as level 4
**Grade 4**<br>Creatine > 6 × ULN	Terminate treatment with SHR-1210<br>Monitor creatinine levels once daily<br>Intravenous infuse 1.0 to 2.0 mg/kg/day Methylprednisolone or its equivalent<br>Consult a nephrologist<br>Consider biopsy of kidney	If returns to grade 1:<br>Gradually reduce glucocorticoid dose for at least 1 month and increase prophylactic antibiotics to prevent opportunistic infections.
Nervous system irAE		

Grade of neurotoxicity (NCI-CTCAE v5.0)	Treatment	Follow-up
**Grade 1**<br>Asymptomatic or mild symptoms; intervention not indicated	Continue treatment with SHR-1210	Continuous monitoring of patients<br>If worsens:<br>Treat it as grade 2 or 3-4
**Grade 2**<br>Moderate symptoms; limiting instrumental ADL	Delay treatment with SHR-1210<br>Treat symptoms according to local guidelines<br>Consider intravenous infuse methylprednisolone 0.5 to 1.0 mg/kg/day or its equivalent	If returns to baseline level:<br>Restart SHR-1210 treatment<br>If worsens:<br>Treat it as grade 3-4
**Grade 3-4**<br>Severe symptoms; limiting selfcare ADL; Life-threatening consequences	Terminate treatment with SHR-1210<br>Get neurological counseling;<br>Treat symptoms according to local guidelines;<br>Consider intravenous infuse methylprednisolone 1.0 to 2.0 mg/kg/day or its equivalent;<br>Increase prophylactic antibiotics for the prevention of opportunistic infections	If returns to grade 2:<br>Gradually reduce glucocorticoid dose for at least 1 month.<br>If it worsens or presents atypical symptoms:<br>Consider IVIG or other immunosuppressive therapies based on local guidelines.

Endocrine irAE		
Grade of endocrine toxicity (NCI-CTCAE v5.0)	Treatment	Follow-up
**Grade 1**<br>(Depending on the type of endocrine disease, refer to NCI CTCAE V5.0 for CTCAE Grade 1 definition)	No dose adjustment	For grade 1 (including asymptomatic patients with elevated TSH):<br>Conduct appropriate endocrine function tests to monitor subject.<br>If TSH < 0.5× LLN or TSH > 2 × ULN, or results consistently exceed the range in the following 2 measurements, FT4 levels will be added in subsequent cycles according to clinical indications, and endocrinology consultation will be considered
**Grade 2**<br>(Depending on the type of endocrine disease, refer to NCI CTCAE V5.0 for CTCAE Grade 2 definition)	Study drug administration can be discontinued until regression to grade 1 or lower.<br>If toxicity worsens, treat it as level 3 or 4.<br>If toxicity improves to baseline, treatment is performed at the next scheduled treatment date.<br>In grade 2 hypothyroidism, immune checkpoint	For grade 2 (including patients with symptomatic endocrine disease):<br>Initiate hormone replacement therapy as needed; Endocrine function is evaluated and pituitary scan is considered according to clinical indications;<br>For subjects with endocrine abnormalities, short-term high-dose glucocorticoid therapy (e.g., intravenously infuse 1 to 2 mg/kg/day methylprednisolone or its equivalent) and hormone replacement therapy (e.g., levothyroxine, hydrocortisone, or sex hormones) are recommended;<br>If there is improvement, glucocorticoid dose should be gradually reduced over 1 month and prophylactic antibiotics should be used to prevent opportunistic infections;<br>For subjects with normal endocrine function (laboratory or MRI scan), repeat laboratory examination/MRI according to clinical indications

	inhibitors therapy may continue together with hormone replacement therapy.	
**Grade 3**<br>(Depending on the type of endocrine disease, refer to NCI CTCAE V5.0 for CTCAE Grade 3 definition)	Study drug administration is discontinued until endocrine disease symptoms are under control.<br>If control is achieved after the next scheduled dose, the study drug administration is restarted.	Initiation of intravenous infusion glucocorticoid therapy (e.g., Methylprednisolone or equivalent) at 1 to 2 mg/kg/day;<br>When isolated hypothyroidism occurs, hormone replacement therapy are used to treat it without interruption or glucocorticoids<br>Use hormone replacement therapy if necessary;<br>For adrenal crisis, severe dehydration, hypotension, or shock: start intravenous infusion glucocorticoid with salt corticoid activity immediately;<br>Endocrinology department consultation;<br>If there is improvement, glucocorticoid dose should be gradually reduced over 1 month and prophylactic antibiotics should be used to prevent opportunistic infections
**Grade 4**<br>(Depending on the type of endocrine disease, refer to NCI CTCAE V5.0 for CTCAE Grade 4 definition)	Permanent discontinuation of study drugs.	The treatment is the same as the grade 3 event above.

7.3.2 Dose Adjustment of Apatinib

Dose Adjustment of Apatinib (AE occurring during treatment is graded according to NCI CTCAE version 5.0, and treatment with apatinib is adjusted based on the grading to manage underlying AE.).

1. Dose adjustment is not required for grade 1~2 AE.
2. For grade 3 AE, apatinib treatment will be suspended until it regrades to grade 1 or lower, and the drug will be continued after the first dose reduction (250mg for 2 days on and 1 day off), and the dose will be lowered again (250mg for 1 day on and 1 day off) if grade 3 AE occurs again.
3. For grade 4 AE, apatinib treatment is immediately and permanently terminated.

After adjustment of apatinib administration during the study period, no callback will be allowed, and apatinib were permanently discontinued if dose interruption exceeded 4 weeks. Guidelines for apatinib treatment adjustment and toxicity management for non-immune-related AE are shown in Table 4. For AE not covered in Table 4 or when specific toxicities exist, refer to ASCO guidelines for treatment.

Table 4: Therapeutic adjustments and recommended toxicity management guidelines for apatinib related adverse events

Hematology AE	
Severity of hematological toxicity (NCI-CTCAE v5.0)	Suggestions of prevention and cure
**Grade 1-2**	No dose adjustment required
**Grade 3**	For the first time, when the adverse events regrade to grade 2 or lower, the original dose can be continued; For the second time, after the adverse events regrade to grade 2 or lower, apatinib can be continued after the first dose reduction; If the dose recurred after reduction, the investigator should evaluate the risks and benefits of continuing apatinib use and determine whether the drug should be terminated permanently.
**Grade 4**	Apatinib is continued after the first dose reduction when the adverse events regrade to grade 2 or lower; If grade 4 toxicity occurs again after dose reduction, the drug will be terminated permanently
Hypertension AE	
Severity of hypertension (NCI-CTCAE v5.0)	Suggestions of prevention and cure
**Grade 1**<br>Systolic BP 120 - 139 mm Hg or diastolic BP 80 - 89 mm Hg;	Monitoring blood pressure closely; Limit salt, quit smoking and alcohol; Continue taking apatinib without dose adjustment
**Grade 2**<br>Systolic BP 140 - 159 mm Hg or diastolic BP 90 - 99 mm Hg;	Monitoring blood pressure closely; Apatinib continues to be taken, generally without dose adjustment. Antihypertensive drugs should be used for treatment and should not be stopped at will

**Grade 3**<br>Systolic BP >=160 mm Hg or diastolic BP >=100 mm Hg;	Stop taking apatinib; For hypertension poorly controlled by single drug, combined drug use should be considered; Seek consultation and treatment from cardiovascular specialists; Monitoring blood pressure closely; If blood pressure is well controlled, apatinib can be continued after initial dose reduction
**Grade 4**<br>Life threatening consequences (e.g., malignant hypertension, transient or permanent neurologic deficit, hypertensive crisis)	Immediately and permanently terminate of apatinib; Consult cardiovascular specialists to actively manage hypertension and closely monitor blood pressure and other vital signs.
Proteinuria AE	
Severity of Proteinuria (NCI-CTCAE v5.0)	Suggestions of prevention and cure
**Grade 1**<br>1+ proteinuria; urinary protein ≥ ULN - < 1.0 g/24 hrs	Continue taking apatinib without dose adjustment
**Grade 2**<br>2+ and 3+ proteinuria; urinary protein 1.0 - < 3.5 g/24 hrs;	Apatinib continues to be taken, generally without dose adjustment; Medical interventions should be considered; 24-hour urine routine and 24-hour urine protein quantification are monitored
**Grade 3**<br>Urinary protein ≥ 3.5 g/24 hrs; 4+ proteinuria;	Stop taking apatinib; Nephrology specialist consultation; Medical intervention; After proteinuria regrade to grade 1 or lower, Apatinib can be taken at a lower dose for the first time; If grade 3 proteinuria still occur after dose reduction, the dose should be reduced again; If proteinuria is persistent and aggravating, apatinib should be terminated permanently.

Palmar-plantar skin reaction AE	
Severity of palmar-plantar skin reaction (NCI-CTCAE v5.0)	Suggestions of prevention and cure
**Grade 1**<br>Minimal skin changes or dermatitis (e.g., erythema, edema, or hyperkeratosis, without pain), but it does not affect daily life.	Apatinib continues to be taken, generally without dose adjustment; When symptoms initially appear, treat with local medication
**Grade 2**<br>Skin changes (e.g., peeling, blisters, bleeding, fissures, edema, or hyperkeratosis) with pain; limiting instrumental ADL	Continue to take apatinib, the dose can be adjusted appropriately; dermal topical administration; Oral B vitamins and celecoxib can be combined with anti-inflammatory or anti-infective drugs
**Grade 3-4**<br>Severe skin changes (e.g., peeling, blisters, bleeding, fissures, edema, or hyperkeratosis) with pain; limiting self care ADL	Stop taking apatinib; Analgesic treatment and dermal topical administration; Combined anti-inflammatory or anti-infective drugs; If symptoms are relieved, dose of apatinib can be lowered for the first time; If grade 3 hand-foot syndrome still occur after dose reduction, the dose should be reduced again; If symptoms are persistent and aggravating, apatinib should be terminated permanently.
Diarrhea AE	
Severity of diarrhea (NCI-CTCAE v5.0)	Suggestions of prevention and cure

**Grade 1**<br>Increase of <4 stools per day over baseline; mild increase in ostomy output compared to baseline	Apatinib continues to be taken, generally without dose adjustment;
**Grade 2**<br>Increase of 4 - 6 stools per day over baseline; moderate increase in ostomy output compared to baseline; limiting instrumental ADL	Apatinib continues to be taken, generally without dose adjustment; Loperamide, compound diphenoxylate (phenethyl piperidine), gastrointestinal mucosal protective agents (e.g., octahedral montmorillonite powder) and berberine may be given as appropriate
**Grade 3**<br>Increase of ≥ 7 stools per day over baseline; hospitalization indicated; severe increase in ostomy output compared to baseline; limiting self care ADL	Stop taking apatinib; Actively stop diarrhea and support symptomatic treatment, pay attention to replenish water and electrolytes, maintain water and electricity balance and prevent acid-base disorder, and replenish nutrition, until diarrhea is significantly alleviated or stopped; The dose of apatinib needs to be appropriately reduced when the drug is resumed.
**Grade 4**<br>Life-threatening consequences; urgent intervention indicated	Immediately and permanently terminate of apatinib; Gastroenterology specialist consultation, active management of diarrhea, and closely monitor of blood pressure and other vital signs
Nausea AE	
Severity of Nausea (NCI-CTCAE v5.0)	Suggestions of prevention and cure

**Grade 1**<br>Loss of appetite without alteration in eating habits	Apatinib continues to be taken, generally without dose adjustment;
**Grade 2**<br>Oral intake decreased without significant weight loss, dehydration or malnutrition	Apatinib continues to be taken, generally without dose adjustment; Active dietary regulation
**Grade 3**<br>Inadequate oral caloric or fluid intake; tube feeding, TPN, or hospitalization indicated	Stop taking apatinib; Actively enteral or parenteral nutrition supplementation until nausea abates or stops; After CTCAE grade regrade to grade 2 or lower, one dose level will be lower.

7.3.3 Matters Needing Attention in Dose Adjustment

1. During the trial, if immune-related toxicity occurs, such as immune pneumonia, hepatitis, colitis, etc., SHR-1210 and apatinib should be suspended as appropriate, and the medication can be resumed when the toxicity recovers grade 1 or baseline level (for ALT/AST and TBIL and other laboratory test indicators are abnormal in the baseline period). The treatment of SHR-1210 should be resumed first. Apatinib administration will be restarted after 7 to 14 days without significant abnormalities, and the subsequent administration mode of apatinib will be adjusted.
2. Grade 3 immune pneumonia or higher, grade 3 TBIL elevation (reoccurring) or higher, grade 4 ALT/AST elevation (reoccurring), other grade 4 immune-related toxicity (except hypothyroidism) and grade 4 injection reaction occurred during the clinical trial or if SHR-1210 has been suspended due to immune-related toxicity for more than 12 weeks and still does not return to grade 1 or baseline level (if baseline period is abnormal), SHR-1210 must be permanently terminated.
3. If the researcher determines that subjects would benefit from apatinib monotherapy after the termination of SHR-1210, subjects will be allowed to receive apatinib monotherapy after toxicity recovery until an event occurs that meets the treatment termination criteria specified in the protocol.
4. In case of grade 3 or higher capillary hyperplasia during the trial period, there is no need for dose adjustment of apatinib, and SHR-1210 should be suspended until toxicity is regraded to grade 2 or lower.

In case of AE clearly related to apatinib, such as hypertension, proteinuria, hand-foot syndrome, etc., during the trial period, apatinib can be suspended, and after toxicity recovery, the original dose of apatinib can be given, the administration mode can be adjusted, or the dose can be terminated. After the termination of apatinib administration, subjects can continue to use SHR-1210 monotherapy.

7.4 Warnings and Precautions

7.4.1 SHR-1210

The safety data for SHR-1210 are derived from nine clinical trials of 986 patients treated with camrelizumab monotherapy, tumor types included esophageal carcinoma (27.5%), hepatocellular carcinoma (22.5%), non-small cell lung cancer (14.8%), nasopharyngeal carcinoma (9.8%), classic Hodgkins lymphoma (7.6%), non -squamous non-small cell lung cancer (5.5%), melanoma (3.7%), gastric cancer (2.7%), lung

adenocarcinoma (1.6%), lung squamous cell carcinoma (1.3%), breast cancer, extranodal NK/T cell lymphoma, bowel cancer, lung cancer, esophageal adenocarcinoma, lung large cell carcinoma, bladder cancer, cholangiocarcinoma, lung endocrine tumor, cervical cancer, esophageal small cell carcinoma (all < 1%). SHR-1210 is given once every 2 weeks at 200 mg (630 cases), 1 mg/kg (13 cases), 3mg/kg (121 cases), 10 mg/kg (12 cases), 60 mg (24 cases), 400 mg (24 cases) and single dose once every 3 weeks at 3 mg/kg (108 cases), 200 mg (54 cases) in the above studies. The incidence of AE of all grades 97% in 986 patients treated with SHR-1210. AE which incidence < 10% include reactive capillary hyperplasia, anemia, fever, hypothyroidism, proteinuria, cough, and loss of appetite. The incidence of grade 3 or higher AE is 24%, incidence > 1% include anemia, hyponatremia, lung infection, elevated AST, elevated γ-GGT, elevated serum bilirubin, elevated conjugated bilirubin, liver function abnormalities, reduction of neutrophil count, reduction of white blood cell count, reduction of platelet count, reduction of lymphocyte count, hypokalemia, elevated ALT, lung inflammation, elevated serum alkaline phosphatase and lipase.

7.4.2 Apatinib

Common AE include hematological system reactions (leukopenia, neutropenia, thrombocytopenia) and non-hematological system reactions (hypertension, proteinuria, hand and foot skin reactions, fatigue, anorexia, diarrhea, bleeding). Most of the AE can be controlled and reversed by suspension of administration, dose reduction and symptomatic management. Hypertension is one of the most common adverse reactions of anti-angiogenic inhibitors, especially VEGF/ VEGFR inhibitors, which can cause secondary hypertension or worsen the existing hypertension. In the phaseII/IIIclinical study of apatinib, the incidence of hypertension is 36.32%, and the incidence of grade 3 hypertension is 5.38%, no grade 4 hypertension is observed. Poor blood pressure control in patients with pre-existing hypertension (>150/100mmHg), or patients with hypertension complicated with thrombosis who need to take anticoagulants for a long time should be cautious in using apatinib. In the phaseII/IIIclinical study of apatinib, the incidence of proteinuria is 44.36%, and grade 3 proteinuria is 1.79%. Proteinuria generally occurs about 3 weeks after apatinib is taken. It is usually asymptomatic and reversible, which can be relieved by suspending the administration or lowering the dose. No serious kidney damage occurs, and special treatment is generally not required. Hand and foot skin reaction is one of the most common clinical skin toxicities in antitumor

molecular targeted drugs. The main symptoms are the numbness of the limbs, burning sensation, erythema, swelling, skin hardens, vesiculation, cracked, and desquamation, with fingers or toes curved skin keratosis characteristics, which is usually bilateral. Symptoms often occur at the same time or in succession, mainly in the palm and plantar, and symptoms are often more significant in these stress areas. Fatigue is often related to the disease itself and the treatment of the tumor. For grade 1 or 2 fatigue, no dose adjustment is required. However, grade 3~4 fatigue requires positive symptomatic treatment and dose adjustment. Progesterone medications (such as megestrol) and multivitamins can be used to help reduce fatigue and improve physical performance. In the phaseII/IIIclinical study of apatinib, the incidence of diarrhea is 10.31%, and the incidence of grade 3-4 diarrhea is 1.35%. In the phaseII/IIIclinical study of apatinib in the treatment of advanced gastric cancer, the incidence of gastrointestinal bleeding in the experimental group and the control group is 2.24% and 3.60%, respectively, with no significant difference. Among them, the incidence of grade 3 and 4 gastrointestinal bleeding is 1.35% and 2.88%, respectively, which generally occurred within the first cycle after taking apatinib.

8.0 Statistical Matters and Analysis Plans

8.1 Primary and Secondary Analysis Endpoints

8.1.1 Analysis Primary Endpoint

The objective response rate (ORR) is defined as the proportion of subjects who achieved best of response (BOR) with a confirmed CR or PR (according to RECIST V1.1). The best of response (BOR) is defined as the best curative effect that is recorded from starting treatment until disease progression (the order is CR, PR, SD, PD or cannot evaluable). Or if PD is not achieved before initiation of subsequent antitumor therapy or before study termination, whichever occurred first, the best efficacy recorded (from the beginning of treatment to the last evaluable tumor imaging examination). BOR outcome is CR or PR needs to be confirmed, defined as CR or PR confirmed by repeated imaging evaluation at least 28 days (4 weeks) after the first observation of CR or PR, with no evidence of PD between the first observation of CR or PR and the confirmed follow-up of CR or PR. Patients who have not had a post-baseline outcome evaluation (for any reason) will be considered non-responders in BOR outcome assessment.

8.1.2 Analysis Secondary Endpoint

1. Disease control rate (DCR) is defined as proportion of subjects who achieved a

BOR of confirmed CR or PR or SD (according to RECIST V1.1).

2. Duration of response (DoR) is defined as the time from the first recorded confirmation of remission (CR or PR) to the first recorded disease progression (according to RECIST V1.1 criteria) or death from any cause, whichever comes first.
3. Progression-free of survival (PFS) is defined as the time from the beginning of treatment until disease progression (according to RECIST V1.1) or death from any cause is recorded, whichever comes first.
4. Overall survival (OS) is defined as the time from enrollment to death from any cause. When recording lost patients before death, up to the time of last recorded contact with the patient.

8.1.3 Safety Analysis

The safety of treatment will be assessed by AE, laboratory tests, vital signs, and imaging examination. All patients who received at least one treatment with any of the study drugs will be included in the safety analyses.

8.2 Sample Size Calculation

Simons optimal 2 -stage design will be used for sample size calculation, with one-side test α=0.05 and power of test=0.8.

(1) Cohort 1: According to previous article, the ORR of RM-NPC treated with anti-PD-1 monoclonal antibody was about 25% after first-line platinum-based chemotherapy failed[14,16-17]. Assuming that SHR-1210 plus apatinib lead to ORR achieved 50% for patients with RM-NPC who failed first-line platinum-based chemotherapy without prior use of anti-PD-1 monoclonal antibody. There were 9 patients enrolled in the first phase. If there were no more than 2 effective cases, the trial would be terminated. Otherwise, the second phase would be entered, and the number of patients enrolled in the second phase would be increased to 24. If there were no more than 9 effective cases (including the effective cases in the first phase), the trial would be terminated. Patients were enrolled for 1 year and followed up for 2 years. Considering the 10% loss to follow-up rate, 27 patients were enrolled in cohort 1.

(2) Cohort 2: Although the previously study found that the response rate of apatinib monotherapy in platinum-resistant NPC was about 30%[28], the objective response rate for PD-1 blockade resistant RM-NPC with apatinib monotherapy was still unkown. Therefore, we assumed that the objective response rate was 20 % with apatinib

monotherapy in cohort 2, which was less than the objective response of 30% for PD-1 blockade-naive RM-NPC treated with apatinib monotherapy. The ORR of RM-NPC treated with apatinib was 20% after previous first-line platinum-based chemotherapy failed. Assuming that SHR-1210 plus apatinib lead to ORR achieved 45% for patients with RM-NPC who failed first-line platinum-based chemotherapy with prior use of anti-PD-1 monoclonal antibody. 10 patients were enrolled in the first phase. If there were no more than 2 effective cases were, the trial would be terminated. Otherwise, the second phase would be entered, and the number of patients enrolled in the second phase would be increased to 22. If there were no more than 7 effective cases (including the first phase), the trial would be terminated. Patients were enrolled for 1 year and followed up for 2 years. Considering the 10% loss to follow-up rate, 25 patients were enrolled in cohort 2.

8.3 Analytical Methods

8.3.1 Type of Analysis

8.3.1.1 Efficacy Analysis

The analysis of efficacy endpoints is based on the full analysis set (FAS), which includes all enrolled subjects who have received at least one study medication and had measurable lesions at baseline (as defined by RECIST V1.1). If the IRRC evaluation data is used for analysis, the IRRC evaluation baseline is used to determine whether there is a measurable lesion. The safety analysis will be performed according to the safety analysis set (SAS), which includes all enrolled subjects who have received at least one study drug treatment.

8.3.1.2 Analysis Methods

1. Analysis of general data

   Evaluate general information such as age, sex, stage, etc.

2. Analysis of adverse events

   NCI-CTC 5.0 standard is used to evaluate the toxicity and complications of the patients.

3. Analytical Approach

   The primary endpoint of this study is ORR assessed according to RECIST (version 1.1). The primary endpoint will be analyzed after the last subject have completed at least 24 weeks of follow-up (the interval between data cutoff date and initial dosing date ≥24 weeks). All the patients who received at least one dose of

camrelizumab and apatinib was included in the full analysis se t (FAS), and the patients who received at least one post-baseline efficacy assessment was included in the efficacy analysis set. Objective response rate, disease control rate, progression-free survival, and overall survival were analyzed both in the full analysis set and efficacy analysis set. Tumor shrinkage rate , time to response, and duration of response were analyzed in the efficacy analysis set. ORR and DCR, 95% accurate bilateral confidence intervals (CI) will be estimated by Clopper Pearson method. Kaplan-Meier method is used to analyze the time to the event endpoint (DoR, PFS and OS). Graphic analysis will include a spider plot of the target tumor load percentage change over time from baseline and a waterfall plot of the optimal percentage change from baseline.

4. Stratification analysis

   Stratification analysis is performed according to the gender, age, WHO classification, ECOG scores, liver metastases, smoking history, previous lines of therapy for advanced diseases, baseline plasma EBV DNA status, previous radiotherapy, previous EGFR inhibitor treatment, PD-L1 expression and VEGF receptor 2 (KDR) expression at baseline.

8.3.2 Safety Data Analysis

All patients who have received at least one treatment and have at least one safety evaluation are included. All safety parameters will be summarized and tabulated according to the safety population

The AEs data are tabulated according to the frequency (overall and intensity) of the human system. Subjects who have the same AE more than once in the total incidence of AEs are counted only once in the frequency table. Statistical analysis of AEs is carried out from the perspectives of total AEs, AEs related to experimental drugs (adverse reactions), AEs resulting in treatment termination and serious AE. The number of cases, cases and incidence are calculated respectively, and the specific record content is given in the form of a list.

The laboratory data will be summarized simultaneously at each sampling time point using a variation table and a frequency table.

All AEs and abnormal laboratory variables will be rated for hematology and chemistry laboratory parametric toxicity symptoms according to the NCI-CTCAE v5.0, and the number and percentage of toxicity levels at each time of visit will be described.

The worst severity level of toxicity before treatment and during each visit are described by cross table, and the number and percentage of cases are given.

9.0 Data Collection and Management

9.1 Requirements for Researchers to Fill Out Clinical Trial Records

For all subjects who have filled in the ICF and are screened as qualified, medical records and case reports should be written carefully and in detail. All items should be filled in without any blank or missing items (see the filling instructions). The data recorded in the case report form should be checked against the medical records and the original examination report. The original medical records (inpatient medical records) and patient diary cards shall be used as the original records, and any corrections shall only be underlined, annotated with modified data and reasons, and signed and dated by the physicians and researchers participating in the clinical trial, and the original records shall not be erased or covered. Data that are significantly high or outside the clinically acceptable range should be verified and explained by the physician participating in the clinical trial.

9.2 Inspectors Examination of Data Records

The inspector should check the ICF and situations of case screening inclusion of the subjects regularly during the trial. Accurate and reliable data collection is ensured by monitoring and cross-checking the CRF against researchers' records by the study inspectors and by reserving a drug dispensing record by the researchers.

The study data are recorded using an electronic case report form through an electronic data capture system (EDC). Confirm that all case reports are filled in correctly and consistent with the original data. All errors or omissions have been corrected or noted, signed and dated by the researchers.

The research center is responsible for data entry into the EDC system. If data inconsistency occurs, the research center will be asked to explain the data, and the research center will answer the data electronically in the EDC system.

Study inspectors (raw data collation) verify and cross-check eCRFs and investigator records and investigator maintained drug dispensing records to ensure accurate and reliable data are collected.

10.0 Quality Control and Quality Assurance

10.1 Quality Control

1. Requirements of participants

The personnel participating in clinical trials should have corresponding professional expertise, qualifications and scientific research ability, and must carefully study and discuss the clinical research plan and trial manual, which will be determined after qualification review. The personnel are relatively fixed.

File keeping, drug use, and calibration of testing instruments need to be managed by qualified personnel.

2. Training of participants

   Through pre-trial training, the participants can fully understand the clinical trial plan and the specific connotation of each indicator. The description of self-conscious symptoms should be objective and should not be induced or prompted. The prescribed objective indicators shall be checked at the time, place and method specified in the plan. Adverse reactions or unanticipated toxic side effects should be observed and followed up.

3. Laboratory quality control measures

   Establish unified test index, standard operating procedure and quality control procedure. The abnormal judgment criteria of laboratory examination shall be based on the normal reference range of each center.

   Special test items must be carried out by special personnel.

4. Other

   Inform the patient of possible adverse reactions to the study medication and inform the physician immediately of any adverse reactions.

10.2 Quality Assurance

1. Establish a multi-center coordination committee

   The multi-center coordination committee is composed of principals of clinical trial units, principal researchers and sponsors. The coordination committee is responsible for the implementation of the entire trial and solving problems related to the trial.

2. Establish the inspection system for clinical inspectors

   CRO shall appoint inspectors of the trial to ensure that the rights and interests of the subjects in the clinical trial are protected, that the data of the trial records and reports are accurate and complete, and that the trial complies with the approved protocol, the GCP and relevant regulations.

Part II: Ethics and General Research Management

11.0 Ethical Matters

11.1 Local Regulations /Declaration of Helsinki

This clinical study will be conducted in compliance with the principles of the Declaration of Helsinki, "Ministerial Ordinance on Good Clinical Practice (GCP)" (Ordinance No. 28 of the Ministry of Health and Welfare dated March 27, 1997) and its revisions, and related notifications ,Chinese laws and regulations to provide patients with greater protection.

11.2 Informed Consent

It is the responsibility of the researchers or researchers' designee to obtain written ICF from each study subject after fully explaining the purpose, methods, expected benefits, and potential harms of the study. For patients who are not qualified or unable to provide legal consent, written ICF must be obtained from the legal guardian. If the patient and his/her legal guardian cannot read, a notary must be present for the entire informed consent discussion. After the patient and guardian verbally agree to participate in the study, the notary sign the ICF certifying that the informed consent information have been accurately interpreted and understood. The researchers or designee must also state that the patient is free to refuse to participate in or withdraw from the study at any time for any reason. If new safety information causes significant changes in the risk/benefit assessment, the ICF should be reviewed and updated as necessary. All patients, including those already under treatment, should be informed of this new information and provided with a revised ICF that gives them consent to continue to participate in the study. In life-threatening cases, the patient is unconscious or unable to communicate, emergency conditions that do not have enough time to get from the patients guardian agree, at the same time also have no other or better treatments are available, so the researchers agreed to allow these patients treated according to the plan, but need record issued by the IEC, that procedures for enrolling patients in these situations have been approved. In addition, the patient or his/her guardian should be informed of the trial and agree to continue as soon as possible, giving written ICF as described above.

11.3 Independent Ethics Committee /Institutional Review Committee

The study protocol and any relevant materials provided to patients (such as a letter

or description of the study), as well as any advertising and compensation offered to patients, will be submitted by the researchers to an independent ethics committee. Ethical consent must be obtained prior to commencement of the study and documented in the form of a letter to the investigator, including providing dates for independent ethics committee meetings and granting of consent. Any modifications to the protocol after receipt of independent ethics committee approval will also be submitted to the independent ethics committee by the researchers in accordance with local procedures and regulations.

12.0 Conditions of Modifying Protocol

All protocol changes, as required, must be submitted to the appropriate independent ethics committee or institutional review committee for consultation and approval. Any protocol modification must be approved before it is implemented, unless the modification can eliminate immediate harm to the study subject, or the modification involves only logistical or administrative aspects of the study (e.g., change of monitor, change of telephone number).

13.0 Conditions for Ending the Study

Both sponsors and researchers have the right to terminate the study at any time. If it is necessary to terminate the study, the study shall be terminated after examination and negotiation by both parties. When terminating studies, sponsors and researchers will ensure that the best interests of patients are taken into account.

14.0 Preservation of Research Documents, Case Reports and Records

14.1 Preservation of Researcher Documents /Records

The researchers must maintain adequate and accurate records so that the conduct of the study can be fully documented and the data can be checked. These documents should be divided into two distinct categories (1) researchers study documents and (2) patient original clinical documents. Researchers study documents include protocol/revision, communication with independent ethics committee/institutional review committee review, samples of ICF, drug records, member resumes and authorization forms, and other relevant documents/letters. In addition, at the end of the study, the researchers will receive patient data, including trace checks, containing a complete record of all data changes, question resolution letters, and reasons for the changes, in a readable format on a CD that must also be stored with the researchers study documents. Patients' original clinical documents (usually previously defined by

the program as documents of key efficacy or safety parameters independent of CRFs). These include patients' inpatient or outpatient records, physician and nurse records, appointment notebooks, original laboratory reports, ECG, EEG, X-ray, pathology, and special assessment reports, signed ICF, advisory letters, and patient screening registration forms. Researchers must file both types of documents for at least 15 years after the study ends or is terminated. After this period, these documents can be processed in accordance with local regulatory requirements.

14.2 Original Documentation and Background Data

The researchers shall provide background data to the sponsor from study documents and clinic records as required. This is especially important when data transcription errors are suspect. In the event of special issues and/or questions or requests for government inspections, it is necessary to access complete study records while ensuring patient confidentiality.

14.3 Inspection

The researchers should be aware of the original documentation of the trial and, under relevant notice, may provide them to the sponsor or its representative, or to the health authority inspector. Case report form data verification must be made by direct inspection of the original documents.

14.4 Case Report Form

For each enrolled patient, the principal researcher or authorized representative of a study member must complete and sign a CRF. It also applies to records of patients who could not complete the study (if a cop of CRF has been initiated, even during the pre-randomized screening phase). If a patient withdraws from the study, the reason must be documented on the CRF. If a patient withdraws from the study because of a treatment-limitingAE, efforts should be made to clearly document the outcome. All forms should be typed or completed in indelible ink and must be legible. Errors should be crossed out, but not overwritten. After correct content inserted, name and date should be signed by the researcher or his/her authorized representative at the corrections. The researchers shall ensure that the CRFs reported to the sponsor and all data required to be reported are accurate, complete, clear and timely.

15.0 Research Monitoring

The inspectors (or representative) will contact and visit the researchers frequently and will be permitted upon request to review various trial records (CRFs and other

related records) while ensuring patient confidentiality as required by the local. The responsibilities of the inspectors are to review the CRF within a time-bound period throughout the study, to verify protocol compliance, and to verify the integrity, consistency, and accuracy of data on the CRFs. Inspectors should review laboratory test reports and other patient records for review of data on CRF. Researchers (or his/her representative) agree to cooperate with the inspectors to ensure that any problems identified during the inspection are resolved.

16.0 Confidentiality of Trial Documents and Patient Records

The researchers must ensure that patient anonymity is maintained and that patient identity is not disclosed to unauthorized parties. On CRFs or other documents submitted to the sponsor, the patient may not be identified by name, only by identifying code. The researchers should record a patient enrollment form showing patient code, name, and address. Researchers should strictly preserve certain documents from being leaking to sponsors, such as ICF from patients.

References:

1. Bray F, Ferlay J, Soerjomataram I et al. Global cancer statistics 2018: GLOBOCAN estimates of incidence and mortality worldwide for 36 cancers in 185 countries. CA Cancer J Clin 2018; 68: 394-424.
2. Chen W, Zheng R, Baade PD et al. Cancer statistics in China, 2015. CA: a cancer journal for clinicians 2016; 66: 115-132.
3. Ng WT, Lee MC, Chang AT et al. The impact of dosimetric inadequacy on treatment outcome of nasopharyngeal carcinoma with IMRT. Oral Oncology 2014; 50: 506-512.
4. Xueming S, Shengfa S, Chunyan C et al. Long-term outcomes of intensity-modulated radiotherapy for 868 patients with nasopharyngeal carcinoma: an analysis of survival and treatment toxicities. Radiotherapy & Oncology 2014; 110: 398-403.
5. Yi J, Huang X, Gao L et al. Intensity-modulated radiotherapy with simultaneous integrated boost for locoregionally advanced nasopharyngeal carcinoma. Radiation Oncology 2014; 9: 56-56.
6. Meng-Xia Z, Jing L, Guo-Ping S et al. Intensity-modulated radiotherapy prolongs the survival of patients with nasopharyngeal carcinoma compared with conventional two-dimensional radiotherapy: A 10-year experience with a large cohort and long follow-up. European Journal of Cancer 2015; 51: 2587-2595.
7. Mao YP, Tang LL, Chen L et al. Prognostic factors and failure patterns in non-metastatic nasopharyngeal carcinoma after intensity-modulated radiotherapy. Chinese Journal of Cancer 2016; 35: 673-682.
8. Lee AWM, Ng WT, Chan LK et al. The strength/weakness of the AJCC/UICC staging system (7th edition) for nasopharyngeal cancer and suggestions for future improvement. Oral Oncology 2012; 48: 1007-1013.
9. Chua MLK, Wee JTS, Hui EP, Chan ATC. Nasopharyngeal carcinoma. Lancet 2016; 387: 1012-1024.
10. Zhang L, Huang Y, Hong S et al. Gemcitabine plus cisplatin versus fluorouracil plus cisplatin in recurrent or metastatic nasopharyngeal carcinoma: a multicentre, randomised, open-label, phase 3 trial. Lancet 2016; 388: 1883-1892.
11. Lee AW, Ma BB, Ng WT, Chan AT. Management of Nasopharyngeal Carcinoma: Current Practice and Future Perspective. J. Clin. Oncol. 2015; 33: 3356-3364.

12. Chen L. Co-inhibitory molecules of the B7-CD28 family in the control of T-cell immunity. Nat. Rev. Immunol. 2004; 4: 336-347.
13. Wang X, Teng F, Kong L, Yu J. PD-L1 expression in human cancers and its association with clinical outcomes. Onco Targets Ther 2016; 9: 5023-5039.
14. Hsu C, Lee SH, Ejadi S et al. Safety and Antitumor Activity of Pembrolizumab in Patients With Programmed Death-Ligand 1-Positive Nasopharyngeal Carcinoma: Results of the KEYNOTE-028 Study. Journal of Clinical Oncology Official Journal of the American Society of Clinical Oncology 2017; 35: JCO2017733675.
15. Wang YQ, Chen YP, Zhang Y et al. Prognostic significance of tumor-infiltrating lymphocytes in non-disseminated nasopharyngeal carcinoma: A large-scale cohort study. International Journal of Cancer 2018.
16. Ma BBY, Lim WT, Goh BC et al. Antitumor Activity of Nivolumab in Recurrent and Metastatic Nasopharyngeal Carcinoma: An International, Multicenter Study of the Mayo Clinic Phase 2 Consortium (NCI-9742). J. Clin. Oncol. 2018; 36: 1412-1418.
17. Fang W, Yang Y, Ma Y et al. Camrelizumab (SHR-1210) alone or in combination with gemcitabine plus cisplatin for nasopharyngeal carcinoma: results from two single-arm, phase 1 trials. Lancet Oncol. 2018; 19: 1338-1350.
18. Kowanetz M, Ferrara N. Vascular endothelial growth factor signaling pathways: therapeutic perspective. Clin. Cancer Res. 2006; 12: 5018-5022.
19. Cheng JZ, Chen JJ, Xue K et al. Clinicopathologic and prognostic significance of VEGF, JAK2 and STAT3 in patients with nasopharyngeal carcinoma. Cancer Cell International 2018; 18: 110-.
20. Wakisaka N, ., Wen QH, Yoshizaki T, . et al. Association of vascular endothelial growth factor expression with angiogenesis and lymph node metastasis in nasopharyngeal carcinoma. Laryngoscope 2010; 109: 810-814.
21. Guang-Wu H, ., Sunagawa M, ., Jie-En L, . et al. The relationship between microvessel density, the expression of vascular endothelial growth factor (VEGF), and the extension of nasopharyngeal carcinoma. Laryngoscope 2010; 110: 2066-2069.
22. Hui EP, Chan ATC, Francesco P et al. Coexpression of hypoxia-inducible factors 1alpha and 2alpha, carbonic anhydrase IX, and vascular endothelial growth factor in nasopharyngeal carcinoma and relationship to survival. Clinical Cancer Research An Official Journal of the American Association for Cancer Research 2002; 8: 2595-2604.
23. Tian S, Quan H, Xie C et al. YN968D1 is a novel and selective inhibitor of vascular endothelial growth factor receptor-2 tyrosine kinase with potent activity in vitro and in

vivo. Cancer Sci. 2011; 102: 1374-1380.
24. X H, J Z, B X et al. Multicenter phase II study of apatinib, a novel VEGFR inhibitor in heavily pretreated patients with metastatic triple-negative breast cancer. International journal of cancer 2014; 135: 1961-1969.
25. Li J, Qin S, Xu J et al. Randomized, Double-Blind, Placebo-Controlled Phase III Trial of Apatinib in Patients With Chemotherapy-Refractory Advanced or Metastatic Adenocarcinoma of the Stomach or Gastroesophageal Junction. J. Clin. Oncol. 2016; 34: 1448-1454.
26. CY L, Y W, Y X et al. Apatinib combined with oral etoposide in patients with platinum-resistant or platinum-refractory ovarian cancer (AEROC): a phase 2, single-arm, prospective study. The Lancet. Oncology 2018; 19: 1239-1246.
27. Lan C-Y, Wang Y, Xiong Y et al. Apatinib combined with oral etoposide in patients with platinum-resistant or platinum-refractory ovarian cancer (AEROC): a phase 2, single-arm, prospective study. The Lancet. Oncology 2018; 19: 1239-1246.
28. Li L, Kong F, Zhang L, Li X, Fu X, Wang X, Wu J, Zhang F, Ren L, Zhang M. Apatinib, a novel VEGFR-2 tyrosine kinase inhibitor, for relapsed and refractory nasopharyngeal carcinoma: data from an open-label, single-arm, exploratory study. Invest New Drugs. 2020 Dec;38(6):1847-1853.
29. Jain RK, Koenig GC, Dellian M et al. Leukocyte-endothelial adhesion and angiogenesis in tumors. Cancer metastasis reviews 1996; 15: 195-204.
30. Melder RJ, Koenig GC, Witwer BP et al. During angiogenesis, vascular endothelial growth factor and basic fibroblast growth factor regulate natural killer cell adhesion to tumor endothelium. Nature medicine 1996; 2: 992-997.
31. Maenhout SK, Thielemans K, Aerts JL. Location, location, location: functional and phenotypic heterogeneity between tumor-infiltrating and non-infiltrating myeloid-derived suppressor cells. Oncoimmunology 2014; 3: e956579.
32. Chaudhary B, Khaled YS, Ammori BJ, Elkord E. Neuropilin 1: function and therapeutic potential in cancer. Cancer immunology, immunotherapy : CII 2014; 63: 81-99.
33. Voron T, Colussi O, Marcheteau E et al. VEGF-A modulates expression of inhibitory checkpoints on CD8+ T cells in tumors. The Journal of experimental medicine 2015; 212: 139-148.
34. Gabrilovich D, Ishida T, Oyama T et al. Vascular endothelial growth factor inhibits the development of dendritic cells and dramatically affects the differentiation of

multiple hematopoietic lineages in vivo. Blood 1998; 92: 4150-4166.
35. Yasuda S, Sho M, Yamato I et al. Simultaneous blockade of programmed death 1 and vascular endothelial growth factor receptor 2 (VEGFR2) induces synergistic anti-tumour effect in vivo. Clin. Exp. Immunol. 2013; 172: 500-506.
36. Ott PA, Hodi FS, Buchbinder EI. Inhibition of Immune Checkpoints and Vascular Endothelial Growth Factor as Combination Therapy for Metastatic Melanoma: An Overview of Rationale, Preclinical Evidence, and Initial Clinical Data. Frontiers in Oncology 2015; 5: 202.
37. Fukumura D, Kloepper J, Amoozgar Z et al. Enhancing cancer immunotherapy using antiangiogenics: opportunities and challenges. Nature reviews. Clinical oncology 2018; 15: 325-340.
38. Huang Y, Kim BYS, Chan CK et al. Improving immune-vascular crosstalk for cancer immunotherapy. Nature reviews. Immunology 2018; 18: 195-203.
39. Yi M, Jiao D, Qin S et al. Synergistic effect of immune checkpoint blockade and anti-angiogenesis in cancer treatment. Molecular cancer 2019; 18: 60.
40. Reck M, Mok TSK, Nishio M et al. Atezolizumab plus bevacizumab and chemotherapy in non-small-cell lung cancer (IMpower150): key subgroup analyses of patients with EGFR mutations or baseline liver metastases in a randomised, open-label phase 3 trial. The Lancet. Respiratory medicine 2019; 7: 387-401.
41. Xu J, Zhang Y, Jia R et al. Anti-PD-1 Antibody SHR-1210 Combined with Apatinib for Advanced Hepatocellular Carcinoma, Gastric, or Esophagogastric Junction Cancer: An Open-label, Dose Escalation and Expansion Study. Clin. Cancer Res. 2019; 25: 515-523.

Appendix 1) RECIST Version 1.1

1. Measurability of tumour at baseline

1.1. Definitions

At baseline, tumour lesions/lymph nodes will be categorized measurable or non-measurable as follows:

1.1.1. Measurable

Tumour lesions: Must be accurately measured in at least one dimension (longest diameter in the plane of measurement is to be recorded) with a minimum size of:

- 10 mm by CT scan (CT scan slice thickness no greater than 5 mm; see Appendix II on imaging guidance).
- 10 mm caliper measurement by clinical exam (lesions which cannot be accurately measured with calipers should be recorded as non-measurable).
- 20 mm by chest X-ray.

Malignant lymph nodes: To be considered pathologically enlarged and measurable, a lymph node must be P15 mm in short axis when assessed by CT scan (CT scan slice thickness recommended to be no greater than 5 mm). At baseline and in follow-up, only the short axis will be measured and followed. See also notes below on '2.2 Baseline documentation of target and non-target lesions' for information on lymph node measurement.

1.1.2. Non-measurable

All other lesions, including small lesions (longest diameter <10 mm or pathological lymph nodes with P10 to <15 mm short axis) as well as truly non-measurable lesions. Lesions considered truly non-measurable include: leptomeningeal disease, ascites, pleural or pericardial effusion, inflammatory breast disease, lymphangitic involvement of skin or lung, abdominal masses/abdominal organomegaly identified by physical exam that is not measurable by reproducible imaging techniques.

1.1.3. Special considerations regarding lesion measurability

Bone lesions, cystic lesions, and lesions previously treated with local therapy require particular comment:

Bone lesions:

• Bone scan, PET scan or plain films are not considered adequate imaging techniques to measure bone lesions. However, these techniques can be used to confirm the presence or disappearance of bone lesions.

• Lytic bone lesions or mixed lytic-blastic lesions, with identifiable soft tissue components, that can be evaluated by cross sectional imaging techniques such as CT or MRI can be considered as measurable lesions if the soft tissue component meets the definition of measurability described above.

• Blastic bone lesions are non-measurable.

Cystic lesions:

• Lesions that meet the criteria for radiographically defined simple cysts should not be considered as malignant lesions (neither measurable nor non-measurable) since they are, by definition, simple cysts.

• 'Cystic lesions' thought to represent cystic metastases can be considered as measurable lesions, if they meet the definition of measurability described above. However, if non-cystic lesions are present in the same patient, these are preferred for selection as target lesions.

Lesions with prior local treatment:

• Tumour lesions situated in a previously irradiated area, or in an area subjected to other loco-regional therapy, are usually not considered measurable unless there has been demonstrated progression in the lesion. Study protocols should detail the conditions under which such lesions would be considered measurable.

1.2. Specifications by methods of measurements

1.2.1. Measurement of lesions

All measurements should be recorded in metric notation, using calipers if clinically assessed. All baseline evaluations should be performed as close as possible to the treatment start and never more than 4 weeks before the beginning of the treatment.

1.2.2. Method of assessment

The same method of assessment and the same technique should be used to characterize each identified and reported lesion at baseline and during follow-up. Imaging based evaluation should always be done rather than clinical examination unless the lesion(s) being followed cannot be imaged but are assessable by clinical exam.

Clinical lesions: Clinical lesions will only be considered measurable when they are

superficial and P10 mm diameter as assessed using calipers (e.g., skin nodules). For the case of skin lesions, documentation by colour photography including a ruler to estimate the size of the lesion is suggested.

Chest X-ray: Chest CT is preferred over chest X-ray, particularly when progression is an important endpoint, since CT is more sensitive than X-ray, particularly in identifying new lesions. However, lesions on chest X-ray may be considered measurable if they are clearly defined and surrounded by aerated lung.

CT, MRI: CT is the best currently available and reproducible method to measure lesions selected for response assessment. This guideline has defined measurability of lesions on CT scan based on the assumption that CT slice thickness is 5 mm or less. When CT scans have slice thickness greater than 5 mm, the minimum size for a measurable lesion should be twice the slice thickness. MRI is also acceptable in certain situations. If prior to enrolment it is known a patient is not able to undergo CT scans with IV contrast due to allergy or renal insufficiency, the decision as to whether a non-contrast CT or MRI (with or without IV contrast) should be used to evaluate the subject at baseline and follow-up should be guided by the tumour type under investigation and the anatomic location of the disease. For patients who develop contraindications to contrast after baseline contrast CT is done, the decision as to whether non-contrast CT or MRI (enhanced or non-enhanced) should be performed should also be based on the tumour type, anatomic location of the disease and should be optimised to allow for comparison to the prior studies if possible. Each case should be discussed with the radiologist to determine if substitution of these other approaches is possible and, if not, the patient should be considered not evaluable from that point forward.

Ultrasound: Ultrasound is not useful in assessment of lesion size and should not be used as a method of measurement.

Endoscopy, laparoscopy, tumour markers, cytology, histology: Objective tumor assessment using these techniques is generally not recommended, but may be determined based on the study design.

2. Tumour response evaluation

2.1. Assessment of overall tumour burden and measurable disease

To assess objective response or future progression, it is necessary to estimate the overall tumour burden at baseline and use this as a comparator for subsequent measurements. Measurable disease is defined by the presence of at least one measurable

lesion (as detailed above in Section 1.1.1).

2.2. Baseline documentation of 'target' and 'non-target' lesions

When more than one measurable lesion is present at baseline all lesions up to a maximum of five lesions total (and a maximum of two lesions per organ) representative of all involved organs should be identified as target lesions and will be recorded and measured at baseline (this means in instances where patients have only one or two organ sites involved a maximum of two and four lesions respectively will be recorded). Other lesions in this organ (although measurable) will be recorded as undetectable (even if CT scan size is greater than 10 mm).

Target lesions should be selected on the basis of their size (lesions with the longest diameter), be representative of all involved organs, but in addition should be those that lend themselves to reproducible repeated measurements. It may be the case that, on occasion, the largest lesion does not lend itself to reproducible measurement in which circumstance the next largest lesion which can be measured reproducibly should be selected.

Lymph nodes merit special mention since they are normal anatomical structures which may be visible by imaging even if not involved by tumour. As noted in Section 1.1.1, pathological nodes which are defined as measurable and may be identified as target lesions must meet the criterion of a short axis of P15 mm by CT scan. Only the short axis of these nodes will contribute to the baseline sum. The short axis of the node is the diameter normally used by radiologists to judge if a node is involved by solid tumour. Nodal size is normally reported as two dimensions in the plane in which the image is obtained (for CT scan this is almost always the axial plane; for MRI the plane of acquisition may be axial, saggital or coronal). The smaller of these measures is the short axis. For example, an abdominal node which is reported as being 20 mm · 30 mm has a short axis of 20 mm and qualifies as a malignant, measurable node. In this example, 20 mm should be recorded as the node measurement. All other pathological nodes (those with short axis P10 mm but <15 mm) should be considered non-target lesions. Nodes that have a short axis <10 mm are considered non-pathological and should not be recorded or followed.

A sum of the diameters (longest for non-nodal lesions, short axis for nodal lesions) for all target lesions will be calculated and reported as the baseline sum diameters. If lymph nodes are to be included in the sum, then as noted above, only the short axis is

added into the sum. The baseline sum diameters will be used as reference to further characterize any objective tumour regression in the measurable dimension of the disease.

All other lesions (or sites of disease) including pathological lymph nodes should be identified as non-target lesions and should also be recorded at baseline. Measurements are not required and these lesions should be followed as 'present', 'absent', or in rare cases 'unequivocal progression' (as detailed in Section 2.3.4). In addition, it is possible to record multiple non target lesions involving the same organ as a single item on the case record form (e.g., 'multiple enlarged pelvic lymph nodes' or 'multiple liver metastases').

2.3. Response criteria

This section provides the definitions of the criteria used to determine objective tumour response for target lesions.

2.3.1. Evaluation of target lesions

Complete Response (CR): Disappearance of all target lesions. Any pathological lymph nodes (whether target or non-target) must have reduction in short axis to <10 mm.

Partial Response (PR): At least a 30% decrease in the sum of diameters of target lesions, taking as reference the baseline sum diameters.

Progressive Disease (PD): At least a 20% increase in the sum of diameters of target lesions, taking as reference the smallest sum on study (this includes the baseline sum if that is the smallest on study). In addition to the relative increase of 20%, the sum must also demonstrate an absolute increase of at least 5 mm. (Note: the appearance of one or more new lesions is also considered progression).

Stable Disease (SD): Neither sufficient shrinkage to qualify for PR nor sufficient increase to qualify for PD, taking as reference the smallest sum diameters while on study.

2.3.2. Special notes on the assessment of target lesions

Lymph nodes. Lymph nodes identified as target lesions should always have the actual short axis measurement recorded (measured in the same anatomical plane as the baseline examination), even if the nodes regress to below 10 mm on study. This means that when lymph nodes are included as target lesions, the 'sum' of lesions may not be zero even if complete response criteria are met, since a normal lymph node is defined

as having a short axis of <10 mm.

Target lesions that become 'too small to measure'. While on study, all lesions (nodal and non-nodal) recorded at baseline should have their actual measurements recorded at each subsequent evaluation, even when very small (e.g., 2 mm). However, sometimes lesions or lymph nodes which are recorded as target lesions at baseline become so faint on CT scan that the radiologist may not feel comfortable assigning an exact measure and may report them as being 'too small to measure'. When this occurs, it is important that a value be recorded on the case report form.

1. If it is the opinion of the radiologist that the lesion has likely disappeared, the measurement should be recorded as 0 mm.
2. If the lesion is believed to be present and is faintly seen but too small to measure, a default value of 5 mm should be assigned (Note: It is less likely that this rule will be used for lymph nodes since they usually have a definable size when normal and are frequently surrounded by fat such as in the retroperitoneum; however, if a lymph node is believed to be present and is faintly seen but too small to measure, a default value of 5 mm should be assigned in this circumstance as well).

To reiterate, however, if the radiologist is able to provide an actual measure, that should be recorded, even if it is below 5 mm.

Lesions that split or coalesce on treatment. When non-nodal lesions 'fragment', the longest diameters of the fragmented portions should be added together to calculate the target lesion sum. Similarly, as lesions coalesce, a plane between them may be maintained that would aid in obtaining maximal diameter measurements of each individual lesion. If the lesions have truly coalesced such that they are no longer separable, the vector of the longest diameter in this instance should be the maximal longest diameter for the 'coalesced lesion'.

2.3.3. Evaluation of non-target lesions

This section provides the definitions of the criteria used to determine the tumour response for the group of non-target lesions. While some non-target lesions may actually be measurable, they need not be measured and instead should be assessed only qualitatively at the time points specified in the protocol.

Complete Response (CR): Disappearance of all non-target lesions and normalization of tumour marker level. All lymph nodes must be non-pathological in size (<10 mm short axis).

Non-CR/Non-PD: Persistence of one or more non-target lesion(s) and/or maintenance of tumour marker level above the normal limits.

Progressive Disease (PD): Unequivocal progression (see comments below) of existing non-target lesions. (Note: the appearance of one or more new lesions is also considered progression).

2.3.4. Special notes on assessment of progression of non-target disease

When the patient also has measurable disease. In this setting, to achieve 'unequivocal progression' on the basis of the non-target disease, there must be an overall level of substantial worsening in non-target disease such that, even in presence of SD or PR in target disease, the overall tumour burden has increased sufficiently to merit discontinuation of therapy. A modest 'increase' in the size of one or more non-target lesions is usually not sufficient to quality for unequivocal progression status. The designation of overall progression solely on the basis of change in non-target disease in the face of SD or PR of target disease will therefore be extremely rare.

When the patient has only non-measurable disease, this circumstance arises in some phase III trials when it is not a criterion of study entry to have measurable disease. The same general concepts apply here as noted above, however, in this instance there is no measurable disease assessment to factor into the interpretation of an increase in non-measurable disease burden. Because worsening in non-target disease cannot be easily quantified (by definition: if all lesions are truly non-measurable) a useful test that can be applied when assessing patients for unequivocal progression is to consider if the increase in overall disease burden based on the change in non-measurable disease is comparable in magnitude to the increase that would be required to declare PD for measurable disease: i.e., an increase in tumour burden representing an additional 73% increase in 'volume' (which is equivalent to a 20% increase diameter in a measurable lesion). Examples include an increase in a pleural effusion from 'trace' to 'large', an increase in lymphangitic disease from localized to widespread, or may be described in protocols as 'sufficient to require a change in therapy'. Some illustrative examples are shown in Figs. 5 and 6 in Appendix II. If 'unequivocal progression' is seen, the patient should be considered to have had overall PD at that point. While it would be ideal to have objective criteria to apply to non-measurable disease, the very nature of that disease makes it impossible to do so, therefore the increase must be substantial.

2.3.5 New lesions

The appearance of new malignant lesions denotes disease progression; therefore, some comments on detection of new lesions are important. There are no specific criteria for the identification of new radiographic lesions; however, the finding of a new lesion should be unequivocal: i.e., not attributable to differences in scanning technique, change in imaging modality or findings thought to represent something other than tumour (for example, some 'new' bone lesions may be simply healing or flare of pre-existing lesions). This is particularly important when the patient's baseline lesions show partial or complete response. For example, necrosis of a liver lesion may be reported on a CT scan report as a 'new' cystic lesion, which it is not.

A lesion identified on a follow-up study in an anatomical location that was not scanned at baseline is considered a new lesion and will indicate disease progression.

If a new lesion is equivocal, for example because of its small size, continued therapy and follow-up evaluation will clarify if it represents truly new disease. If repeat scans confirm there is definitely a new lesion, then progression should be declared using the date of the initial scan.

2.4 Evaluation of response

2.4.1. Time point response

It is assumed that at each protocol specified time point, a response assessment occurs. Table 1 on the next page provides a summary of the overall response status calculation at each time point for patients who have measurable disease at baseline.

When patients have non-measurable (therefore non-target) disease only, Table 2 is to be used.

Table 1: Time point response: patients with target (+/–non-target) disease.

Target lesions	Non-target lesions	New leisions	Overall response
CR	CR	No	CR
CR	Non-CR/non-PD	No	PR
CR	Not evaluated	No	PR
PR	Non-PD or not all evaluated	No	PR
SD	Non-PD or not all evaluated	No	SD

Not all evaluated	Non-PD	No	NE
PD	Any	Yes or No	PD
Any	PD	Yes or No	PD
Any	Any	Yes	PD

CR = complete reponse, PR = partial response, SD = stable disease, PD = progressive disease, and NE = inevaluable.

Table 2: Time point response: patients with non-target disease only.

Non-target lesions	New leisions	Overall response
CR	No	CR
Non-CR/non-PD	No	Non-CR/non-PD[a]
Not all evaluated	No	NE
Unequivocal PD	Yes or No	PD
Any	Yes	PD

CR = complete reponse, PD = progressive disease, and NE = inevaluable.

a 'Non-CR/non-PD' is preferred over 'stable disease' for non-target disease since SD is increasingly used as endpoint for assessment of efficacy in some trials so to assign this category when no lesions can be measured is not advised

2.4.2. Missing assessments and inevaluable designation

When no imaging/measurement is done at all at a particular time point, the patient is not evaluable (NE) at that time point. If only a subset of lesion measurements is made at an assessment, usually the case is also considered NE at that time point, unless a convincing argument can be made that the contribution of the individual missing lesion(s) would not change the assigned time point response. This would be most likely to happen in the case of PD. For example, if a patient had a baseline sum of 50 mm with three measured lesions and at follow-up only two lesions were assessed, but those gave a sum of 80 mm, the patient will have achieved PD status, regardless of the contribution of the missing lesion.

If one or more target lesions are not evaluated because there is no scan, or cannot be evaluated because of poor image quality or limited field of view, then the efficacy evaluation of the target lesion should be "not evaluated" because the patient is not evaluated. Similarly, if one or more non-target lesions are indicated as "unassessed", the non-target response should be "not evaluated" (unless significant progression occurs). If the target response or non-target response is " not evaluated ", then the

overall efficacy response should be " not evaluated " (unless this is clear evidence of disease progression), as this is equivalent to being inevaluable at that point in time.

Table 3 – Best overall response when confirmation of CR and PR required.

Overall response<br>First time point	Overall response<br>Subsequent time point	BEST overall response
CR	CR	CR
CR	PR	SD, PD or PR[a]
CR	SD	SD provided minimum criteria for SD duration met, otherwise, PD
CR	PD	SD provided minimum criteria for SD duration met, otherwise, PD
CR	NE	SD provided minimum criteria for SD duration met, otherwise, NE
PR	CR	PR
PR	PR	PR
PR	SD	SD
PR	PD	SD provided minimum criteria for SD duration met, otherwise, PD
PR	NE	SD provided minimum criteria for SD duration met, otherwise, NE
NE	NE	NE

CR = complete reponse, PR = partial response, SD = stable disease, PD = progressive disease, and NE = inevaluable.

a If a CR is truly met at first time point, then any disease seen at a subsequent time point, even disease meeting PR criteria relative to baseline, makes the disease PD at that point (since disease must have reappeared after CR). Best response would depend on whether minimum duration for SD was met. However, sometimes 'CR' may be claimed when subsequent scans suggest small lesions were likely still present and in fact the patient had PR, not CR at the first time point.

Under these circumstances, the original CR should be changed to PR and the best response is PR.

3. Special notes on response assessment

When nodal disease is included in the sum of target lesions and the nodes decrease to ‘normal’ size (<10 mm), they may still have a measurement reported on scans. This measurement should be recorded even though the nodes are normal in order not to overstate progression should it be based on increase in size of the nodes. As noted earlier, this means that patients with CR may not have a total sum of ‘zero’ on the case report form (CRF).

Patients with a global deterioration of health status requiring discontinuation of treatment without objective evidence of disease progression at that time should be reported as ‘symptomatic deterioration’. Every effort should be made to document objective progression even after discontinuation of treatment. Symptomatic deterioration is not a descriptor of an objective response: it is a reason for stopping study therapy. The objective response status of such patients is to be determined by evaluation of target and non-target disease as shown in Tables 1–3.

For equivocal findings of progression (e.g., very small and uncertain new lesions; cystic changes or necrosis in existing lesions), treatment may continue until the next scheduled assessment. If at the next scheduled assessment, progression is confirmed, the date of progression should be the earlier date when progression was suspected.

When the primary disease persists or is partially present in enrolled patients with advanced disease, the primary tumor should be appropriately documented in the target or non-target lesion. This is to avoid using statistical procedures to misevaluate complete overall efficacy when the primary disease is still present but cannot be evaluated.

Appendix 2) ECOG Performance Status Scale

Grade	Description
0	Normal activity. Fully active, able to carry on all pre-disease performance without restriction.
1	Symptoms, but ambulatory. Restricted in physically strenuous activity, but ambulatory and able to carry out work of a light or sedentary nature (e.g., light housework, office work).
2	In bed < 50% of the time. Ambulatory and capable of all self-care, but unable to carry out any work activities. Up and about more than 50% of waking hours.
3	In bed > 50% of the time. Capable of only limited self-care, confined to bed or chair more than 50% of waking hours.
4	100% bedridden. Completely disabled. Cannot carry on any self-care. Totally confined to bed or chair.
5	Dead.
