# Supplementary material for: Camrelizumab combined with apatinib in patients with first-line platinum-resistant or PD-1 inhibitor resistant recurrent/metastatic nasopharyngeal carcinoma: a single-arm, phase 2 trial
Source: Nat Commun. 2023 Aug 14;14:4893. doi: 10.1038/s41467-023-40402-x (PMC10425437; doi:10.1038/s41467-023-40402-x)
Supplement: Supplementary file 3 — Description of Additional Supplementary Files [file 41467_2023_40402_MOESM3_ESM.pdf]

Description of Additional Supplementary Files

Supplementary Data 1. Differentially expressed genes of baseline tumor specimens in cohort 1 by response.

Response as indicated with responders (Rs) defined as having a complete or partial response by RECIST 1.1, and non-responders (NRs) as having less than partial response (n = 9 Rs and 4 NRs). A cutoff fold change (log_2-transformed) of > 2 or < −2 and a false discovery rate (FDR) q-value of < 0.05 were applied, and only genes that met these criteria were listed. DESeq2 (v1.32.0) software was used to identify DEGs between the Rs and NRs. Shown are: baseMean, mean normalized counts averaged over all samples from both conditions; Log2FoldChange, the logarithm (to base 2) of the fold change; lfcSE, standard error estimate for the log2 fold change estimate; stat, Wald statistic; P.Value, P value for the statistical significance of this change; Adj.P.Value, P value adjusted for multiple testing with the Benjamini-Hochberg procedure which controls FDR.

Supplementary Data 2. MCP-Counter geneset.

Gene markers for MCP-counter methodology to produce an abundance score for eight immune cell types, endothelial cells and fibroblasts. Listed are the HUGO symbols, ENTREZ ID and ENSEMBL ID for each gene and the identification of cell population to which the gene is assigned.

Supplementary Data 3. MCP-counter outputs in baseline and relapse tumor specimens of NPC patients in all 44 samples.

MCP-counter output scores for ten cell populations (eight immune cell types, endothelial cells and fibroblasts) of tumor specimens from all 48 tumor specimens: cohort 1 (PD-1 inhibitor-naive, n = 12 Rs and 5 NRs at baseline; n = 5 Rs and 4 NRs at post-treatment) and cohort 2 (PD-1 inhibitor- resistance, n = 9 Rs and 7 NRs at baseline; n = 4 Rs and 2 NRs at post-treatment) NPC patients. Sample ID, cohort, time

point and response as indicated. The column “Cluster” demonstrated the 11 pairs of baseline and residual specimens from the same patient. Heatmaps were plotted using the output data for *z*-score normalization**.**

Supplementary Data 4. Differentially expressed genes at baseline tumor specimens in enrolled patients by treatment cohort.

A cutoff fold change (log_2-transformed) of > 2 or < -2 and a false discovery rate (FDR) *q*-value of < 0.05 were applied, and only genes that met these criteria were listed. DESeq2 (v1.32.0) software was used to identify DEGs between the cohort 1 (*n*=13) and cohort 2 (*n*=13). Shown are: baseMean, mean normalized counts averaged over all samples from both conditions; Log2FoldChange, the logarithm (to base 2) of the fold change; IfcSE, standard error estimate for the log2 fold change estimate; stat, Wald statistic; P.Value, *P* value for the statistical significance of this change; Adj.P.Value, *P* value adjusted for multiple testing with the Benjamini-Hochberg procedure which controls FDR.

Supplementary Data 5. Differentially expressed genes of responders' tumor specimens between baseline and post-treatment in both cohorts.

A cutoff fold change (log_2-transformed) of > 2 or < -2 and a false discovery rate (FDR) *q*-value of < 0.05 were applied, and only genes that met these criteria were listed. DESeq2 (v1.32.0) software was used to identify DEGs between the baseline (*n*=7) and post-treatment (*n*=7). Shown are: b
aseMean, mean normalized counts averaged over all samples from both conditions; Log2FoldChange, the logarithm (to base 2) of the fold change; IfcSE, standard error estimate for the log2 fold change estimate; stat, Wald statistic; P.Value, *P* value for the statistical significance of this change; Adj.P.Value, *P* value adjusted for multiple testing with the Benjamini-Hochberg procedure which controls FDR.

Supplementary Data 6. Differentially expressed genes of non-responders' tumor specimens between baseline and post-treatment in both cohorts.

A cutoff fold change (log_2-transformed) of > 2 or < −2 and a false discovery rate (FDR) *q*-value of < 0.05 were applied, and only genes that met these criteria were listed. DESeq2 (v1.32.0) software was used to identify DEGs between the baseline (*n*=4) and post-treatment (*n*=4). Shown are: baseMean, mean normalized counts averaged over all samples from both conditions; Log2FoldChange, the logarithm (to base 2) of the fold change; lfcSE, standard error estimate for the log2 fold change estimate; stat, Wald statistic; P.Value, *P* value for the statistical significance of this change; Adj.P.Value, *P* value adjusted for multiple testing with the Benjamini-Hochberg procedure which controls FDR.
