# Supplementary material for: Camrelizumab combined with apatinib in patients with first-line platinum-resistant or PD-1 inhibitor resistant recurrent/metastatic nasopharyngeal carcinoma: a single-arm, phase 2 trial
Source: Nat Commun. 2023 Aug 14;14:4893. doi: 10.1038/s41467-023-40402-x (PMC10425437; doi:10.1038/s41467-023-40402-x)
Supplement: Supplementary file 10 — Reporting Summary [file 41467_2023_40402_MOESM10_ESM.pdf]

nature portfolio

Corresponding author(s): Hai-Qiang Mai

Last updated by author(s): Jul 21, 2023

Reporting Summary

Nature Portfolio wishes to improve the reproducibility of the work that we publish. This form provides structure for consistency and transparency in reporting. For further information on Nature Portfolio policies, see our Editorial Policies and the Editorial Policy Checklist.

Statistics

For all statistical analyses, confirm that the following items are present in the figure legend, table legend, main text, or Methods section.

n/a	Confirmed	
☐	☒	The exact sample size (*n*) for each experimental group/condition, given as a discrete number and unit of measurement
☒	☐	A statement on whether measurements were taken from distinct samples or whether the same sample was measured repeatedly
☐	☒	The statistical test(s) used AND whether they are one- or two-sided<br>*Only common tests should be described solely by name; describe more complex techniques in the Methods section.*
☒	☐	A description of all covariates tested
☒	☐	A description of any assumptions or corrections, such as tests of normality and adjustment for multiple comparisons
☐	☒	A full description of the statistical parameters including central tendency (e.g. means) or other basic estimates (e.g. regression coefficient) AND variation (e.g. standard deviation) or associated estimates of uncertainty (e.g. confidence intervals)
☐	☒	For null hypothesis testing, the test statistic (e.g. *F*, *t*, *r*) with confidence intervals, effect sizes, degrees of freedom and *P* value noted<br>*Give P values as exact values whenever suitable.*
☒	☐	For Bayesian analysis, information on the choice of priors and Markov chain Monte Carlo settings
☒	☐	For hierarchical and complex designs, identification of the appropriate level for tests and full reporting of outcomes
☐	☒	Estimates of effect sizes (e.g. Cohen's *d*, Pearson's *r*), indicating how they were calculated

Our web collection on statistics for biologists contains articles on many of the points above.

Software and code

Policy information about availability of computer code

Data collection	The data in this study were collected using the EpiData 3.1.
Data analysis	The data in this study were analyzed using SPSS (version 22.0) and R (version 4.2.1). Cell quantification was performed across whole tumor sections using Halo analysis software (PANOVU).

For manuscripts utilizing custom algorithms or software that are central to the research but not yet described in published literature, software must be made available to editors and reviewers. We strongly encourage code deposition in a community repository (e.g. GitHub). See the Nature Portfolio guidelines for submitting code & software for further information.

Data

Policy information about availability of data

All manuscripts must include a data availability statement. This statement should provide the following information, where applicable:

- Accession codes, unique identifiers, or web links for publicly available datasets
- A description of any restrictions on data availability
- For clinical datasets or third party data, please ensure that the statement adheres to our policy

All requests for data will be reviewed by the clinical site Sun Yat-sen University Cancer Center, to verify if the request is subject to any intellectual property or confidentiality obligations. The trial protocol is available in the supplementary note 1. The full IHC/MIHC dataset could also be shared upon request. The raw sequence data for this paper has been deposited in the Genome Sequence Archive (Genomics, Proteomics & Bioinformatics 2021) at the National Genomics Data Center (Nucleic Acids Res 2022), China National Center for Bioinformation / Beijing Institute of Genomics, Chinese Academy of Sciences [66, 67] (GSA-Human:

HRA004738). The sequencing data can be accessed through GSA under the accession code HRA004738 [https://ngdc.cncb.ac.cn/gsa-human/browse/HRA004738]. Sequencing data are available under restricted access. Access can be obtained by completing the application form via GSA-Human System and/or by contacting the corresponding authors. Clinical data are not publicly available due to involving patient privacy, but can be accessed on request from the corresponding author Hai-Qiang Mai for 10years; individual de-identified participant data will be shared. All requests for data will be reviewed by the leading clinical site Sun Yat-Sen University Cancer Center and the study sponsor, Jiangsu Hengrui Pharmaceuticals, to verify if the request is subject to any intellectual property or confidentiality obligations. Requests for access to the patient-level data from this study can be submitted via email to maihq@sysucc.org.cn with detailed proposal for approval. A signed data access agreement with the sponsor is required before accessing the shared data.The remaining data are available within the Article, Supplementary Information, and Source Data. Source data are provided with this paper.

Research involving human participants, their data, or biological material

Policy information about studies with human participants or human data. See also policy information about sex, gender (identity/presentation), and sexual orientation and race, ethnicity and racism.

Reporting on sex and gender	Sex was self-report and was not considered in study design. We confirm that the patients characteristics in the supplementary tables also include sex.
Reporting on race, ethnicity, or other socially relevant groupings	The manuscript used gender and age as the socially constructed or socially relevant categorization variables.The study aimed to examine the effect of gender and age on ORR and survival in different cohorts.Gender was self-reported by participants as male or female. Age was also self-reported as a continuous variable in years.There was no confounding variables in our analyses.The study did not onfounding variables, such as education, income, political ideology, and geographic region.
Population characteristics	Patients with recurrent or metastatic nasopharyngeal carcinoma who had failed first-line platinum-based chemotherapy or were resistant to Anti-PD-1 monoclonal antibody were enrolled.The mean age was 45 years, and all patients had previously received at least first-line platinum-based treatment for RM NPC.
Recruitment	Participants were recruited from the Guangzhou area (China) via posters, emails, flyers, social media, and website advertisements. 72 participants were recruited into the trial after signing a written informed consent.Because all the participants were recruited from the Guangzhou area, they might not have been representative of the wider population. No other potential self-selection bias was present in this trial.
Ethics oversight	The trial was approved by the Research Ethics Board of Sun Yat-sen University Cancer Center and was done in accordance with the Declaration of Helsinki. All patients provided written informed consent.

Note that full information on the approval of the study protocol must also be provided in the manuscript.

Field-specific reporting

Please select the one below that is the best fit for your research. If you are not sure, read the appropriate sections before making your selection.

[x] Life sciences   [ ] Behavioural & social sciences   [ ] Ecological, evolutionary & environmental sciences

For a reference copy of the document with all sections, see nature.com/documents/nr-reporting-summary-flat.pdf

Life sciences study design

All studies must disclose on these points even when the disclosure is negative.

Sample size

N=72. Simon's optimal 2-stage design will be used for sample size calculation, with one-side test α=0.05 and power of test=0.8.

(1) Cohort 1: According to previous article, the ORR of RM-NPC treated with anti-PD-1 monoclonal antibody was about 25% after first-line platinum-based chemotherapy failed[14,16-17]. Assuming that SHR-1210 plus apatinib lead to ORR achieved 50% for patients with RM-NPC who failed first-line platinum-based chemotherapy without prior use of anti-PD-1 monoclonal antibody. There were 9 patients enrolled in the first phase. If there were no more than 2 effective cases, the trial would be terminated. Otherwise, the second phase would be entered, and the number of patients enrolled in the second phase would be increased to 24. If there were no more than 9 effective cases (including the effective cases in the first phase), the trial would be terminated. Patients were enrolled for 1 year and followed up for 2 years. Considering the 10% loss to follow-up rate, 27 patients were enrolled in cohort 1.

(2) Cohort 2: Although the previously study found that the response rate of apatinib monotherapy in platinum-resistant NPC was about 30% [28], the objective response rate for PD-1 blockade resistant RM-NPC with apatinib monotherapy was still unkown. Therefore, we assumed that the objective response rate was 20 % with apatinib monotherapy in cohort 2, which was less than the objective response of 30% for PD-1 blockade-naive RM-NPC treated with apatinib monotherapy. The ORR of RM-NPC treated with apatinib was 20% after previous first-line platinum-based chemotherapy failed. Assuming that SHR-1210 plus apatinib lead to ORR achieved 45% for patients with RM-NPC who failed first-line platinum-based chemotherapy with prior use of anti-PD-1 monoclonal antibody. 10 patients were enrolled in the first phase. If there were no more than 2 effective cases were, the trial would be terminated. Otherwise, the second phase would be entered, and the number of patients enrolled in the second phase would be increased to 22. If there were no more than 7 effective cases (including the first phase), the trial would be terminated. Patients were enrolled for 1 year and followed up for 2 years. Considering the 10% loss to follow-up rate, 25 patients were enrolled in cohort 2.

In order to make more patients will benefit from this trial and continue to evaluate stability and reliability of ORR and its 95% (CI) as the sample size increased, the principal investigator applied to the Research Ethics Committee of Sun Yat-sen University Cancer Center to amend the protocol and expanded the sample size, including 13 additional patients in the cohort 1 and 7 in the cohort 2.

(1) On January 31, 2021, the protocol was changed to include 13 more cohort 1 patients and 7 additional cohort 2 patients, for a total of 40 cohort 1 patients and 32 cohort 2 patients. Cohort 1(n=40): patients with RM-NPC who had failed first-line platinum-based chemotherapy and had not been treated with anti-PD-1 monoclonal antibody.

	(2) Cohort 2(n=32): patients with RM-NPC who failed first-line platinum-based chemotherapy and continued to progress after treatment with anti-PD-1 monoclonal antibody.
Data exclusions	None
Replication	Replication was not applicable as this was a clinical study with unique patient samples.
Randomization	This was a single arm phase 2 study
Blinding	This was a single arm, open label phase 2 study.

Reporting for specific materials, systems and methods

We require information from authors about some types of materials, experimental systems and methods used in many studies. Here, indicate whether each material, system or method listed is relevant to your study. If you are not sure if a list item applies to your research, read the appropriate section before selecting a response.

Materials & experimental systems

n/a	Involved in the study	
☐	☒	Antibodies
☒	☐	Eukaryotic cell lines
☒	☐	Palaeontology and archaeology
☒	☐	Animals and other organisms
☐	☒	Clinical data
☒	☐	Dual use research of concern
☒	☐	Plants

Methods

n/a	Involved in the study	
☒	☐	ChIP-seq
☒	☐	Flow cytometry
☒	☐	MRI-based neuroimaging

Antibodies

Antibodies used

The antibodies used for IHC staining are CD4 (ZM-0418, ZSbio, Clone: EP204); CD19 (ZM-0038, ZSbio, Clone: UMAB103); CD8 (ZA-0508, ZSbio, Clone: SP16); CD3 (ZM-0417 ZSbio Clone: LN10); Eomes (ab183991, Abcam, 1:200); a-SMA (ab7817, Abcam, 1ug/mL); KDR (ab2349, Abcam, 1:100); PD-L1 (ab205921, Abcam, 2ug/mL); CD31(ab28364, Abcam, 1:50); IL8 (94407T, Cell Signaling Technology, 1:100); VEGFA (ab52917, Abacm, 1:100); c-KIT (ab32363, Abcam, 1:400); SRC (ab109381,Abacam, 1:400).

Validation

Anti- human CD4 (ZM-0418, ZSbio, Clone: EP204) and Anti- human CD19 (ZM-0038, ZSbio, Clone: UMAB103): Jiang-Ping Li, Qian Zhong et al. PD-1+CXCR5-CD4+ Th-CXCL13 cell subset drives B cells into tertiary lymphoid structures of nasopharyngeal carcinoma. J Immunother Cancer. 2021 Jul;9(7): e002101. doi: 10.1136/jitc-2020-002101. PMID: 34253636 PMCID: PMC8276302.

Anti- human CD8 (ZA-0508, ZSbio, Clone: SP16): RRID: AB_2890107.

Anti- human CD3 (ZM-0417 ZSbio Clone: LN10): RRID: AB_2890105.

Anti- human Eomes (ab183991, Abcam): RRID: AB_2721040. Morandell J et al. Cul3 regulates cytoskeleton protein homeostasis and cell migration during a critical window of brain development. Nat Commun. 2021 May 24;12(1):3058. doi: 10.1038/s41467-021-23123-x. PMID: 34031387, PMCID: PMC8144225

Anti- human-SMA (ab7817, Abcam): RRID: AB_262054. Rafat M, et al. Bioengineered corneal tissue for minimally invasive vision restoration in advanced keratoconus in two clinical cohorts. Nat Biotechnol 41:70-81 (2023). PMID: 35953672, PMCID: PMC9849136 DOI: 10.1038/s41587-022-01408-w.

Anti- human KDR (ab2349, Abcam): RRID: AB_302998. Bersani-Amado LE, et al. Prostaglandin E1 prevents histopathological changes improving renal function in experimental nephropathy induced by renal microembolism. Int J Clin Exp Pathol. 2020 Jul 1;13(7):1624-1632. PMID: 32782681, PMCID: PMC7414467.

Anti- human PD-L1 (ab205921, Abcam): RRID: AB_2687878. Pathania AS, et al. miR-15a and miR-15b modulate natural killer and CD8+T-cell activation and anti-tumor immune response by targeting PD-L1 in neuroblastoma. Mol Ther Oncolytics. 2022 Mar 31; 25: 308-329. doi: 10.1016/j.omto.2022.03.010. PMID: 35663229, PMCID: PMC9133764

Anti- human CD31(ab28364, Abcam): RRID: AB_726362. Flanagan DJ, et al. Epithelial TGFβ engages growth-factor signalling to circumvent apoptosis and drive intestinal tumourigenesis with aggressive features. Nat Commun. 2022 Dec 7;13(1):7551. doi: 10.1038/s41467-022-35134-3. PMID: 36477656, PMCID: PMC9729215

Anti- human IL8 (94407T, Cell Signaling Technology): Zhihua Ye et al. PCDH1 promotes progression of pancreatic ductal adenocarcinoma via activation of NF-κB signalling by interacting with KPNB1. Cell Death Dis. 2022 Jul 21;13(7):633. doi: 10.1038/s41419-022-05087-y. PMID: 35864095, PMCID: PMC9304345.

Anti- human VEGFA (ab52917, Abacm): RRID: AB_883427. Sivaraj KK et al. Mesenchymal stromal cell-derived septoclasts resorb cartilage during developmental ossification and fracture healing. Nat Commun. 2022 Jan 28;13(1):571. doi: 10.1038/s41467-022-28142-w. PMID: 35091558, PMCID: PMC8799643.

Anti- human c-KIT (ab32363, Abcam): RRID: AB_731513. Harding K et al. C-kit, flt-3, PDGFR-β, and VEGFR2 expression in canine adrenal tumors and correlation with outcome following adrenalectomy. Can J Vet Res. 2021 Oct;85(4):279-284. PMID: 34602732.

Anti- human SRC (ab109381, Abcam): RRID: AB_10865528. Morii M et al. Src Acts as an Effector for Ku70-dependent Suppression of Apoptosis through Phosphorylation of Ku70 at Tyr-530. J Biol Chem. 2017 Feb 3;292(5):1648-1665. doi: 10.1074/jbc.M116.753202. PMID: 27998981, PMCID: PMC5290942.

Clinical data

Policy information about clinical studies

All manuscripts should comply with the ICMJEguidelines for publication of clinical research and a completedCONSORT checklist must be included with all submissions.

Clinical trial registration	ClinicalTrial.gov, NCT04547088 and NCT04548271.
Study protocol	Information regarding the study protocol can be found at clinicaltrials.gov
Data collection	Date was collected in Sun Yat-sen University Cancer Center. The first and last patients were officially enrolled in cohort 1 on September 8, 2020, and August 30, 2021, respectively. In cohort 2, patients were enrolled beginning on November 2th, 2020, and ending on September 7th, 2021. Date collection was the responsibility of the clinical study staff at the site under the supervision of the site Investigator. The study eCRF was the primary data collection instrument for the study. The incestigator had to ensure the accuracy, completeness, legibility, and timeliness of the data reported in the CRFs and all other required reports.
Outcomes	The primary endpoint was the proportion of patients achieving an objective response according to RECIST version 1.1, which included patients with measurable disease who had a complete or partial response. Secondary endpoints were progression-free survival, duration of response, proportion of disease control, and safety. Progression-free survival was defined as the interval from the start of treatment to disease progression or death for any cause (whichever occurred first) or the last progression-free survival assessment for patients alive without progression. Duration of response was assessed in patients who achieved a response and was defined as the time from the date of the first documented response until the date of documented progression or death from any cause. Disease control was defined as the proportion of patients who achieved complete response, partial response or stable disease. The overall survival was defined as the time from treatment initiation to death for any reason.
